# Ribozyme‐Catalyzed Site‐Specific Labeling of RNA Using *O* ^6^‐alkylguanine SNAP‐Tag Substrates

**DOI:** 10.1002/anie.202500257

**Published:** 2025-04-24

**Authors:** Manisha B. Walunj, Carolin P. M. Scheitl, Tina Jungnickel, Claudia Höbartner

**Affiliations:** ^1^ Institute of Organic Chemistry Julius‐Maximilians‐Universität Würzburg Am Hubland 97074 Würzburg Germany; ^2^ Center for Nanosystems Chemistry Julius‐Maximilians‐Universität Würzburg Theodor‐Boveri‐Weg 97074 Würzburg Germany

**Keywords:** Click labeling, Fluorescent labeling, Photocrosslinking, Ribozyme, RNA labeling

## Abstract

Site‐specific modification of RNAs with functional handles enables studies of RNA structure, fate, function, and interactions. Ribozymes provide an elegant way to covalently modify RNA of interest (ROI). Here, we report that the methyltransferase ribozyme MTR1 can be employed as a versatile tool for RNA modification and labeling. Using *O* ^6^‐alkylguanine cofactors, designed in analogy to SNAP‐tag substrates for protein labeling, MTR1 installs various bioorthogonal functional groups at *N* ^1^ of a specific adenosine in the RNA target. In this application of ribozyme‐catalyzed RNA labeling, MTR1 is now called SNAPR. In contrast to the self‐labeling SNAP‐tag, which is appended to the protein of interest, SNAPR is a truly intermolecular RNA catalyst (active in trans). SNAPR assembles with the ROI to the active ribozyme, allowing for the transfer of clickable tags, such as azide and alkyne moieties, as well as photolabile groups or cross‐linkers from the guanine cofactor to the ROI. Moreover, we demonstrate a two‐step approach to attach labels at *N* ^6^ of the target adenosine: first, SNAPR generates *N* ^1^A‐modified RNA, followed by preparative Dimroth rearrangement to produce *N* ^6^A‐modified RNA. We demonstrate this strategy with *p*‐azidobenzyl groups as photocrosslinker to generate covalent RNA–protein conjugates. Overall, this work expands the toolbox for site‐specific RNA modification.

## Graphical abstract

SNAPR is a ribozyme that repurposes *O* ^6^‐benzylguanine SNAP‐tag substrates for site‐specific labeling of RNA with bioorthogonal alkyne and azide functional groups, fluorophores or photocrosslinkers. The *N* ^1^A‐alkylated RNA products were converted to *N* ^6^A‐modified RNA by Dimroth rearrangement. RNA‐catalyzed fluorescent labeling was shown for tRNA, and protein–RNA photocrosslinking was demonstrated for an m^6^A reader protein.

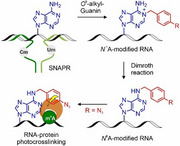

RNA is a key molecule involved in the regulation of numerous cellular processes. To unveil its many functions, structures, and dynamics, methods for the site‐specific functionalization of RNAs are in high demand.^[^ ^1^, ^2^, ^3^ ^]^ Although well‐established solid‐phase synthesis is often the method of choice,^[^ ^4^ ^]^ its lengths limitations as well as incompatibility with certain chemically labile functional groups motivates the development of alternatives. Enzymatic synthetic methods are on the rise for the incorporation of modified nucleotides by engineered polymerases,^[^ ^5^, ^6^, ^7^ ^]^ whereas enzymatic ligation protocols enable the assembly of shorter fragments.^[^ ^8^, ^9^, ^10^ ^]^ Enzymes are also frequently used for the postsynthetic modification of RNAs.^[^ ^11^ ^]^ Examples include the repurposing of tRNA modifying enzymes such as tRNA guanine transglycosylase (TGT)^[^ ^12^ ^]^ or agmatidine synthase,^[^ ^13^ ^]^ and a series of natural or engineered methyltransferases have been employed together with synthetic SAM analogues for the attachment of fluorophores,^[^ ^14^ ^]^ photocrosslinkers,^[^ ^15^ ^]^ or tags for click labeling.^[^ ^16^ ^]^

Recent developments in the field of ribozymes (and deoxyribozymes) paved the way for new approaches for site‐specific RNA modification and labeling.^[^ ^17^, ^18^, ^19^, ^20^, ^21^, ^22^ ^]^ Earlier examples targeted the 2′‐OH group of specific adenosines via formation of phosphodiester^[^ ^23^, ^24^, ^25^, ^26^ ^]^ or phosphonomonoester^[^ ^27^ ^]^ bonds with fluorescently labeled nucleotides, as demonstrated for example on rRNAs^[^ ^27^ ^]^ and mRNAs.^[^ ^28^ ^]^ Alkylation of an internal guanosine at *N* ^7^ was achieved with fluorescein‐ or biotin‐iodoacetamide^[^ ^29^, ^30^ ^]^ or epoxides.^[^ ^31^ ^]^ The programmable RNA‐catalyzed modification of the adenine nucleobase in variable sequence contexts became feasible with the discovery of the methyltransferase ribozyme MTR1^[^ ^32^ ^]^ and the SAM‐analogue utilizing ribozyme SAMURI.^[^ ^33^ ^]^

MTR1 catalyzes the site‐specific methylation of adenosine at position *N* ^1^ using *O* ^6^‐methylguanine as cofactor, resulting in the important natural modified nucleotide m^1^A. Because MTR1 originated from an in vitro selection experiment using biotinylated *O* ^6^‐benzylguanine (BG‐biotin), it is expected that the application of the ribozyme can be expanded in analogy to the well‐established SNAP‐tag for protein labeling,^[^ ^34^ ^]^ but now repurposing SNAP‐tag substrates for the modification of RNA.

Our earlier work revealed the structure of MTR1 and identified two key nucleotides in the active site, C12 and U42.^[^ ^35^, ^36^ ^]^ When these two nucleotides in a bimolecular (split) version of the ribozyme were simultaneously replaced by the corresponding 2′‐*O*‐methyl nucleotides, Cm12 and Um42 (also known as MTR1m2), the methyl transfer rate increased by a factor of 15 compared to the unmodified split ribozyme. The ribozyme was also crystallized after reaction with *O* ^6^‐(4‐aminomethyl‐benzyl)guanine (BG‐NH_2_) as the cofactor, and the structure suggested that further modification of the benzyl group should be tolerated.^[^ ^35^ ^]^

Here, we explored the cofactor scope of MTR1 and establish SNAPR as new ribozyme‐catalyzed RNA labeling platform. We examined the reactivity of MTR1m2 with a library of *O* ^6^‐alkylguanine cofactors, which were prepared by established synthetic strategies (Figure 1).^[^ ^34^ ^]^ Activation of 6‐chloro‐2‐aminopurine (**1**) with *N*‐methylpyrrolidin to **2** was followed by nucleophilic aromatic substitution with a respective alcohol (Figure 1b and Table S1). This resulted in *O* ^6^‐propargylguanine (prop^6^G; **3**) and *O* ^6^‐6‐azidohex‐2‐ynylguanine (azihex^6^G; **4**), which carried the functional group directly at the *O* ^6^ position of guanine. All other cofactors **5**–**10** were synthesized as benzylguanine (BG) derivatives with the functional groups attached at the *para*‐position of the phenyl ring, as previously shown for SNAP‐tag substrates.^[^ ^34^, ^37^, ^38^, ^39^, ^40^ ^]^ The BG cofactors **5**–**7** (Figure 1c) carried alkyne or azide groups for subsequent click labeling by Cu‐catalyzed or strain‐promoted azide–alkyne cycloadditions, and cofactors **8**–**10** contained aryl azides or benzophenone as photocrosslinkers. Cofactors **7** ^[^ ^41^ ^]^ and **10** ^[^ ^42^ ^]^ were obtained from BG‐NH_2_ via amide coupling to DBCO‐NHS ester and formation of the azidonaphthalimide (AzNP) from the corresponding anhydride, respectively. It should be noted that compounds **3**, **5**, **6**, and **7**, are available from commercial suppliers as SNAP tag substrates that can now also be used for RNA labeling.

**Figure 1:**
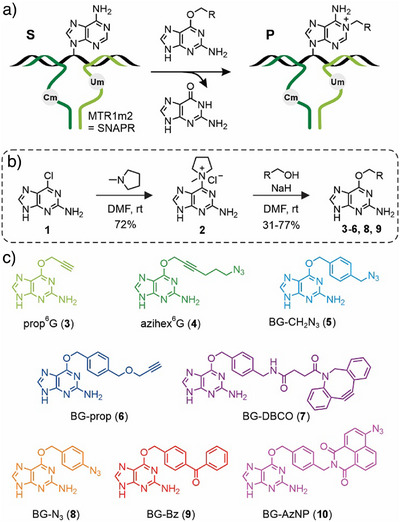
a) Schematic depiction of MTR1m2 (assembled from two strands shown in dark and light green) hybridized to the target RNA substrate (S, black). The alkyl group is transferred to the *N* ^1^ position of the modification site adenosine within the target RNA. Cm and Um represent the 2′‐*O*‐methylated nucleosides Cm12 and Um42. b) General reaction scheme for the synthesis of *O* ^6^‐alkylguanine cofactors. c) Chemical structures for all guanine cofactor derivatives used in this study. prop, propargyl; azihex, 6‐azidohex‐2‐ynyl; BG, benzyl guanine; DBCO, dibenzocyclooctyne; AzNP, azidonaphthalimide; Bz, benzoyl.

Each of these *O* ^6^‐alkylguanine cofactors was tested as ribozyme substrate and shown to yield alkylated RNAs. The kinetic assays were performed with the bimolecular ribozyme variant MTR1m2 due to its high reaction rate and low Mg^2+^ demand (5 mM Mg^2+^, pH 6.0, 25 °C). The reactions were performed using a fluorescently labeled 14‐nt RNA substrate (S, sequence R1 see Supporting Information, Table S2) and the formation of the alkylated product (P) was observed by denaturing PAGE (Figure 2a). The BG derivatives **5–8** yielded ≈90% alkylated RNA within one hour (Figure 2b). The reactions of prop^6^G (**3**) and axihex^6^G (**4**) were somewhat slower and needed 3–5 h for complete conversion to the product. With the bulkier cofactors **9** and **10**, more than 90% alkylated RNA was produced after 24 h. The observed rate constants for all cofactors are summarized in Figure 2c (additional gel images in Figure S1). We also note that diverse *O* ^6^‐alkylguanine cofactors can be used with the unmodified monomolecular MTR1, but the reactions are generally slower (Figure S1). Additional conceptual advantages of using the split SNAPR version are that the active ribozyme assembles only in the presence of the target RNA substrate, i.e., with both fragments finding a correct hybridization partner.^[^ ^43^ ^]^ The short binding arms do not tolerate mismatches close to the target site and the shorter SNAPR fragments have a smaller chance for intramolecular misfolding than a full‐length ribozyme. Furthermore, SNAPR is an efficient multiple turnover ribozyme, resulting in 90% labeled product after 5 h with only 10 mol% ribozyme (Figure S2). Thus, following the promising kinetic scale experiments, each of the SNAPR reactions was also performed on preparative scale using RNA substrate R2 (same sequence as R1, but no fluorescent label). The reactions were also monitored by anion‐exchange HPLC (Figure S3), and the products were isolated by PAGE and characterized by high‐resolution electrospray ionization mass spectrometry (HR‐ESI‐MS) (Table S4).

**Figure 2:**
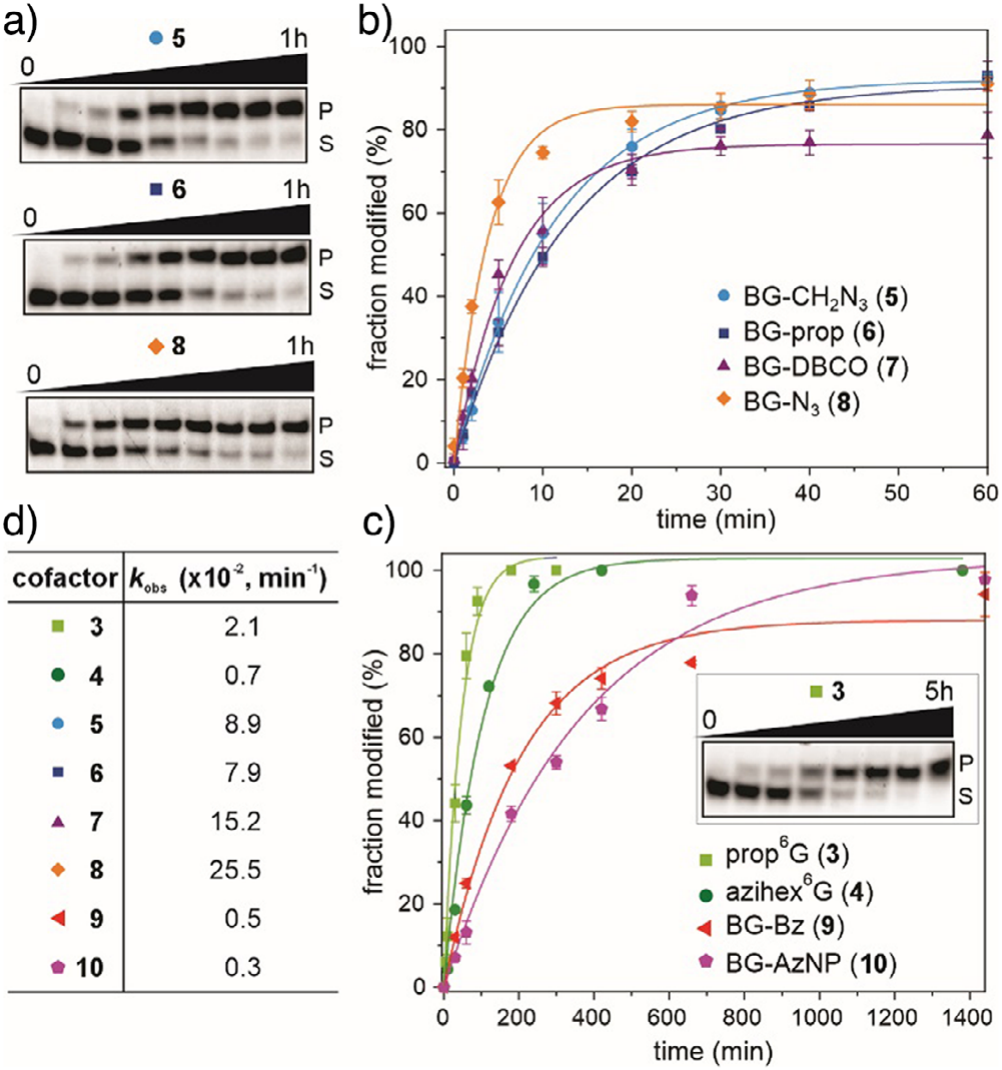
a) Representative PAGE images of the kinetic assays for cofactors **5**, **6,** and **8** from two independent experiments. 1 µM 3′‐fluorescein labeled RNA R1 (5′‐CCACUGAGAGCUUC‐NH_2_‐3′), 10 µM MTR1m2 (R3 + R4, Table S2), 100 µM cofactor, 5 mM MgCl_2_, pH 6.0, 25 °C, timepoints: 0, 1, 2, 5, 10, 20, 30, 40, and 60 min. b) and c) Kinetics of MTR1m2 with all cofactors used in this study. The data was fit using a pseudo‐first‐order curve fit. d) Summary of *k*_obs_ values for graphs shown in (b) and (c).

To demonstrate the general application of SNAPR for fluorescent labelling of longer RNAs, we designed the two ribozyme fragments with binding arms that target A58 of *E.coli* tRNA‐Asp (Figure 3).^[^ ^44^ ^]^ First, we confirmed the high efficiency of the labeling reaction in the tRNA sequence context on a 13‐nt tRNA fragment (R5) using azide and alkyne cofactors **5** and **6** (Figure 3a,b). The same ribozyme was then used on the 77‐nt full‐length tRNA (Figure 3c). The successful installation of the azide or alkyne handle was confirmed by CuAAC with FAM‐alkyne or FAM‐azide, respectively, which resulted in fluorescent tRNA bands, when the reaction mixture was resolved on PAGE (Figure 3d). Control reactions performed in the absence of either cofactor or dye showed no fluorescent signal. Importantly, no degradation of the tRNA was observed during alkylation or click reactions (Figure S4).

**Figure 3:**
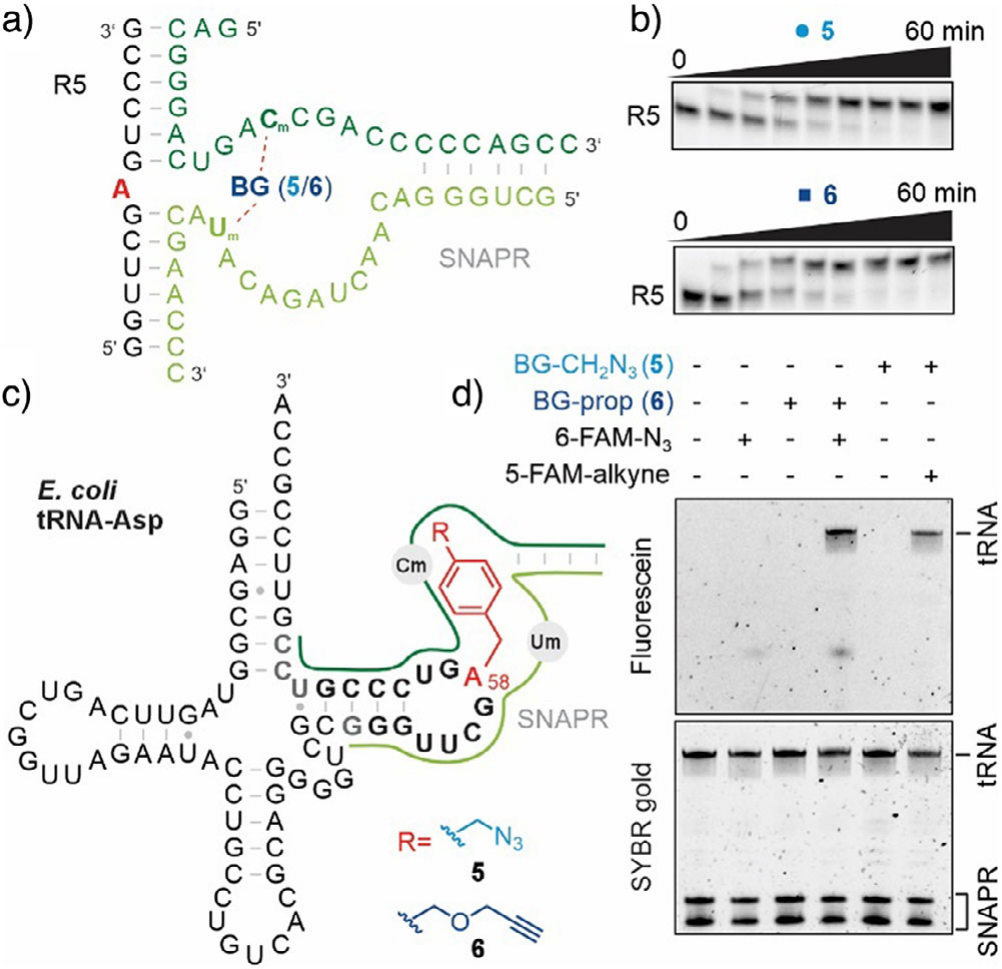
SNAPR‐catalyzed labeling of *E. coli* tRNA‐Asp. a) Sequence of 13‐nt tRNA fragment (R5) and two SNAPR fragments (R6, R7 shown in light and dark green), with binding site and H‐bonding of BG cofactors **5** and **6** to Cm and Um indicated. b) Gel images of labeling kinetics using 1 µM 3′‐fluorescein labeled RNA R5, 10 µM SNAPR (R6 + R7), 100 µM cofactor, 5 mM MgCl_2_, pH 6.0, 25 °C, timepoints: 0, 1, 2, 5, 10, 20, 30, 40, and 60 min. c) Sequence and secondary structure of the tRNA. Modification site A58 is depicted in red. SNAPR indicated by green lines, binding sites on the RNA shown in bold letters. d) CuAAC labeling reaction of *E. coli* tRNA‐Asp modified by SNAPR using cofactors **5** or **6** resolved on denaturing PAGE. Conditions: 50 µM 6‐FAM‐N_3_ or 5‐FAM‐alkyne, 0.5 mM CuBr, 1 mM TBTA, 37 °C, 3 h. The gel was imaged in the fluorescein‐channel (top) to detect the labeled tRNAs. Then it was stained with SYBR gold (bottom) to visualize the tRNA and the two SNAPR fragments.

Alkylation of adenosine at *N* ^1^ by SNAPR generates a positively charged nucleobase with a blocked Watson–Crick base‐pairing face. However, for some applications it may be desirable to attach the alkyl group at *N* ^6^ instead of *N* ^1^ of adenosine. It is well known that m^1^A can be converted to m^6^A by Dimroth rearrangement under alkaline conditions^[^ ^32^, ^45^, ^46^ ^]^ or in the presence of nucleophiles, such as 4‐nitrothiophenol (NTPh) at pH 6.0.^[^ ^47^ ^]^ Interestingly, for several of the alkylation products generated by SNAPR with cofactors **3**–**10**, a minor fraction of *N* ^6^‐alkylated product was already observed after extraction from polyacrylamide gels (Figure S3, S5), suggesting that Dimroth rearrangement occurs rather easily for *N* ^1^‐benzylated adenosines. Thus, we investigated the efficiency of forming *N* ^6^‐alkylated RNAs directly from the SNAPR products (Figure 4). Traditional OH^−^‐promoted conversion at pH 10 (60 °C, 1 h) resulted in only about 50% *N* ^6^A‐modified RNA products, but treatment with NTPh at pH 6 yielded more than 80% conversion (Figure 4b and Table S5), as observed by a shift in retention time by anion‐exchange HPLC. In addition, the formation of *N* ^6^‐alkylation products was also confirmed after digestion and LCMS analysis using synthetic references. Although the analysis is well established for m^1^A and m^6^A, which show a large difference in RP‐HPLC retention times (Figure S5), the analysis of the experiments with the more hydrophobic benzylated analogues needed additional attention. For example, the benzophenone‐modified nucleoside *N* ^6^‐(4‐benzoylbenzyl) adenosine was largely extracted into the organic phase upon workup of the digestion reaction, whereas the minor fraction of *N* ^1^‐(4‐benzoylbenzyl) adenosine stayed in the aqueous phase (Figure S6).

**Figure 4:**
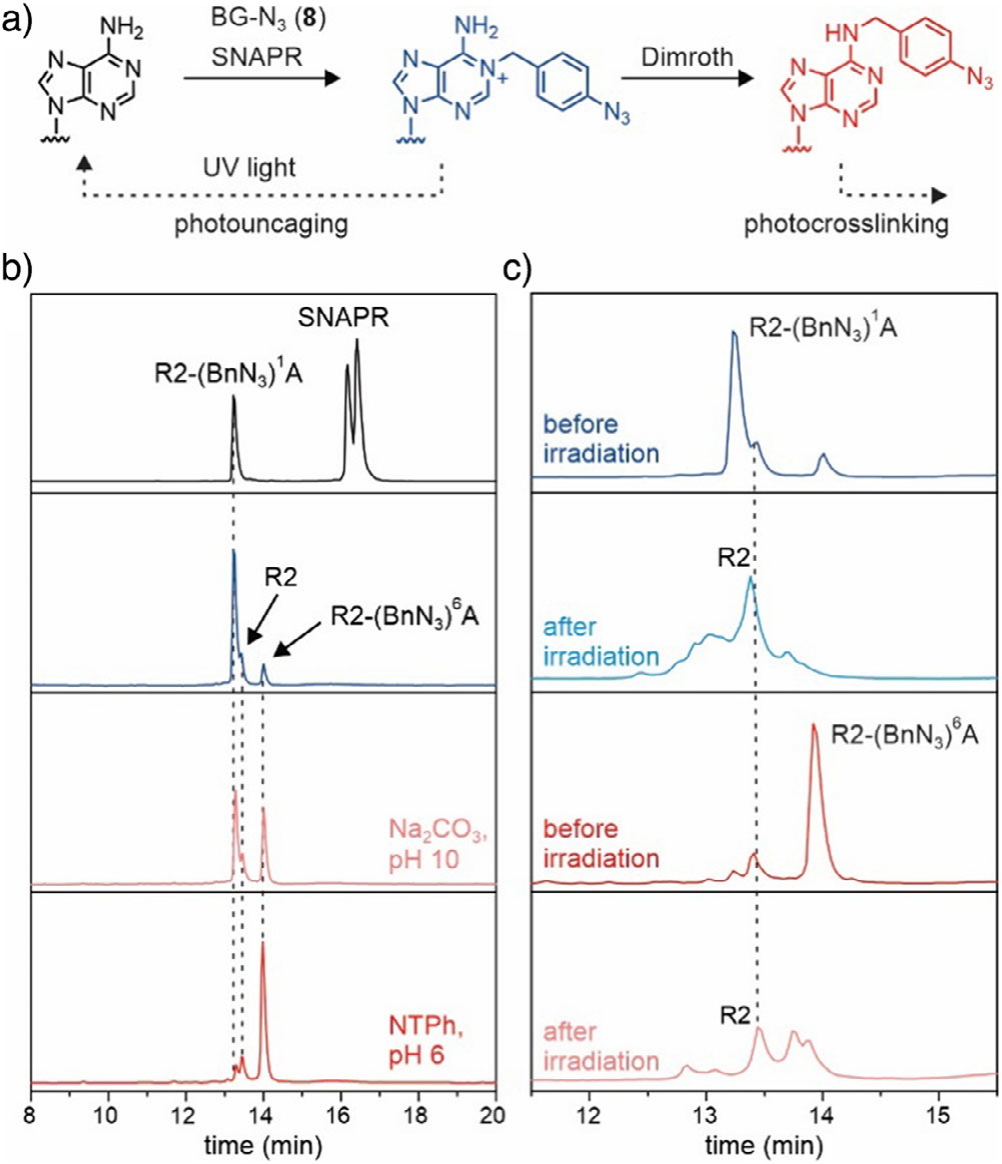
a) Schematic depiction of the generation of (BnN_3_)^1^A‐modified RNA (14‐nt) by SNAPR, subsequent formation of (BnN_3_)^6^A‐modified RNA via Dimroth rearrangement and the generation of uncaged adenosine upon irradiation. b) Anion‐exchange HPLC profile of the transalkylation reaction using SNAPR and the cofactor BG‐N_3_ (**8**) before (black) and after (blue) PAGE purification as well as the products upon Dimroth rearranged using Na_2_CO_3_ (pH 10), 60 °C, 1 h (pink) or 4‐nitrothiophenol (NTPh), pH 6, 60 °C, 9 h (red). c) Anion‐exchange HPLC profile for (BnN_3_)^1^A and (BnN_3_)^6^A‐modified RNA before and after irradiation at 312 nm for 5 min.

For the *p*‐azidobenzyl‐modified RNAs obtained with cofactor **8**, we note another interesting observation. In short, we found that the *p*‐azidobenzyl group functions as the expected photocrosslinker^[^ ^48^ ^]^ when it is attached to *N* ^6^ of adenosine, whereas UV irradiation of the *N* ^1^ isomer resulted in unexpected release of unmodified RNA as the predominant product (Figures 4c and S7). Thus, the *p*‐azidobenzyl group at *N* ^1^ of adenosine can be considered as a photolabile caging group for the Watson–Crick face of adenosine. This finding is just the opposite of what has been reported for the *o*‐nitrobenzyl group attached to *N* ^1^ or *N* ^6^ of adenosine, where only the *N* ^6^ isomer was cleaved upon UV irradiation, whereas the *N* ^1^ isomer was unreactive.^[^ ^49^ ^]^ Although best known as crosslinker via photoinduced formation of aryl nitrene, the *p*‐azidobenzyl group has previously also been used as a caging group, for example, in carboxylic acid esters; photoreduction of the azide to the amine resulted in release of the carboxylic acid.^[^ ^50^, ^51^ ^]^ Here, for *p*‐azidobenzyl adenosines, photoactivation of the azide results in release of nitrogen, and the resulting nitrene is either directly trapped or undergoes ring expansion and reaction with a nucleophile, or the azide is transformed to an amine that undergoes 1,6‐elimination resulting in cleavage from the adenosine. The HPLC traces (Figure 4c) and mass spectra (Figure S7) reflect the multiple possible reaction products. Similar results were obtained with synthetic *N* ^1^‐ and *N* ^6^‐*p*‐azidobenzyladenosine nucleoside standards (Figure S8), showing adenosine as the major product released from (BnN_3_)^1^A. The positive charge in *N* ^1^‐(*p*‐azidobenzyl)‐adenosine likely assists the elimination reaction. Indeed, similar observations for decaging of positively charged *N* ^1^‐adenosine or *N* ^7^‐guanosine derivatives were recently reported for benzophenone‐modified nucleosides.^[^ ^52^ ^]^

To demonstrate the use of SNAPR for installation of a photocrosslinker to generate covalent RNA–protein conjugates, we chose m^6^A‐containing RNA and attempted the covalent trapping of an m^6^A‐reader protein. m^6^A is the most extensively studied epigenetic modification and is dynamically regulated by writer and eraser proteins; reader proteins including the YTH family are responsible for binding m^6^A‐marked RNAs.^[^ ^53^, ^54^ ^]^ First, we used SNAPR to install the *p*‐azidobenzyl group in the vicinity of an m^6^A‐modified DRACH motif (D = A, G, or U; R = G or A; H = A, C, or U) and employed the optimized Dimroth rearrangement conditions to produce *N* ^6^‐modified RNA (Figures 5 and S9). Next, we assessed the binding of double‐modified RNA to YTHDF2 by fluorescence anisotropy,^[^ ^55^ ^]^ and found that the benzyl group outside the DRACH motif does not disturb the recognition of m^6^A (Figure 5c). In contrast, a benzyl group in place of m^6^A within the DRACH motif disrupted protein binding. After addition of YTHDF2, the RNA–protein complex solution was irradiated at 312 nm and the sample was resolved on SDS‐PAGE. A slower‐migrating band revealed the covalent RNA–protein conjugate, which was not present in the negative controls (Figure 5d). Further, a random RNA containing the BnN_3_ photocrosslinker or addition of bovine serum albumin (BSA) instead of YTHDF2 showed no detectable photocrosslinking reaction (Figure S10). Only for the m^6^A containing, non‐BnN_3_ control RNA (R10), a very faint band was detectable, resembling the noncovalent m^6^A‐RNA protein complex.

**Figure 5:**
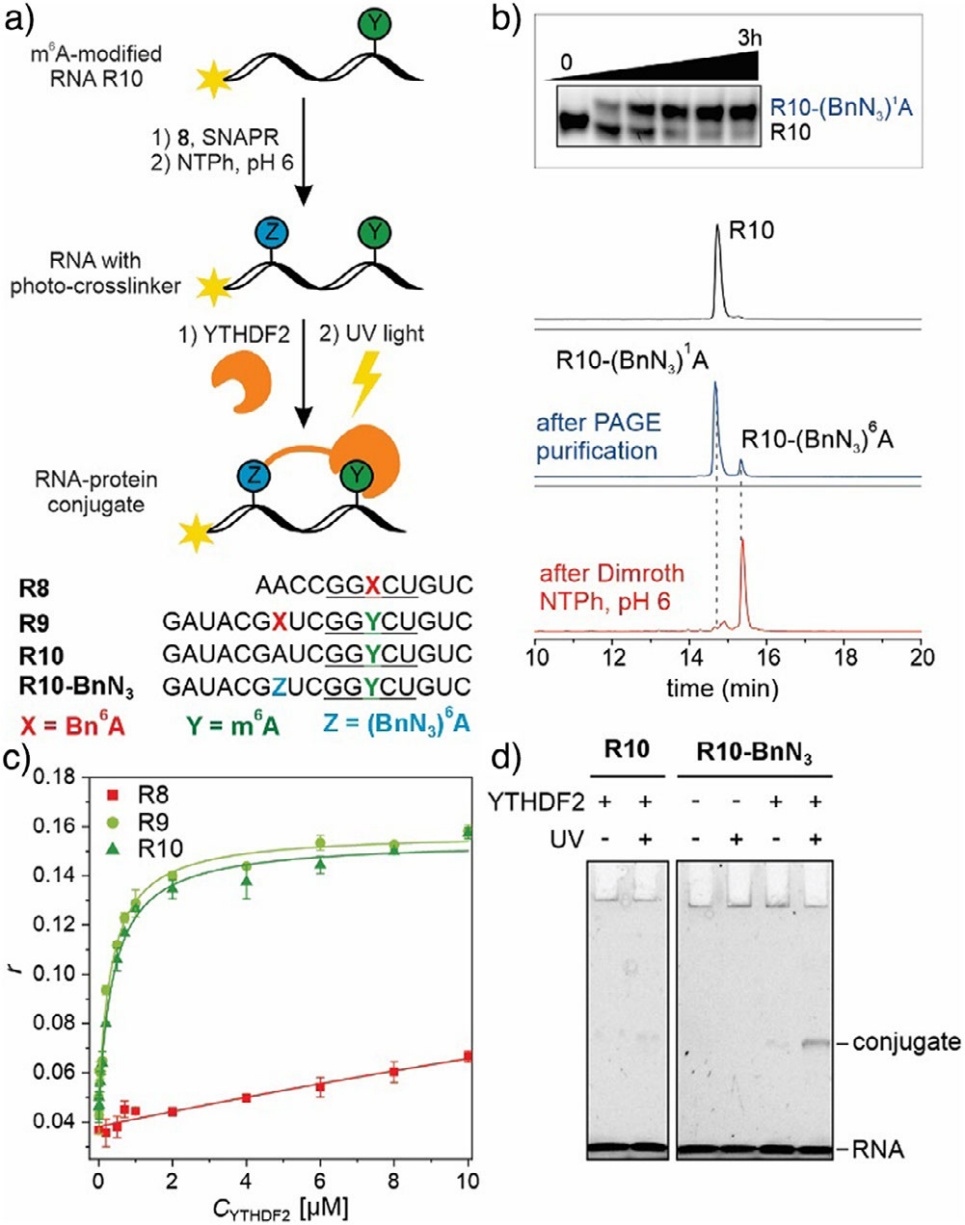
a) Schematic representation of *p*‐azidobenzyl (BnN_3_) modification and photocrosslinking between the YTHDF2 reader protein and BnN_3_‐modified m^6^A‐containing RNA. b) Analysis of SNAPR‐catalyzed labeling of R10 (17‐nt) by PAGE (top) and Dimroth reaction by anion‐exchange HPLC. c) Fluorescence anisotropy binding curves of the m^6^A reader protein YTHDF2 in complex with FAM‐labelled RNAs R8, R9, and R10. Data points were collected as triplicates and are presented as mean ± SEM. d) Analysis of RNA–protein crosslinking on SDS‐PAGE reveals a band for the covalently crosslinked RNA–protein conjugate. Photocrosslinking conditions: 0.2 µM of RNA (2 pmol), 2 µM YTHDF2, 30 mM Tris‐HCl (pH 7.5), 120 mM NaCl, irradiation at 312 nm for 5 min.

In summary, we established SNAPR as new RNA labeling platform that employs the MTR1 ribozyme in combination with a variety of *O* ^6^‐alkylguanine cofactors for the fast and simple postsynthetic modification of RNAs. SNAPR enables the installation of different clickable bioorthogonal or light‐sensitive tags on various target RNAs including long or structured RNAs of interest, such as tRNAs. We demonstrated that SNAPR can be employed for fluorescent labeling, RNA caging as well as RNA–protein crosslinking, thus complementing existing methods for nucleic acid modification. This work also showed the postsynthetic introduction of functional groups, such as *p‐*azidobenzyl, which are incompatible with phosphoramidite chemistry in solid‐phase synthesis. Together with almost quantitative Dimroth rearrangement under mild conditions, this work expands the fields of applications of the MTR1 ribozyme and of SNAP‐tag substrates for site‐specific RNA labeling and functionalization at *N* ^6^ of adenosine. Thus, SNAPR is a fully RNA‐based alternative to engineered MTase enzymes that are used in combination with synthetic SAM derivatives to label RNA at various positions.^[^ ^14^, ^15^, ^16^ ^]^ MTases are specific for certain nucleotides in defined sequence or structural contexts, such as for example, the DRACH motif or mRNA cap structure. In contrast, SNAPR can be directed to internal adenosines in RNA of interest by Watson–Crick complementarity of the binding arms. SNAPR uses bioorthogonal guanine‐based cofactors that do not show any known cross reactivity with MTases or other RNA‐modifying enzymes. For future applications, we imagine that SNAPR can be expanded further using tailored cofactors to install functionalities beyond those investigated within this study. These results may also inspire the search for new RNA alkylating ribozymes that target nucleosides beyond adenosine to further expand the toolbox of RNA labeling ribozymes.

## Supporting Information

Materials and methods, supporting Tables S1–S5, supporting Figures S1–S11, and NMR spectra.

## Conflict of Interests

The authors declare no conflict of interest.

## Supporting information



Supporting information

## Data Availability

The data that support the findings of this study are available in the Supporting Information of this article.
