# Supplementary material for: Ribozyme‐Catalyzed Site‐Specific Labeling of RNA Using O 6‐alkylguanine SNAP‐Tag Substrates
Source: Angew Chem Int Ed Engl. 2025 Apr 24;64(26):e202500257. doi: 10.1002/anie.202500257 (PMC12184302; doi:10.1002/anie.202500257)
Supplement: Supplementary file 1 — Supporting information [file ANIE-64-e202500257-s001.pdf]

Ribozyme-catalyzed site-specific labeling of RNA using O^6-alkylguanine SNAP-tag substrates

Manisha B. Walunj, Carolin P. M. Scheitl, Tina Jungnickel, and Claudia Höbartner*

Institute of Organic Chemistry, University of Würzburg, Am Hubland, Germany

Table of Contents

1 General information 2

1.1 Materials 2

1.2 NMR spectroscopy and mass spectrometry 2

2. Synthesis 2

2.1 1-(2-Amino-9H-purin-6-yl)-1-methylpyrrolidin-1-ium chloride (**2**): 2

2.2 Synthesis of alcohols 3

2.3 Synthesis of *O*^{6}-(4-aminomethylbenzyl)guanine = BG-NH_2(**25**) 6

2.4 Synthesis of DBCO-NHS ester (**33**) 7

2.5. Synthesis of 6-azido-1H,3H-benzo[de]isochromene-1,3-dione (**35**) 11

2.6 General procedure for synthesis of *O*^{6}-alkyl guanine cofactors (**3**–**6**, **8**, **9**) (Table S1). 11

2.7 Synthesis of *N*-(4-(((2-amino-9*H*-purin-6-yl)oxy)methyl)benzyl)-4-(11,12-didehydrodi-benzo[b,f]azocin-5(6*H*)-yl)-4-oxobutanamide (**7**) 12

2.8 Synthesis of 2-(4-(((2-amino-9H-purin-6-yl)oxy)methyl)benzyl)-6-azido-1H-benzo 12

[de]isoquinoline-1,3(2H)-dione (**10**) 12

2.9 Spectral data for *O*^{6}-alkyl guanine cofactors (**3**–**10**) 12

2.10 Synthesis of N^1 and N^6 modified adenosine standards 15

3. Solid phase synthesis, deprotection and purification of RNA oligonucleotides 20

4. In vitro transcription reaction of unmodified RNA (tRNA and ribozymes) 20

5. Fluorescent labeling of oligonucleotides 20

5.1 3'-end labeling of 3'-amino-terminated RNAs using NHS-ester 20

5.2 3'-end labeling of substrate RNA using periodate glycol oxidation 20

5.3 5'-end labeling of 5'-alkyne-terminated RNA using CuAAC 21

6. Kinetic assays of RNA-catalyzed RNA alkylation reactions 21

7. Preparative RNA alkylation using MTR1m2 21

8. Dimroth rearrangement 21

9. LC-MS anaylsis of modified RNA substrates 22

10. Fluorescence anisotropy 22

11. Supporting Tables 22

12. Supporting Figures 25

13 NMR spectra 34

14 HR-ESI-MS spectra 46

References 53

1 General information

1.1 Materials: All standard chemicals and solvents were purchased from commercial suppliers. Organic solvents were used in “pro analysis” or “for synthesis” quality without further purification. Solvents for extraction and column chromatography were purchased in technical quality and distilled prior to use. Dry solvents (dichloromethane, THF, DMF) were obtained from a solvent purification system (SPS). Nanopure water was obtained from a Sartorius Arium® pro ultrapure water system. Thin layer chromatography (TLC) was performed on silica gel pre-coated aluminum plates (Alugram SIL G/UV254, Macherey-Nagel). Visualization was accomplished by irradiation with UV light at short wavelength (254 nm). Column chromatography was carried out on silica gel (Kieselgel 60, Merck, 0.063 – 0.200 mm). Standard 5'-*O*-DMT-2'-*O*-TOM-protected RNA phosphoramidites for solid phase synthesis as well as standard 5'-*O*-DMT-protected CPG supports (1000 Å, 25-35 µmol/g) were purchased from ChemGenes or Sigma Aldrich.

1.2 NMR spectroscopy and mass spectrometry: NMR spectra were measured on a Bruker Avance III HD 400 spectrometer at 400 MHz or 600 MHz. Chemical shifts (δ) were referenced to the residual solvent signals as internal standards (in ppm; $CDCl_3$: ^1H = 7.26, ^{13}C = 77.16, DMSO: ^1H = 2.50, ^{13}C = 39.52) Coupling constants (J) were reported in Hz. Multiplets were abbreviated as following: s (singlet), d (doublet), t (triplet), q (quartet) and m (multiplet). Spectral assignments were verified by 2D NMR experiments. HR-ESI-MS spectra of the oligonucleotides were recorded on a Bruker micrOTOF-Q III spectrometer in negative ion mode upon direct injection of a 3 µM RNA solution in 44% ACN (v/v) containing 44 mM hexafluoroisopropanol (HFIP) and 3.8 mM triethylamine (TEA), using capillary voltage 3 kV, end plate voltage 2.5 kV, nitrogen nebulizer pressure 0.4 bar, dry gas flow 4 L/min, dry temperature 200 °C, and recorded in the range m/z 200 - 4000. Data evaluation and ion deconvolution were performed by Data analysis software DA 4.2 (Bruker Daltonics).

2. Synthesis

2.1 1-(2-Amino-9H-purin-6-yl)-1-methylpyrrolidin-1-ium chloride (2):

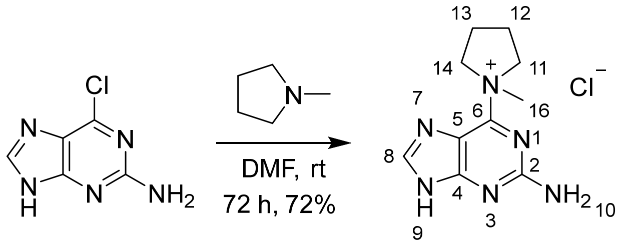

1 **2**

Compound **2** was synthesized according to a procedure reported in literature.[1] 6-Chloroguanine (5.00 g, 29.49 mmol, 1.00 eq.) was dissolved in DMF (250 mL) and stirred for 30 min at 50 °C. The solution was cooled to room temperature. Next, 1-methylpyrrolidine (17.26 mL, 162.19 mmol, 5.5 eq.) was added to the cooled solution and the reaction mixture was stirred for 72 h at room temperature. Acetone was added to ensure complete precipitation. The colourless precipitate was filtered and further washed with acetone and dried in vacuum to obtained the pure product as a white solid (6.5 g, 87%). The product shows blue

fluorescence at longer wavelength (>360 nm) under the UV lamp. Yield and spectral data are in agreement with the previously reported procedure.

^{1}H NMR (400 MHz, DMSO-*d*$_6$) δ (ppm) = 13.46 (s, 1H, H-9), 8.34 (s, 1H; H-8), 7.12 (s, 2H, H-10), 4.65 – 4.54 (m, 2H, -CH_2-N^+-, pyrrolidin), 4.03 – 3.92 (m, 2H, -CH_2-N^+-, pyrrolidin), 3.65 (s, 3H, CH3), 2.29 –2.19 (m, 2H, CH_2-, pyrrolidin), 2.09 – 1.98 (m, 2H, CH_2-, pyrrolidin).

^{13}C NMR (101 MHz, DMSO-*d*$_6$) δ (ppm) = 159.06 (C6), 158.55 (C2), 151.65 (C4), 142.99 (C8), 116.02 (C5), 64.01 (2C, C11, C14), 51.54 (C16), 21.44 (2C, C12, C13).

HR-MS (ESI$^+$): Exact mass calculated for $[C_{10}H_{15}N_6]^+$: 219.1353, found: 219.1358.

2.2 Synthesis of alcohols

2.2.1 6-Azidohex-2-yn-1-ol (12)

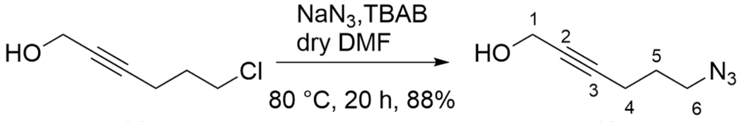

11 **12**

The synthesis was conducted according to a procedure reported in literature.[2] Sodium azide (2.45 g, 37.70 mmol, 9.93 eq.) and tetrabutylammonium bromide TBAB (122 mg, 378 µmol, 0.10 eq.) were added to a solution of 6-chlorohex-2-yn-1-ol **11** (503 mg, 3.79 mmol, 1.00 eq.) in DMF (25 mL). The resulting suspension was stirred at 80 °C for 20 h under nitrogen atmosphere. After cooling to room temperature, distilled H_2O (25 mL) was added and the aqueous phase was extracted with a mixture of *n*-hexane and EtOAc (1:1, 4 x 5 mL, then 1:2, 2 x 42 mL) followed by EtOAc (3 x 30 mL). The combined organic phases were washed with distilled H_2O (5 x 25 mL) and dried over anhydrous $MgSO_4$. Removal of the solvent under reduced pressure afforded **12** as a slightly yellow oil (462 mg, 3.32 mmol, 88%).

^{1}H-NMR (400 MHz, DMSO-*d*$_6$) δ (ppm) = 5.06 (t, *J* = 5.9 Hz, 1H, OH), 4.03 (dt, *J* = 5.9, 2.2 Hz, 2H, $\underline{CH_2}$-OH), 3.41 (t, *J* = 6.8 Hz, 2H, $\underline{CH_2}$-N_3), 2.27 (tt, *J* = 7.0, 2.2 Hz, 2H, H-5), 1.68 (p, *J* = 6.9 Hz, 2H, H-6).

^{13}C-NMR (101 MHz, DMSO-*d*$_6$) δ (ppm) = 82.80 (C4), 81.09 (C2), 49.60 (CH_2-N_3), 49.11 (CH_2-OH), 27.48 (C6), 15.40 (C5).

HR-MS (ESI$^+$): Exact mass calculated for $C_6H_9N_3NaO$ $[M+Na]^+$: 162.06378, found: 162.06451.

2.2.2 (4-(Azidomethyl)phenyl)methanol (14)

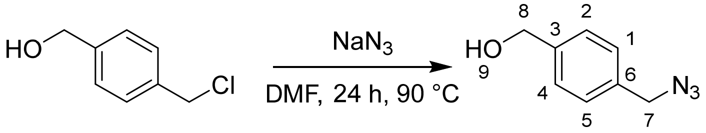

13 **14**

The reaction procedure was modified from a literature known procedure.[3] 4-(Chloromethyl)-benzylalcohol **13** (2.50 g, 15.96 mmol, 1.00 eq.) and sodium azide (10.38 g, 159.60 mmol, 10.0 eq.) were dissolved in 160 mL dry DMF and stirred at room temperature for 24 h. After completion of the reaction almost half the volume of DMF was evaporated, and water (50 mL) was added. The product was extracted with EtOAc. Afterwards the combined organic phases were washed with water (4x100 mL). Further, the product was purified using flash chromatography with 5% MeOH in CH_2Cl_2 which afforded the pure product **14** as a yellow-colored oil.

TLC (CH_2Cl_2); R_f = 0.33.

^{1}H-NMR (400 MHz, DMSO-d_6) δ (ppm) = 7.39 – 7.29(m, 4H, Ar), 5.23 (tt, J = 5.7, 1.5 Hz, 1H, OH), 4.51 (d, J = 5.7 Hz, 2H, $\underline{CH_2}$-OH), 4.41 (s, 2H, $\underline{CH_2}$-N_3) ppm.
^{13}C-NMR (101 MHz, DMSO-d_6) δ (ppm) = 143.48, 135.46, 128.37 (2C), 126.77 (2C), 62.60 ($\underline{CH_2}$-OH), 54.24 ($\underline{CH_2}$-N_3) ppm.
HR-MS (ESI$^+$): Exact mass calculated for $C_8H_9N_3NaO$ $[M+Na]^+$: 186.06378; found: 186.06428.

2.2.3 (4-((Prop-2-yn-1-yloxy)methyl)phenyl)methanol (16)

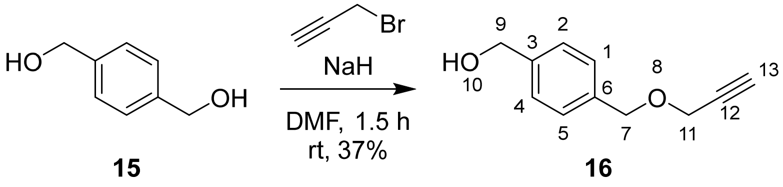

The reaction was performed using a literature known procedure.[1] 1,4-Benzenedimethanol (2.00 g, 14.47 mmol, 1.10 eq.) was dissolved in dry DMF (20 mL) under an argon atmosphere at room temperature. Sodium hydride (400 mg, 16.69 mmol, 1.30 eq.) was added in small portions over 10 min at 0 °C. The reaction mixture was stirred for additional 15 min at 0 °C. Afterwards, propargyl bromide (1.93 g, 13.03 mmol, 1.00 eq.) was added, and the mixture was stirred at room temperature. After 1.5 h the reaction was quenched with cold water. Solvents were removed under reduced pressure. The crude mixture was dissolved in water (50 mL) extracted with CH_2Cl_2 (2 x 50 mL) and further purified by column chromatography (EtOAc /*n*-hexane = 50:50), which afforded the pure product as a colorless oil (37%, 0.84 g).
TLC (EtOAc /*n*-hexane = 50:50); R_f = 0.50
^{1}H-NMR (400 MHz, DMSO-d_6) δ (ppm) = 7.33 – 7.25 (m, 4H), 4.51 – 4.46 (m, 4H), 4.16 (d, J = 2.4, 2H), 3.48 (t, J = 2.4, 1H)
^{13}C-NMR (101 MHz, DMSO-d_6) δ (ppm) = 142.06, 135.85, 127.68 (2C), 126.39 (2C), 80.24, 77.41, 70.61, 62.67, 56.72.
HR-MS (ESI$^+$): Exact mass calculated for $C_{11}H_{12}NaO_2$ $[M+Na]^+$: 199.07295; found: 199.07288.

2.2.4 (4-(Azido)phenyl)methanol (18)

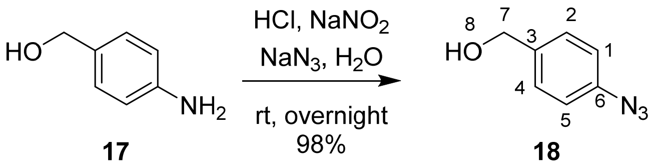

(4-Azidophenyl)methanol was synthesized according to a procedure reported in literature.[4] 4-Aminobenzylalcohol **17** (0.50 g, 4.06 mmol, 1.00 eq.) was dissolved in hydrochloric acid (2.5 mL, 12 M). Sodium nitrite (0.42 mg, 6.09 mmol, 1.50 eq.) was dissolved in water (10 mL) and added dropwise to the reaction mixture within 30 min. The solution was stirred vigorously in ice-cold water. Sodium azide (1.05 g, 16.15 mmol, 4 eq.) was added in portions to the mixture. The resulting solution was stirred at room temperature overnight. After completion of the reaction, the crude mixture was extracted with EtOAc (2 x 10 mL). The organic layer was dried over anhydrous Na_2SO_4 and the crude product was purified by silica gel column chromatography (EtOAc /*n*-hexane = 20:80) to obtained the pure product **18** as a colorless oil (98%, 0.59 g).
TLC (EtOAc /*n*-hexane = 60:40); R_f = 0.57
^{1}H NMR (400 MHz, $CDCl_3$) δ (ppm) = 7.37 – 7.34 (m, 2H, Ar-H), 7.04 – 7.01 (m, 2H, Ar-H), 4.67 (s, 2H, H-7).

^{13}C NMR (101 MHz, $CDCl_3$) δ (ppm) = 139.53, 137.71, 130.42 (2C), 120.01 (2C), 64.42.
HR-MS (ESI$^+$): Exact mass calculated for C_7H_8NO $[M-N_2+H]^+$: 122.06004, found: 122.06054.

2.2.5 4-Hydroxymethyl benzophenone (20)

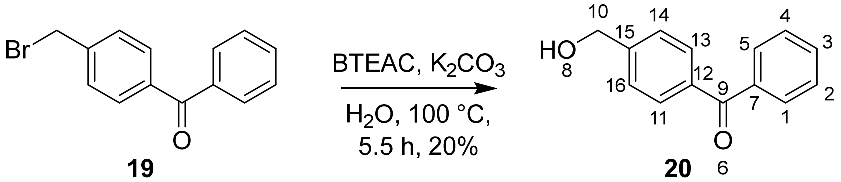

4-Hydroxymethyl benzophenone **20** was synthesized according to a procedure reported in literature.[5] A mixture of 4-bromomethyl benzophenone **19** (3.00 g, 10.90 mmol, 1.00 eq.), K_2CO_3 (4.50 g, 32.70 mmol, 3.00 eq.) and the phase transfer catalyst benzyltriethylammonium chloride BTEAC (273 mg, 1.20 mmol, 0.11 eq.) in water (30 mL) was stirred at 100 °C for 5.5 h. Further, the reaction mixture was extracted with EtOAc (2 x 30 mL) and the organic layer was dried over anhydrous sodium sulfate. The crude product was purified by column chromatography using a gradient based on EtOAc:*n*-hexane to obtain the desired product as a pure white solid. (462 mg, 20%). In addition, dimer formation of the expected product was observed ($C_{28}H_{22}O_3$, 1.20 g 27% R_f = 0.59).
TLC (EtOAc /*n*-hexane = 30:70); R_f = 0.20
^{1}H NMR (400 MHz, $CDCl_3$) δ (ppm) = 7.81–7.77 (m, 4H, Ar-H), 7.58 (t, *J* = 7.4 Hz, 1H, Ar-H), 7.50 – 7.46 (m, 4H, Ar-H), 4.80 (s, 2H, <u>CH$_2$</u>-OH).
^{13}C NMR (101 MHz, $CDCl_3$) δ (ppm) = 196.67, 145.70, 137.74, 136.85, 132.58, 130.54 (2C), 130.15 (2C), 128.43 (2C), 126.54 (2C), 64.81.
HR-MS (ESI$^+$): Exact mass calculated for $C_{14}H_{12}NaO_2$ $[M+Na]^+$: 235.0730, found: 235.0736.

2.2.6 2,2,2-Trifluoro-*N*-(4-(hydroxymethyl)benzyl)acetamide (23) [6]

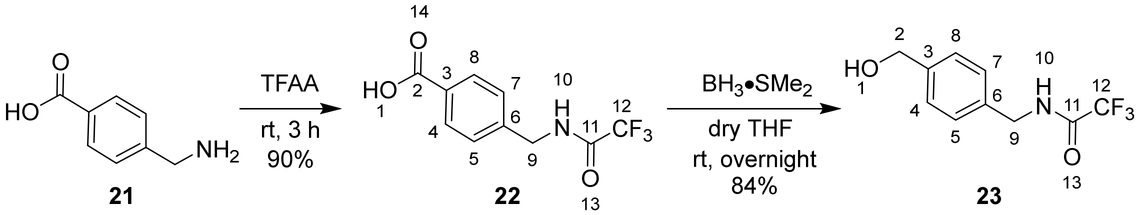

Trifluoroacetic anhydride (8.34 mL, 12.60 g, 60 mmol, 3.00 eq.) was added to a solution of aminomethylbenzoic acid **21** (3.00 g, 19.84 mmol, 1.00 eq.) at 0 °C, and the reaction mixture was stirred for 3 h at room temperature. After completion, the reaction was quenched with cold water (10 mL). The resulting precipitate was filtered, washed with cold water and dried under high vacuum to obtain the desired product **22** a white solid (4.40 g, 90%).
TLC ($MeOH/CH_2Cl_2$ = 6:94); R_f = 0.54.
^{1}H NMR (400 MHz, DMSO-*d*$_6$) δ (ppm) = 12.95 (s, 1H, OH), 10.08 (t, *J* = 6.0 Hz, NH), 7.93 (d, *J* = 8.2 Hz, 2H, Ar-H), 7.39 (d, *J* = 8.2 Hz, 2H, Ar-H), 4.46 (d, *J* = 6.0 Hz, 2H, <u>CH$_2$</u>-NH).
^{13}C NMR (101 MHz, DMSO-*d*$_6$) δ (ppm) = 167.09, 156.54 (q, *J* = 36.2 Hz, C-11), 142.47, 129.85, 129.61 (2C), 127.40 (2C), 116.01 (q, *J* = 286.3 Hz, CF_3), 42.36 (C-9).
^{19}F NMR (376 MHz MHz, DMSO-*d*$_6$) δ (ppm) = -74.86 (CF_3).
HR-MS (ESI$^-$): Exact mass calculated for $C_{10}H_7F_3NO_3$ $[M-H]^-$: 246.0384, found: 246.0379.

BH_3·SMe_2 (5 mL, 4.06 g, 53.4 mmol, 3.00 eq.) was added slowly to a solution of compound **22** (4.3 g, 17.4 mmol, 1.00 eq.) in dry THF (180 mL) under nitrogen atmosphere at 0 °C.

Afterwards, the reaction mixture was stirred overnight at room temperature. After completion, the reaction was quenched with methanol (35 mL) and further stirred for 1 h. Solvents were removed under reduced pressure and the residue was purified by column chromatography using 3% methanol in dichloromethane to obtain the desired product **23** as a white solid (3.5 g, 84%).

TLC (MeOH/CH_2Cl_2 = 6:94); R_f = 0.57.

^{1}H NMR (400 MHz, $CDCl_3$) δ (ppm) = 7.35 (d, *J* = 8.2 Hz, 2H, Ar-H), 7.27 (d, *J* = 8.2 Hz, 2H, Ar-H), 6.76 (s, 1H, NH), 4.68 (s, 2H, $\underline{CH_2}$-OH), 4.50 (d, *J* = 5.8 Hz, 2H, $\underline{CH_2}$-NH).

^{13}C NMR (101 MHz, $CDCl_3$) δ (ppm) = 157.32 (q, *J* = 37.19 Hz, C11), 141.14, 135.33, 128.31 (2C), 127.69 (2C), 115.98 (q, *J* = 287.82 Hz, CF_3), 64.75 (C-2), 43.59 (C-9).

^{19}F NMR (376 MHz MHz, $CDCl_3$) δ (ppm) = -75.81.

HR-MS (ESI$^-$): Exact mass calculated for $C_{10}H_9F_3NO_2$ $[M-H]^-$: 232.0591; found: 232.0592.

2.3 Synthesis of *O^6*-(4-aminomethylbenzyl)guanine = BG-NH_2(25)

The synthesis of compound **24** was slightly modified from literature known procedures.[6]

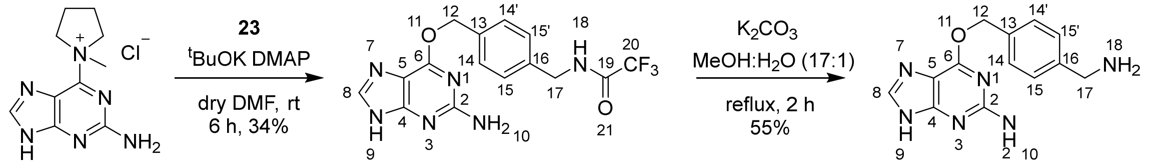

2 24 25

2,2,2-Trifluoro-*N*-(4-hydroxymethyl-benzyl)-acetamide **23** (560 mg, 2.4 mmol, 2 eq.) was dissolved in dry DMF (6 mL) under nitrogen atmosphere and potassium-*t*-butoxide (606 mg, 5.4 mmol, 4.5 eq.) was added. The reaction mixture was stirred for 5 min at room temperature. Further, 1-(2-amino-9H-purin-6-yl)-1-methylpyrrolidin-1-ium chloride **2** (300 mg, 1.2 mmol, 1.00 eq.) and 4-dimethylaminopyridine (22 mg, 0.18 0.25 eq.) were added and the solution was stirred for 6 h. Solvents were removed under reduced pressure and the crude product was purified by column chromatography using 5% MeOH in CH_2Cl_2 to obtain the desired product **24** as a white solid (150 mg, 34%).

TLC (MeOH/CH_2Cl_2 = 12:88); R_f = 0.40.

^{1}H NMR (400 MHz, DMSO-*d*$_6$) δ (ppm) = 7.83 (s, 1H, H-8), 7.49 (d, *J* = 7.9 Hz, 2H, Ar-H), 7.30 (d, *J* = 7.9 Hz, 2H, Ar-H), 6.27 (s, 2H, NH_2), 5.46 (s, 2H, CH_2-OH), 4.39 (s, 2H, CH_2-NH).

^{13}C NMR (101 MHz, DMSO-*d*$_6$) δ (ppm) = 159.61(2C, C-2, C-6), 156.38 (q, *J* = 36.7 Hz, C-19), 138.50 (C-8), 137.30, 135.94, 128.74 (2C), 127.49 (2C), 117.47, 66.43 (C-12) 42.40 (C-17).

HR-MS (ESI$^+$): Exact mass calculated for $C_{15}H_{13}F_3N_6NaO_2$ $[M+Na]^+$: 389.0944, found: 389.0931.

N-[4-(2-amino-*9H*-purin-6-yloxymethyl)-benzyl]-2,2,2-trifluoro-acetamide **24** (140 mg, 0.38 mmol, 1.00 eq.) was suspended in methanol (14 mL) and water (0.84 mL). After addition of K_2CO_3 (289 mg, 2.09 mmol, 5.2 eq.), the reaction mixture was refluxed for 2 h. The solvents were removed *in vacuo* and the product was purified by flash column chromatography using 10% MeOH in CH_2Cl_2 to obtain the desired product **25** as a white solid (57 mg, 55%).

TLC (MeOH/CH_2Cl_2 = 20:80, containing 2 drops of Et_3N); R_f = 0.14.

^{1}H NMR (400 MHz, DMSO-*d*$_6$): δ (ppm) = 7.83 (s, 1H, C-8), 7.46 (d, *J* = 8 Hz, 2H, Ar-H), 7.37 (d, *J* = 8 Hz, 2H, Ar-H), 6.28 (s, 2H, NH_2), 5.46 (s, 2H, $\underline{CH_2\text{-}O}$), 3.78 (s, 2H, $\underline{CH_2\text{-}NH}$).

^{13}C NMR (101 MHz, DMSO-*d*$_6$): δ (ppm) = 159.59 (C-6, C-2), 156.16 (C-4), 144.25, 138.63 (C-8), 134.57, 128.45 (2C), 127.04 (2C), 112.97 (C-5), 66.66 (C-12), 45.46 (C-17).

HR-MS (ESI$^+$): Exact mass calculated for $C_{13}H_{14}N_6NaO$ $[M+Na]^+$: 293.1121, found: 293.1128.

2.4 Synthesis of DBCO-NHS ester (33)

Synthesis of 4-(11,12-Didehydrodibenzo[*b,f*]azocin-5(6*H*)-yl)-4-oxobutanoic acid **32** (DBCO−COOH) according to reference [7]

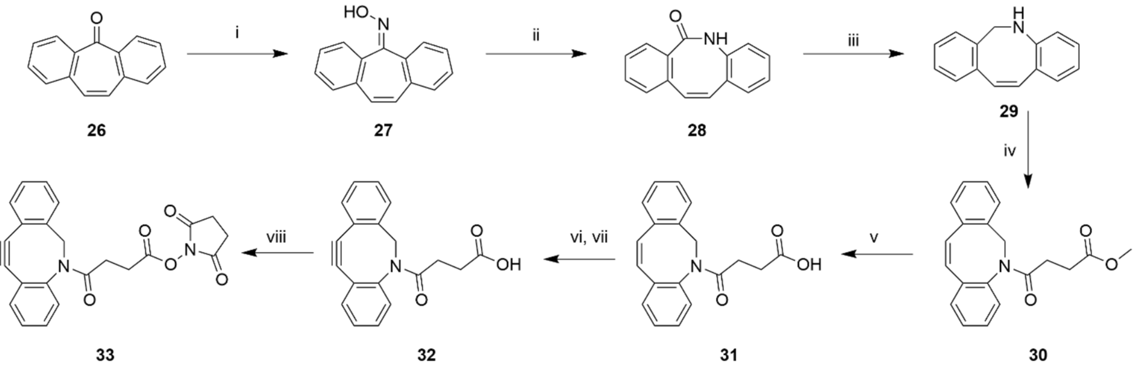

Scheme S1. i) Hydroxylamine hydrochloride, Na_2CO_3, EtOH, 90 °C, 24 h, 96%; ii) Eaton´s reagent, 100 °C, 45 min, 99%; ii) $LiAlH_4$, Et_2O, 49 °C, 21 h, 97%. iv) KOH (2 M), methyl 4-chloro-4-oxobutanoate, CH_2Cl_2, rt, 4 h, 86%; v) lithium hydroxide monohydrate, MeOH/H_2O (2:1), 80 °C, 1.5 h, 98%; vi) Br_2, CH_2Cl_2, rt, 1h; vii) KO^{t}Bu, THF, -78 °C, 2.5 h, 45%; viii) Et_3N, TSTU, THF, rt, 15 h, 82%

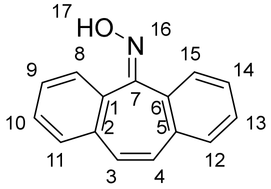

27

Hydroxylamine hydrochloride (2.70 g, 38.9 mmol, 4.01 eq.) and Na_2CO_3 (1.54 g, 14.6 mmol, 1.50 eq.) were added to a suspension of dibenzosuberenone **26** (2.00 g, 9.70 mmol, 1.00 eq.) in anhydrous EtOH (12 mL) and the mixture was stirred at 90 °C for 24 h. After cooling to room temperature, the suspension was poured into ice cold 1 M HCl (150 mL). The resulting off-white slurry was filtered, washed with 1 M HCl (3 x 10 mL) and distilled H_2O (3 x 10 mL) and dried under high vacuum to afford **27** as an off-white solid (2.07 g, 96%).

^{1}H NMR (400 MHz, DMSO-*d*$_6$) δ (ppm) = 9.85 (s, 1H, OH), 7.35 – 7.28 (m, 2H, H-8; H-15), 7.27 – 7.19 (m, 2H, C9-H; H-14), 7.18 – 7.07 (m, 4H, H-10; H-11; H-12; H-13), 7.00 (d, *J* = 11.6 Hz, 1H, Alkene-CH), 6.89 (d, *J* = 11.6 Hz, 1H, Alkene-CH).

^{13}C NMR (101 MHz, DMSO-*d*$_6$) δ (ppm) = 171.70 (C=N-OH), 136.31, 136.14, 134.47, 133.45, 132.65 (Alkene-C), 130.21 (Alkene-C), 128.98, 128.94, 128.08, 127.81, 127.77, 127.41, 126.41, 126.22.

HR-MS (ESI$^+$): Exact mass calculated for $C_{15}H_{11}NNaO$ $[M+Na]^+$: 244.07328, found: 244.07305.

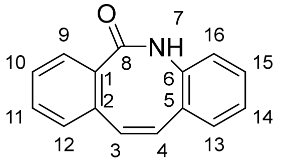

28

27 (1.98 g, 8.95 mmol, 1.00 eq.) was suspended in the Eaton's reagent (4 mL) and the orange mixture was stirred at 100 °C for 45 min. Afterwards, the hot solution was poured onto ice (10.5 g) and mixed with a glass spatula to quench Eaton´s reagent. The light brown suspension was then filtered and the filter cake was washed with distilled H_2O until the filtrate was neutral. After drying under high vacuum, **28** was obtained as a light grey-brown powder (1.95 g, 99%).
^{1}H NMR (400 MHz, DMSO-d_6) δ (ppm) = 9.85 (s, 1H, NH), 7.35 – 7.28 (m, 2H, H-9; H-16), 7.28 – 7.19 (m, 2H, H-10; H-15), 7.18 – 7.07 (m, 4H, H-11; H-12; H-13; H-14), 7.00 (d, *J* = 11.6 Hz, 1H, H-4), 6.89 (d, *J* = 11.6 Hz, 1H, H-3).
^{13}C NMR (101 MHz, DMSO-d_6) δ (ppm) = 171.70 (C=O), 136.31 (C-6), 136.14 (C-1), 134.47 (C-2), 133.45 (C-5), 132.65 (C-4), 130.21 (C-3), 128.99 (C-9), 128.94 (C-12), 128.08 (C-10), 127.81 (C-16), 127.77 (C-13), 127.42 (C-15), 126.41 (C-14), 126.22 (C-11).
HR-MS (ESI$^+$): Exact mass calculated for $C_{15}H_{11}NNaO$ $[M+Na]^+$:244.07328, found: 244.07381.

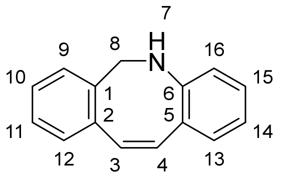

29

28 (1.90 g, 8.60 mmol, 1.00 eq.) was suspended in anhydrous Et_2O (15 mL) under nitrogen atmosphere and the mixture was cooled to 0 °C. Then $LiAlH_4$ (1.64 g, 43.3 mmol, 5.03 eq.) was added in portions upon stirring. After the mixture was allowed to come to rt, it was diluted with anhydrous Et_2O (5 mL) and stirred at 49 °C for 21 h. The green-grey suspension was cooled to 0 °C and diluted with CH_2Cl_2 (15 mL) before being quenched by the dropwise addition of distilled H_2O (2 mL). After stirring at 0 °C for 1.5 h, 4 M NaOH (2 mL) was added dropwise, followed by distilled H_2O (6 mL). The reaction mixture was allowed to come to rt and stirred for 45 min. During this time its color changed from olive-green to yellow. Then anhydrous Na_2SO_4 was added until the organic phase appeared clear. The mixture was filtered and the filter cake was washed with EtOAc. Removal of the solvent *in vacuo* afforded **29** as a yellow-brown solid (1.73 g, 97%).
^{1}H NMR (400 MHz, DMSO-d_6) δ (ppm) = 7.29 – 7.15 (m, 3H, H-10; H-11; H-12), 7.15 – 7.11 (m, 1H, H-9), 6.87 (dd, *J* = 7.8, 1.6 Hz, 1H, H-13), 6.80 (ddd, *J* = 8.5, 7.0, 1.6 Hz, 1H, H-15), 6.55 – 6.38 (m, 3H, H-4; H-14; H-16), 6.25 (d, *J* = 13.1 Hz, 1H, H-3), 4.46 (d, *J* = 7.2 Hz, 2H, H-8).
^{13}C NMR (101 MHz, DMSO-d_6) δ (ppm) = 148.26 (C-6), 139.13 (C-2), 138.52 (C-1), 134.54 (C-13), 133.11 (C-14), 129.66 (C-11), 129.35 (C-9), 127.83 (C-15), 127.41 (C-10), 127.18 (C-12), 126.35 (C-3), 120.16 (C-5), 117.26 (C-16), 115.93 (C-14), 47.96 (C-8)
HR-MS (ESI$^+$): Exact mass calculated for $C_{15}H_{14}N$ $[M+H]^+$: 208.11208, found: 208.11200.

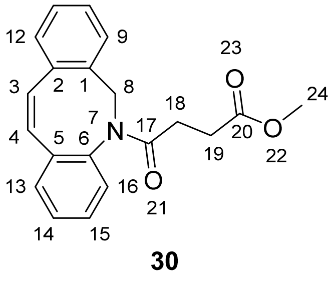

30

A solution of **29** (1.71 g, 8.27 mmol, 1.00 eq.) in CH_2Cl_2 (15 mL) was cooled to 0 °C and 2 M KOH (13.5 mL) was added upon stirring. To this mixture, a solution of methyl 4-chloro-4-oxobutanoate (1.40 mL, 11.4 mmol, 1.38 eq.) in CH_2Cl_2 (2.6 mL) was added dropwise over 25 min at 0 °C. The emulsion was allowed to come to rt and stirred for 4 h. Phases were separated and the aqueous phase was washed with CH_2Cl_2 (4 x 5 mL). The combined organic phases were dried over Na_2SO_4 and concentrated onto celite. Column chromatography was performed twice (first column: SiO_2, *n*-hexane/EtOAc 5:1 then 4:1 then 3:2; second column: SiO_2, *n*-hexane/EtOAc 4:1 then 3:1 then 2:1) and **30** was obtained as a beige solid (2.28 g, 86%).

^{1}H NMR (400 MHz, DMSO-d_6) δ (ppm) = 7.42 – 7.32 (m, 4H, H-13; H-14; H-15; H-16), 7.27 (dd, *J* = 7.7, 1.9 Hz, 1H, H-9), 7.22 – 7.11 (m, 3H, H-10; H-11; H-12), 6.80 (d, *J* = 12.9 Hz, 1H, H-3), 6.68 (d, *J* = 13.0 Hz, 1H, H-4), 5.40 (d, *J* = 15.4 Hz, 1H, H_a-8), 4.23 (d, *J* = 15.4 Hz, 1H, H_b-8), 3.50 (s, 3H, CH_3), 2.48 – 2.31 (m, 2H, H_{ab}-18), 2.26 (ddd, *J* = 16.7, 7.9, 5.8 Hz, 1H, H_a-19), 1.87 (dt, *J* = 16.7, 6.3 Hz, 1H, H_b-19).

^{13}C NMR (101 MHz, DMSO-d_6) δ (ppm) = 172.63 (C-20), 169.84 (C-17), 140.33 (C-6), 136.21 (C-5), 135.35 (C-2), 134.68 (C-1), 131.94 (C-3), 131.87 (C-12), 130.57 (C-13), 129.98 (C-9), 128.84 (Ar-C), 128.42 (Ar-C), 128.12 (Ar-C), 127.28 (C-11), 127.19 (C4), 126.82 (C-10), 53.48(CH_2-N), 51.26 (C-24), 29.11 (C-19), 28.65 (C-18).

HR-MS (ESI$^+$): Exact mass calculated for $C_{20}H_{19}NNaO_3$ $[M+Na]^+$: 344.12571, found: 344.12581.

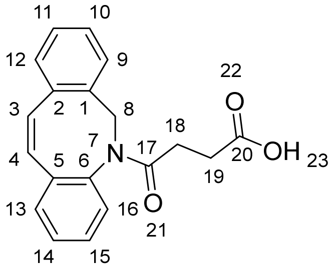

31

30 (2.25 g, 7.01 mmol, 1.00 eq.) was suspended in a 2:1 mixture of MeOH and distilled H_2O (6.8 mL). $LiOH \cdot H_2O$ (1.76 g, 42.0 mmol, 5.99 eq.) was added and the mixture was stirred at 80 °C for 1.5 h under nitrogen atmosphere. The suspension was allowed to come to rt and 1 M HCl (40.5 mL) was added. The mixture was extracted with CH_2Cl_2 (1 x 35 mL then 3 x 25 mL) and the combined organic phases were dried over $MgSO_4$. Removal of the solvent in vacuo afforded **31** as an off-white foam (2.11 g, 98%).

^{1}H NMR (400 MHz, DMSO-d_6) δ (ppm) = 12.02 (s, 1H, OH), 7.44 – 7.31 (m, 4H, H-13; H-14; H-15; H-16), 7.27 (dd, *J* = 7.7, 2.2 Hz, 1H, H-9), 7.21 – 7.11 (m, 3H, H-10; H-11; H-12), 6.79 (d, *J* = 12.9 Hz, 1H, H-3), 6.67 (d, *J* = 12.9 Hz, 1H, H-4), 5.41 (d, *J* = 15.4 Hz, 1H, H_a-8), 4.23 (d, *J* = 15.4 Hz, 1H, H_b-8), 2.42 – 2.16 (m, 3H, H_{ab}-19; H_a-18), 1.82 (dt, *J* = 16.4, 6.7 Hz, 1H, H_b-18).

^{13}C NMR (101 MHz, DMSO-d_6) δ (ppm) = 173.66 (C-20), 170.04 (C-17), 140.41 (C-6), 136.26 (C-5), 135.34 (C-2), 134.74 (C-1), 131.94 (C-3), 131.86 (C-12), 130.48 (C-13), 129.94 (C-9), 128.82 (Ar-C), 128.45 (Ar-C), 128.09 (Ar-C), 127.32 (C-11), 127.20 (C-4), 126.81 (C-10), 53.47 (CH_2-N), 29.17 (C18), 28.92 (C19).

HR-MS (ESI$^+$): Exact mass calculated for $C_{19}H_{18}NO_3$ $[M+H]^+$: 308.12812, found: 308.12507.

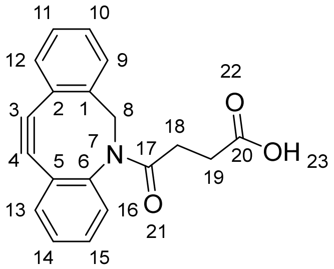

32

A solution of Br_2 (300 µL, 5.86 mmol, 1.72 eq.) in CH_2Cl_2 (0.7 mL) was added dropwise to a solution of **31** (1.05 g, 3.40 mmol, 1.00 eq.) in CH_2Cl_2 (14.7 ml) at 0 °C. After the solution was stirred at rt for 1 h, saturated aqueous $Na_2S_2O_3$ solution (10 mL) was added and the mixture was stirred at rt for 5 min. Phases were separated and the organic phase was washed with distilled H_2O (3 x 10 mL), 1 M HCl (1 x 10 mL) and brine (1 x 10 mL) and dried over $MgSO_4$. Removal of the solvent *in vacuo* gave the brominated intermediate as a white-green foam. Since this compound is unstable, it should be eliminated on the same day. The brominated intermediate was dissolved in anhydrous THF (6.75 mL) and cooled to -78 °C. Separately, KO^tBu (1.63 g, 14.6 mmol, 4.28 eq.) was suspended in anhydrous THF (14 mL) under nitrogen atmosphere and cooled to -78 °C before being cannulated into the cooled solution of the brominated intermediate. After the mixture was stirred at -78 °C for 2.5 h, Et_2O (20 mL) was added and the mixture was allowed to come to rt. The suspension was washed with 1 M HCl (3 x 20 mL) and brine (1 x 10 mL) and dried over $MgSO_4$. The solvent was partially removed at rt under reduced pressure until crystal formation could be observed. Et_2O (50 mL) was added and the mixture was stored at -20 °C overnight. The precipitate was filtered, washed with small amounts of ice-cold Et_2O and dried under high vacuum to give **32** as a white-brown powder (463 mg, 45%).

^{1}H NMR (400 MHz, DMSO-d_6) δ (ppm) = 11.99 (s, 1H, OH), 7.68 – 7.64 (m, 1H, H-16), 7.62 (dd, J = 7.3, 1.6 Hz, 1H, H-9), 7.54 – 7.43 (m, 3H, H-13; H-14; H-15), 7.41 – 7.27 (m, 3H, H-10; H-11; H-12), 5.03 (d, J = 14.1 Hz, 1H, H_a-8), 3.62 (d, J = 14.1 Hz, 1H, H_b-8), 2.59 (ddd, J = 16.7, 7.6, 6.5 Hz, 1H, H_a-18), 2.30 (ddd, J = 17.1, 7.5, 6.3 Hz, 1H, H_a-19), 2.18 (dt, J = 17.2, 6.6 Hz, 1H, H_b-19), 1.77 (dt, J = 16.6, 6.4 Hz, 1H, H_b-18).

^{13}C NMR (101 MHz, DMSO-d_6) δ (ppm) = 173.59 (C-20), 170.75 (C-17), 151.47 (C-6), 148.43 (C-1), 132.41 (C-9), 129.64 (C-16), 128.95 (C-15), 128.22 (C-14), 128.04 (C-10), 127.70 (C-11), 126.84 (C-13), 125.18 (C-12), 122.55 (C-2), 121.56 (C-5), 114.29 (C-3), 108.04 (C-4), 54.96 (C-8), 29.27 (C-18), 28.95 (C-19).

HR-MS (ESI$^+$): Exact mass calculated for $C_{19}H_{16}NO_3$ $[M+H]^+$: 306.11247, found: 306.11245.

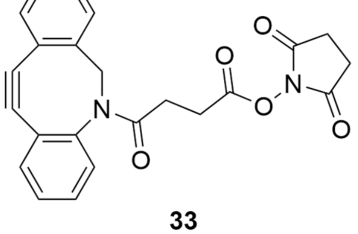

33

32 (782 mg, 2.56 mmol, 1.00 eq.) was dissolved in anhydrous THF (35 mL) under nitrogen atmosphere. Et_3N (1.10 mL, 7.89 mmol, 3.08 eq.) and TSTU (1.55 g, 5.15 mmol, 2.01 eq.) were added and the suspension was stirred at rt for 15 h. After the solvent was removed *in vacuo* the residue was suspended in EtOAc (120 mL), washed with distilled H_2O (3 x 50 mL)

and dried over Na_2SO_4. The solvent was removed under reduced pressure and the residue was dissolved in CH_2Cl_2 (30 mL) before *n*-hexane (100 mL) was added. The solution was carefully concentrated under reduced pressure until a slightly yellow solid started to precipitate. The suspension was stored at -20 °C for 15 min before the precipitate was filtered, washed with small amounts of ice-cold *n*-hexane and dried under high vacuum. The filtrate was subjected to two more rounds of precipitation as described above. After the last round, the filtrate was concentrated onto celite and a column chromatography (SiO_2, *n*-hexane/EtOAc 2:1 then 1:1) was performed. **33** was obtained as a slightly yellow powder (843 mg, 82%).
^{1}H NMR (400 MHz, DMSO-d_6) δ (ppm) = 7.64 (ddd, *J* = 7.7, 6.4, 1.8 Hz, 2H, H-9; H-16), 7.55 – 7.45 (m, 3H, H-13; H-14; H-15), 7.42 – 7.27 (m, 3H, H-10; H-11; H-12), 5.05 (d, *J* = 14.1 Hz, 1H, H_a-8), 3.66 (d, *J* = 14.0 Hz, 1H, H_b-8), 2.82 – 2.70 (m, 6H, H_a-18, H_a-19, H_{ab}-25, H_{ab}-26), 2.69 – 2.58 (m, 1H, H_b-18), 1.95 – 1.83 (m, 1H, H_b-19).
^{13}C NMR (101 MHz, DMSO-d_6) δ (ppm) = 170.06 (2C, C-24, C-27), 169.59 (C-17), 168.52 (C-20), 151.12 (C-6), 148.25 (C-1), 132.43 (C-9), 129.57 (C-16), 129.06 (C-15), 128.43 (C-14), 128.13 (C-10), 127.78 (C-11), 126.94 (C-13), 125.24 (C-12), 122.49 (C-2), 121.66 (C-5), 114.34 (C-3), 107.88 (C-4), 55.06 (C-8), 28.58 (C-19), 25.89 (C-18), 25.41 (2C, C-25, C-26).

2.5. Synthesis of 6-azido-1H,3H-benzo[de]isochromene-1,3-dione (35)[8]

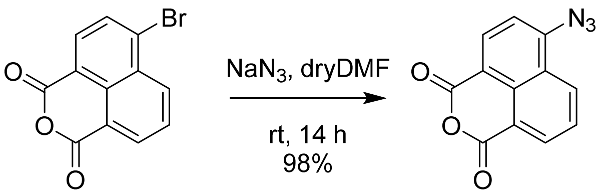

34 **35**

To a solution of 4-bromo-1,8-naphthalic anhydride **34** (1.00 g, 3.60 mmol, 1.00 eq.) in dry DMF, sodium azide (351 mg, 5.40 mmol, 1.50 eq.) was added and the mixture was stirred at room temperature for 14 h. After completion, water (60 mL) was added and the precipitate was filtered and washed with water. The precipitate was dried under high vacuum to get a yellow solid (850 mg, 98%).
TLC (EtOAc / *n*-hexane = 40:60); R_f = 0.70.
^{1}H NMR (400 MHz, DMSO-d_6) δ (ppm) = 8.53 (d, *J* = 7.2 Hz, 1H), 8.46 (dd, *J* = 8.2, 4.8 Hz, 2H), 7.87 (t, *J* = 7.9 Hz, 1H), 7.76 (d, *J* = 8.0 Hz, 1H).
^{13}C NMR (101 MHz, DMSO-d_6) δ (ppm) = 160.67, 160.08, 144.11, 133.31, 133.27, 130.70, 129.47, 127.63, 123.54, 119.16, 116.33, 114.68.
HR-MS (ESI$^+$): Exact mass calculated for $C_{12}H_5N_3NaO_3$ $[M+Na]^+$: 262.0223, found: 262.0225.

2.6 General procedure for synthesis of *O*^{6}-alkyl guanine cofactors (3–6, 8, 9) (Table S1)

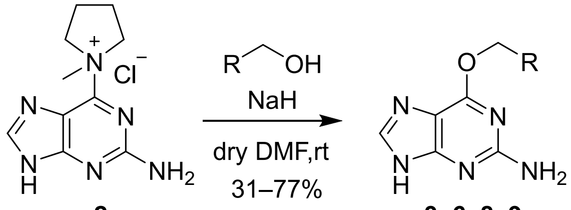

2 **3–6, 8, 9**

The respective alcohol (1.10–4.00 eq.) was dissolved in dry DMF (3–4 mL). Sodium hydride (60% dispersion in mineral oil) (4 eq.) was added in portions to the above solution over 5 min. Afterwards, the reaction mixture was stirred for 10-15 min. 1-(2-Amino-9H-purin-6-yl)-

1-methylpyrrolidin-1-ium chloride (**2**, 1.00 eq.) and 4-dimethylaminopyridine (0.25 eq.) were added. Further the reaction mixture was stirred at room temperature. Distilled water was added to quench the excess sodium hydride. Solvents were removed under reduced pressure and the crude mixture was purified by silica gel column chromatography using gradient based on MeOH:CH_2Cl_2 (4-6 % MeOH) to afford the desired alkyl guanine cofactors (**3**–**6, 8, 9**) as a pure white solid (For further details see Table S1).

2.7 Synthesis of *N*-(4-(((2-amino-9*H*-purin-6-yl)oxy)methyl)benzyl)-4-(11,12-didehydrodibenzo[b,*f*]azocin-5(6*H*)-yl)-4-oxobutanamide (7)[9]

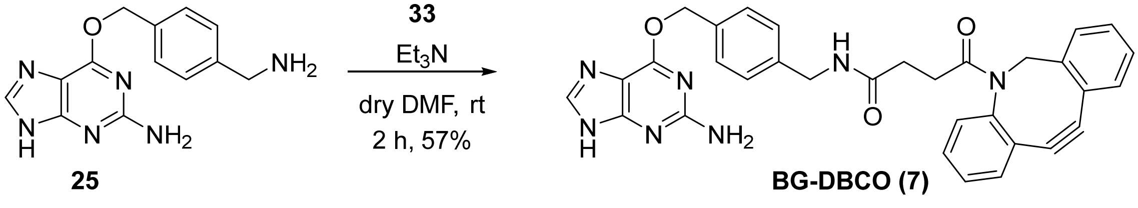

25 **BG-DBCO (7)**

*O*6-(4-aminomethylbenzyl)guanine **25** (15.7 mg, 39.0 µmol, 1.00 eq.) was dissolved in anhydrous DMF (0.6 mL) under nitrogen atmosphere. Et_3N (300 µL, 2.15 mmol, 55.20 eq.) was added and the solution was stirred at rt for 10 min. Compound **33** (15.2 mg, 56.2 µmol, 1.44 eq.) was added and the yellow solution was stirred at rt for 2 h before distilled H_2O (8 mL) was added. The mixture was extracted with EtOAc (1 x 6 mL; then 3 x 3 mL), the combined organic phases were washed with distilled H_2O (10 x 5 mL) and dried over Na_2SO_4. The solvent was removed *in vacuo* and subsequent column chromatography (SiO_2, CH_2Cl_2 /MeOH 9:1) gave **7** as a slightly rose solid (12.4 mg, 57%).

2.8 Synthesis of 2-(4-(((2-amino-9H-purin-6-yl)oxy)methyl)benzyl)-6-azido-1H-benzo [de]isoquinoline-1,3(2H)-dione (10)[10]

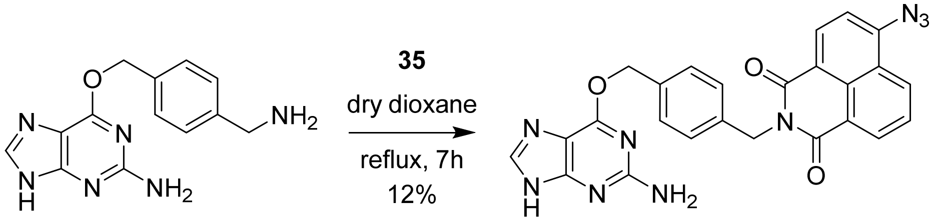

25 **BG-AzNP (10)**

*O*6-(4-aminomethylbenzyl)guanine **25** (50 mg, 0.19 mmol, 1.10 eq.) and 6-azido-1H,3H-benzo[de]isochromene-1,3-dione **35** (41 mg, 0.17 mmol, 1.00 eq.) were suspended in dry dioxane (3 mL). The resulting suspension was refluxed for 7 h in the dark at 100 °C. The reaction mixture was then allowed to cool to room temperature. Dioxane was removed under reduced pressure. The crude residue was purified by column chromatography with a gradient of 4% MeOH in CH_2Cl_2 to obtain the desired product **10** as a yellow solid (10 mg, 12%).

2.9 Spectral data for *O*6-alkyl guanine cofactors (3–10)

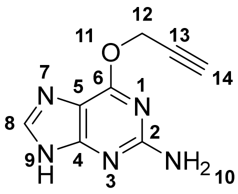

prop6G (3)

TLC (MeOH/CH_2Cl_2 = 10:90); R_f = 0.40.
^{1}H NMR (400 MHz, DMSO-d_6) δ (ppm) = 12.49 (s, 1H, NH), 7.85 (s, 1H, H-8), 6.33 (s, 2H, NH_2), 5.10 (d, J = 2.4 Hz, 2H, OCH_2), 3.56 (t, J = 2.4 Hz, 1H, H-14).
^{13}C NMR (101 MHz, DMSO-d_6) δ (ppm) = 159.56 (C-6), 158.86 (C-2), 155.43 (C-4), 138.27 (C-8), 113.41 (C-5), 79.34 (C-13), 77.77 (C-14), 52.78 (C-12).
HR-MS (ESI$^+$): Exact mass calculated for $C_8H_8N_5O$ $[M+H]^+$: 190.0723, found: 190.0731.

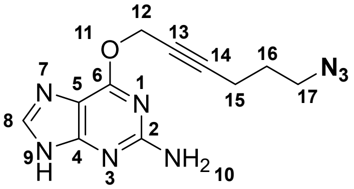

azihex$_6$G (4)

TLC (MeOH/CH_2Cl_2 = 10:90); R_f = 0.46.
^{1}H-NMR (400 MHz, DMSO-d_6) δ (ppm) = 12.52 (s, 1H, N-9), 7.85 (s, 1H, H-8), 6.30 (s, 2H, N-10), 5.09 (t, J = 2.2 Hz, 2H, H-12), 3.41 (t, J = 6.7 Hz, 2H, H-17), 2.33 (tt, J = 7.1, 2.2 Hz, 2H, H-15), 1.70 (p, J = 6.9 Hz, 2H, H-16).
^{13}C-NMR (101 MHz, DMSO-d_6) δ (ppm) = 158.73 (C-2, C-6), 155.87 (C-4), 138.60 (C-8), 113.05 (C-5), 85.83 (C-14), 76.36 (C-13), 53.24 (C-12), 49.57 (C-17), 27.24 (C-16), 15.45 (C-15).
HR-MS (ESI$^-$): Exact mass calculated for $C_{11}H_{11}N_8O$ $[M-H]^-$: 271.10613, found: 271.10468.

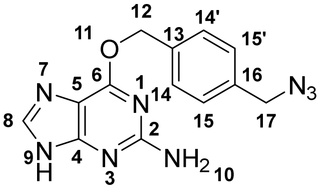

BG-CH$_2$N$_3$ (5)

TLC (MeOH/CH_2Cl_2 = 10:90); R_f = 0.50.
^{1}H NMR (400 MHz, DMSO-d_6) δ (ppm) = 12.47 (br.s, 1H, H-9), 7.83 (s, 1H, H-8), 7.54 (d, J = 8.1 Hz, 2H, H-14,14’), 7.39 (d, J = 8.1 Hz, 2H, H-15,15’), 6.30 (s, 2H, NH_2), 5.49 (s, 2H, OCH_2-12), 4.45 (s, 2H, H-17).
^{13}C NMR (101 MHz, DMSO-d_6) δ (ppm) = 159.70 (2C, C-2; C-6), 155.28 (C4), 138.03 (C-8), 136.80 (C-13), 135.42 (C-16), 128.84 (2C, C-14, C-14'), 128.59 (2C, C-15, C-15'), 66.41 (C-12), 53.35 (C-17).
HR-MS (ESI$^+$): Exact mass calculated for $C_{13}H_{13}N_8O$ $[M+H]^+$: 297.12068, found: 297.12201.

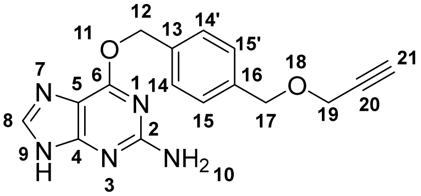

BG-prop (6)

TLC (MeOH/CH_2Cl_2 = 10:90); R_f = 0.44.
^{1}H NMR (400 MHz, DMSO-d_6) δ (ppm) = 12.46 (s, 1H, H-9), 7.84 (s, 1H, H-8), 7.49 (d, J = 8.1 Hz, 2H, H-14,14’), 7.35 (d, J = 8.1 Hz, 2H, H-15,15’), 6.29 (s, 2H, H-10), 5.48 (s, 2H, H-12), 4.53 (s, 2H, H-17), 4.18 (d, J = 2.4 Hz, 2H, H-19), 3.49 (t, 1H, J = 2.4 Hz, H-21).

^{13}C NMR (101 MHz, DMSO-d_6) δ (ppm) = 159.75 (C-6), 159.56 (C-2) 155.07 (C-4), 137.77 (C-8), 137.30 (C-16), 136.33 (C-13), 128.37 (2C, C-14, C-14'), 127.72 (2C, C-15, C-15'), 113.58 (C-5), 80.10 (C-20), 77.34 (C-21), 70.35 (C-17), 66.36 (C-12), 56.99 (C-19).
HR-MS (ESI$^+$): Exact mass calculated for $C_{16}H_{15}N_5NaO_2$ $[M+Na]^+$: 332.11180, found: 332.11103.

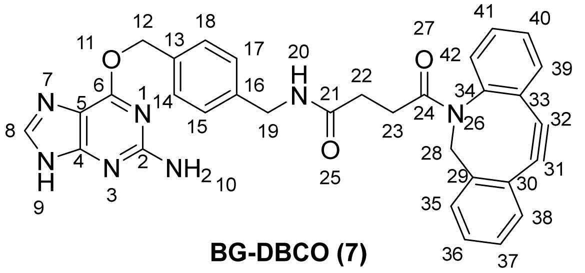

BG-DBCO (7)

TLC (MeOH/CH_2Cl_2 = 10:90); R_f = 0.43.
^{1}H NMR (400 MHz, DMSO-d_6) δ (ppm) = 12.42 (s, 1H, NH), 8.23 (t, J = 5.9 Hz, 1H, NH-20), 7.81 (s, 1H, H-8), 7.71 – 7.67 (m, 1H, H-42), 7.63 (dd, J = 7.3, 1.5 Hz, 1H, H-35), 7.54 – 7.43 (m, 3H, H-39, H-40, H-41), 7.42 – 7.26 (m, 5H, H-14, H-18, H-36, H-37,H-38), 7.18 (d, J = 8.1 Hz, 2H, H-15, H-17), 6.30 (s, 2H, NH_2), 5.44 (s, 2H, O-CH_2), 5.03 (d, J = 14.1 Hz, 1H, H_a-28), 4.16 (d, J = 5.9 Hz, 2H, H_{ab}-19), 3.61 (d, J = 14.0 Hz, 1H, H_b-28), 2.63 (dt, J = 15.9, 7.7 Hz, 1H, H_a-23), 2.30 (dt, J = 15.3, 7.5 Hz, 1H, H_a-22), 2.07 (ddd, J = 15.4, 7.8, 5.8 Hz, 1H, H_b-22), 1.80 (ddd, J = 16.4, 7.7, 5.8 Hz, 1H, H_b-23).
^{13}C NMR (101 MHz, DMSO-d_6) δ (ppm) = 171.09 (C-24), 171.06 (C-21), 159.86 (C-6), 159.67 (C-2), 155.21 (C-4), 151.61 (C-34), 148.47 (C-29), 139.34 (C-16), 137.81 (C-8), 135.19 (C-13), 132.45 (C-35), 129.67 (C42), 128.94 (C41), 128.43 (2C, C14, C-18), 128.16 (C-40), 128.04 (C-36), 127.70 (C-37), 127.20 (2C, C-15, C-17), 126.81 (C-39), 125.18 (C-38), 122.55 (C-30), 121.44 (C-33), 114.23 (C-31), 113.52 (C-5), 108.17 (C-32), 66.46 (C-12), 54.93 (C-28), 41.79 (C-19), 30.35 (C-22), 29.70 (C-23).
HR-MS (ESI$^+$): Exact mass calculated for $C_{32}H_{27}N_7NaO_3$ $[M+Na]^+$: 580.20676, found: 580.20669.

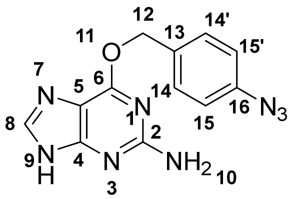

BG-N_3 (8)

TLC (MeOH/CH_2Cl_2 = 10:90); R_f = 0.53.
^{1}H NMR (400 MHz, DMSO-d_6) δ (ppm) = 12.44 (s, 1H, H-9), 7.81 (s, 1H, H-8), 7.55 (d, J = 8.0 Hz, 2H, H-14,H-14'), 7.14 (d, J = 8.0 Hz, 2H, H-15,H-15'), 6.31 (s, 2H, H-10), 5.46 (s, 2H, H-12).
^{13}C NMR (101 MHz, DMSO-d_6) δ (ppm) = 159.79 (C-6), 159.66 (C-2), 155.25 (C-4), 139.13 (C-16), 137.88 (C-8), 133.74 (C-13), 130.41 (2C, C-14, C-14'), 119.11 (2C, C-15, C-15'), 113.49 (C-5), 66.14 (C-12).
HR-MS (ESI$^+$): Exact mass calculated for $C_{12}H_{11}N_8O$ $[M+H]^+$: 283.10558, found: 283.10624.

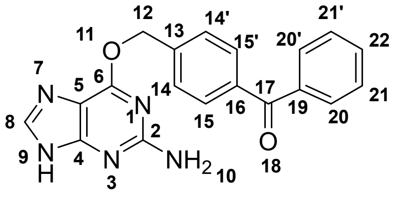

BG-Bz (9)

TLC ($MeOH/CH_2Cl_2$ = 10:90); R_f = 0.38.

^{1}H NMR (400 MHz, DMSO-d_6) δ (ppm) = 12.49 (s, 1H, H-9), 7.86 (s, 1H, H-8), 7.78–7.73 (m, 4H, H-15, 15',20, 20'), 7.70 –7.66 (m, 3H, H-14, H-14', H-22), 7.58–7.54 (m, 2H, H-21, H-21'), 6.32 (s, 2H, H-10), 5.61 (s, 2H, H-12).

^{13}C NMR (101 MHz, DMSO-d_6) δ (ppm) = 195.51 (C-17), 159.67 (2C, C-2,C-6), 155.37 (C-4), 141.72 (C-13), 138.01 (C-8), 137.03 (C-19), 136.51 (C-16), 132.76 (C-22), 129.86 (2C, C-15,C-15'), 129.67 (2C, C-20, C-20'), 128.63 (2C, C-21,C-21'), 128.11 (2C, C-14,C-14'), 113.46 (C-5), 66.02 (C-12).

HR-MS (ESI$^+$): Exact mass calculated for $C_{19}H_{16}N_5O_2$ $[M+H]^+$: 346.12985, found: 346.13120.

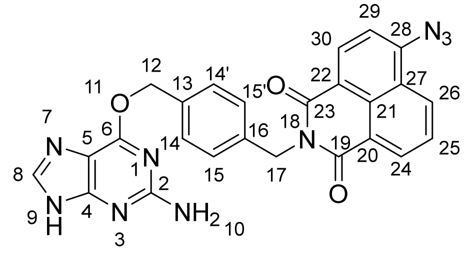

BG-AzNP (10)

TLC ($MeOH/CH_2Cl_2$ = 6:94); R_f = 0.23.

^{1}H NMR (600 MHz, DMSO-d_6): δ (ppm) =12.39 (s, 1H), 8.58 – 8.53 (m, 1H), 8.54 – 8.48 (m, 1H), 8.48 – 8.42 (m, 1H), 7.91 – 7.84 (m, 1H), 7.78 (s, 2H), 7.44 (d, *J* = 8.2 Hz, 2H), 7.38 (d, *J* = 8.4 Hz, 2H), 6.27 (s, 2H), 5.42 (s, 2H), 5.25 (s, 2H).

^{13}C NMR (150 MHz, DMSO-d_6) δ (ppm) = 163.37 (C- 23), 162.91, 159.82 (C-6), 159.63 (C-2), 156.17, 155.20 (C-4), 143.18, 137.79, 137.18, 135.66, 131.96 (C-26), 131.89 (C-24), 128.67, 128.63 (2C, C-14, C-14'), 128.50, 127.67 (2C, C-15, C-15'), 127.43, 123.64, 122.10, 118.07, 116.12, 113.48 (C-5), 66.43 (C-12), 42.75 (C-17).

HR-MS (ESI$^+$): Exact mass calculated for $C_{25}H_{18}N_9O_3$ $[M+H]^+$: 492.15271, found: 492.15411.

2.10 Synthesis of N^1 and N^6 modified adenosine standards

2.10.1 N^1-benzophenone modified adenosine (BnBz^{1}A)[11]

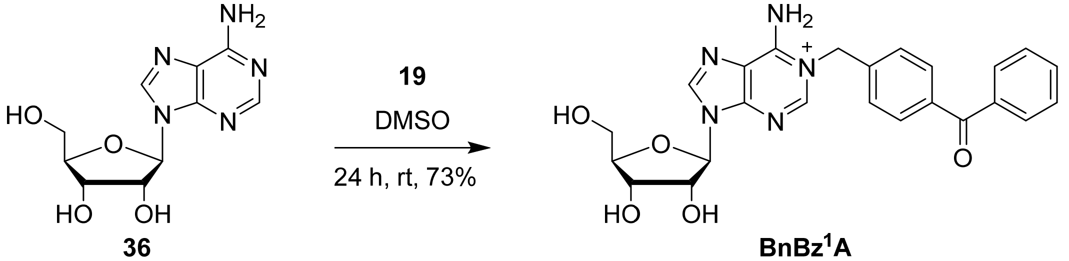

36 **BnBz^{1}A**

Adenosine **36** (100 mg, 0.37 mmol, 1.00 eq.) and 4-bromomethyl benzophenone **19** (124 mg, 0.45 mmol, 1.00 eq.) were suspended in DMSO (2 mL). After vigorously stirring at room

temperature for 24 h, the reaction mixture was lyophilized. The residue was dissolved in mixture of H_2O, acetonitrile and methanol. The product was purified by preparative RP-HPLC using aqueous mobile phase A (10 mM NH_4OAc, pH 6.1) and organic mobile phase B (100% acetonitrile). The collected product fractions were lyophilized and the desired product $(BnBz)^1A$ (126 mg , 73%) was obtained as white solid. The analytical data was in agreement with previous reports.[11]

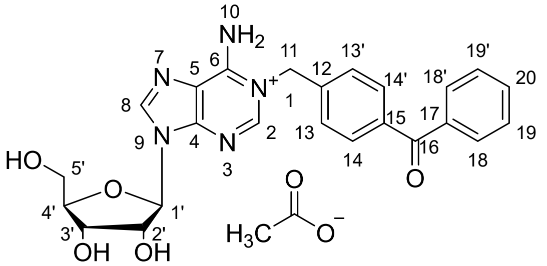

^{1}H NMR (400 MHz, DMSO-*d*$_6$): δ (ppm) = 8.32 (s, 1H, H-8), 8.19 (s, 1H, H-2), 7.76 – 7.68 (m, 4H, H-18, H-18', H-19, H-19'), 7.71 – 7.62 (m, 1H, H-19), 7.56 – 7.50 (m, 4H, H-13, H-13', H-14, H-14'), 5.78 (d, *J* = 5.9 Hz, 1H, H-1'), 5.35 (s, 2H, H-11), 4.48 (t, *J* = 5.5 Hz, 1H, H-2'), 4.11 (dd, *J* = 5.3, 3.1 Hz, 1H, H-3'), 3.93 (, q, *J* = 3.7 Hz, 1H, H-4'), 3.64 (dd, *J* = 12.0, 4.0, 1H, H-5'a), 3.54 (dd, *J* = 12.0, 4.0 Hz, 1H, 5'b), 1.88 (s, 4H, acetate).
^{13}C NMR (101 MHz, DMSO-*d*$_6$) δ (ppm) = 195.40 (C-16), 153.52 (C-6), 148.59 (C-2), 142.32 (C-17), 138.12 (C-8), 137.00 (C-15), 136.01 (C-12), 132.73 (C-20), 129.92 (2C, C-19, C-19'), 129.65(2C, C-18, C-18'), 128.60 (2C, C-14, C-14'), 127.51 (2C,C-13, C-13'), 122.96 (C-5), 87.55 (C-1'), 85.74 (C-4'), 74.00 (C-2'), 70.42 (C-3'), 61.44 (C-5'), 48.84 (C-11), 21.14 (acetate CH_3).
HR-MS (ESI$^+$): Exact mass calculated for $C_{24}H_{23}N_5NaO_5$ $[M+Na]^+$: 484.15914, found: 484.15847.

2.10.2 4-Aminomethyl benzophenone (38) [12]

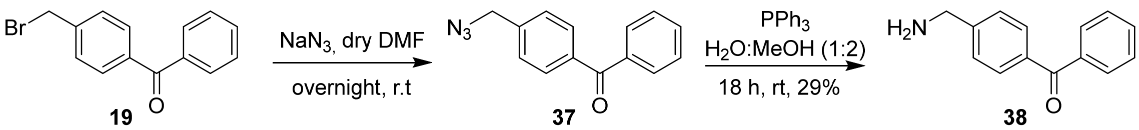

A solution of 4-bromo methyl benzophenone **19** (500 mg, 1.82 mmol, 1.00 eq.) and NaN_3 (118 mg, 2.73 mmol, 1.50 eq.) in dry DMF (3 mL) was stirred at room temperature overnight and then diluted with water (3 mL) followed by extraction with diethyl ether (3 x 3 mL). The organic layer was dried over anhydrous Na_2SO_4 and concentrated *in vacuo* to obtain **37** as a yellow oil. **TLC** (EtOAc/*n*-hexane = 10:90); R_f = 0.40.
The azido intermediate **37** was dissolved in a mixture of H_2O (2 mL) and MeOH (4 mL), then triphenylphosphine (716 mg, 2.73 mmol, 1.50 eq.) was added and the resulting mixture was stirred at room temperature for 18 h. After diluting with H_2O (2 mL), the mixture was acidified with 1N HCl and extracted with ethyl acetate. The combined organic layers were dried over anhydrous Na_2SO_4 and the solvent was removed *in vacuo* to furnish **38** as a yellow oil (111 mg, 29%). Analytical data in agreement with previous report.[12]
TLC (MeOH/CH_2Cl_2 =20:80); R_f = 0.40.
^{1}H NMR (400 MHz, $CDCl_3$) δ (ppm) = 7.81 – 7.78 (m, 4H, Ar-H), 7.59 (m, 1H, Ar-H); 7.50 – 7.43 (m, 4H, Ar-H); 3.98 (s, 2H, $\underline{CH_2}$-N); 1.61 (br.s, 2H, NH_2).
^{13}C NMR (101 MHz, $CDCl_3$) δ (ppm) = 196.57, 161.78, 147.43, 137.79, 136.33, 132.48, 130.61 (2C), 130.09 (2C), 128.38 (2C), 127.11 (2C), 46.09.

HR-MS (ESI⁺): Exact mass calculated for $C_{14}H_{14}NO$ $[M+H]^+$: 212.10699, found: 212.10753.

2.10.3 N⁶-benzophenone modified adenosine (BnBz⁶A)

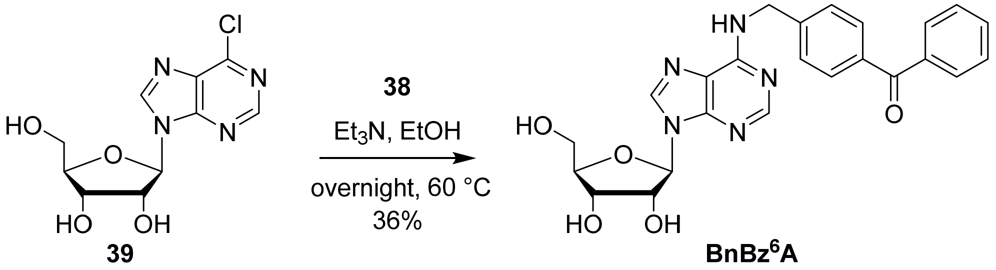

6-Chloropurine nucleoside **39** (70 mg, 0.24 mmol, 1.00 eq.) and 4-aminomethyl benzophenone **38** (103 mg, 0.48 mmol, 2.00 eq.) were suspended in ethanol (6 mL) and Et_3N (132 µL, 0.96 mmol, 4.00 eq.) was added to the suspension. The mixture was stirred overnight at 60 °C. Then solvents were evaporated and the residue was further dissolved in 3 ml of EtOH (3 mL) and stored at 4 °C overnight. The white precipitate was collected and purified by column chromatography using 4% MeOH in CH_2Cl_2 to obtain the pure product $BnBz^6A$ as a white solid (40 mg, 36%).

TLC ($MeOH/CH_2Cl_2$ = 10:90); R_f = 0.68.

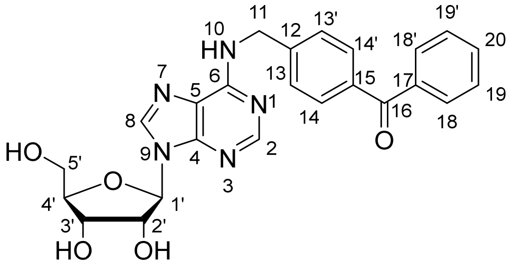

^{1}H NMR (400 MHz, DMSO-d_6) δ (ppm) = 8.60 (s, 1H, H-10), 8.41 (s, 1H, H-2), 8.22 (s, 1H, H-8), 7.72 – 7.64 (m, 5H, H-17, H-18, H-18', H-19, H-19'), 7.56 – 7.49 (m, 4H, H-13,H-13',H-14, H-14'), 5.90 (d, J = 6.1 Hz, 1H, H-1'), 5.46 (d, J = 6.2 Hz, 1H, 2'-OH), 5.37 (dd, J = 7.0, 4.5, 1H, 5'-OH), 5.20 (d, J = 4.6 Hz, 1H, 3'-OH), 4.81 (s, 2H, H-11), 4.64 – 4.60 (q, J = 5.9 Hz, 1H, H-2'), 4.15 (td, J = 4.7, 3.0 Hz, 1H, H-3'), 3.97 – 3.95 (m,1H, H-4'), 3.70 – 3.65 (dt, J = 12.1, 4.2 Hz, 1H, H-5'a), 3.58– 3.52 (ddd, J = 12.0, 7.1, 3.7 Hz, H-5'b).

^{13}C NMR (101 MHz, DMSO-d_6) δ (ppm) = δ 195.95 (C-16), 154.73 (C-6), 152.84 (C-2), 148.99 (C-4), 145.70 (C-17), 140.57 (C-8), 137.63 (C-15), 135.90 (C-12), 133.05, (C-20) 130.32 (2C, C-19, C-19'), 130.03 (2C, C-18, C-18'), 129.02 (2C, C-13, C-13'), 127.51 (2C, C-14, C-14'), 120.27 (C-5), 88.38 (C-4'), 86.37 (C-4'), 73.93 (C-2'), 71.10 (C-3'), 62.10 (C-5'), 43.25 (C-11).

HR-MS (ESI⁺): Exact mass calculated for $C_{24}H_{23}N_5NaO_5$ $[M+Na]^+$: 484.15914, found: 484.15830.

2.10.4 4-Azidobenzyl bromide (40)

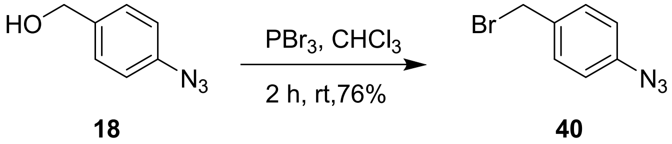

4-Azidophenyl)methanol **18** (300 mg, 2.00 mmol, 1.00 eq.) in $CHCl_3$ (6 mL) under nitrogen atmosphere. PBr_3 (228 µL, 650 mg, 2.40 mmol, 1.20 eq) was added dropwise to the reaction mixture at room temperature. The reaction was quenched with a saturated aqueous solution

of $NaHCO_3$ (3 mL). The organic layer was washed with water (6 mL). The combined organic layers were dried over anhydrous Na_2SO_4 and the solvent was removed *in vacuo.* The residue was purified by column chromatography on silica gel to obtain the desired product **40** as a brown solid (330 mg, 76%).

TLC (EtOAc/*n*-hexane = 40:60); R_f = 0.69.

^{1}H NMR (400 MHz, $CDCl_3$) δ (ppm) = 7.40 – 7.36 (m, 2H, Ar-H), 7.02 – 6.98 (m, 2H, Ar-H), 4.48 (s, 2H, CH_2).

^{13}C NMR (101 MHz, $CDCl_3$) δ (ppm) = 137.85, 134.61, 129.37 (2C), 118.65 (2C), 31.33.

2.10.5 N1-azidobenzyl modified adenosine $(BnN_3)^1A$

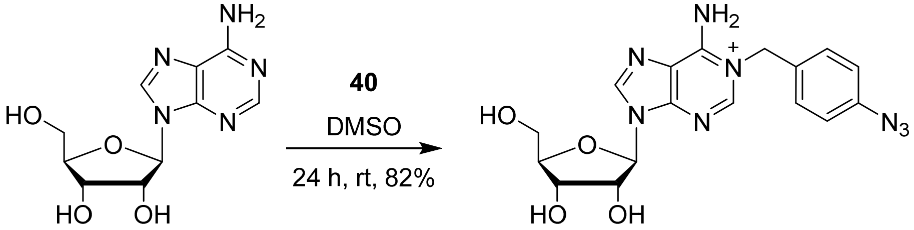

36 **$(BnN_3)^1$A**

Adenosine (50 mg, 0.19 mmol, 1.00 eq.) and 4-azidobenzyl bromide **38** (48 mg, 0.22 mmol, 1.20 eq.) was suspended in DMSO (1 mL). After vigorously stirring at room temperature for 24 h, the reaction mixture was lyophilized. The crude residue was purified by silica gel column chromatography using 10% MeOH in CH_2Cl_2 to obtained pure off-white solid **$(BnN_3)^1$A** (62 mg, 82%)

TLC ($MeOH/CH_2Cl_2$ = 20:80); R_f = 0.34.

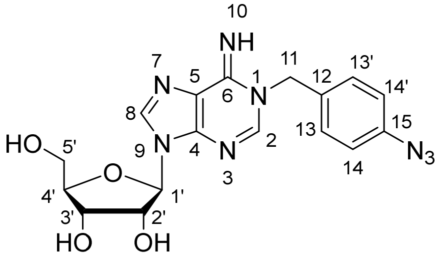

^{1}H NMR (400 MHz, DMSO-d_6) δ (ppm) = 8.64 (s, 1H, H-2), 8.51 (s, 1H, H-8), 7.42 – 7.38 (m, 2H, H-13, H-13'), 7.14 – 7.11 (m, 2H, H-14, H-14'), 5.87 (d, *J* = 5.6, 1H, H-1'), 5.54 (d, *J* = 6.0, 1H, 2'-OH), 5.43 – 5.39 (s, 2H), 5.26 (d, *J* = 5.0 Hz, 1H, 3'-OH), 5.11– 5.08 (m,1H, 5'-OH), 4.49 (q, *J* = 5.6 Hz, 1H, H-2'), 4.15 – 4.12 (td, *J* = 4.9, 3.5 Hz, 1H, H-3'), 3.97 – 3.95 (dd, *J* = 3.9 Hz, 1H, H-4'), 3.69 – 3.64 (m, 1H, H-5'a), 3.58 – 3.53 (m, 1H, H-5'b).

^{13}C NMR (101 MHz, DMSO-d_6) δ (ppm) = 151.59 (C-6), 148.19 (C-2), 144.38 (C-4), 140.91 (C-8), 139.22 (C-12), 132.06 (C-15), 129.35 (2C, C-13, C-13'), 120.90 (C-5), 119.44 (2C, C-14, C-14'), 87.83 (C-1'), 85.89 (C-4'), 74.30 (C-2'), 70.24 (C-3'), 61.19 (C-5'), 50.30 (C-11).

HR-MS (ESI$^-$): Exact mass calculated for $C_{17}H_{17}N_8O_4$ $[M-H]^-$: 397.13782, found: 397.13874.

2.10.6 4-Azidobenzylamine (42)[13]

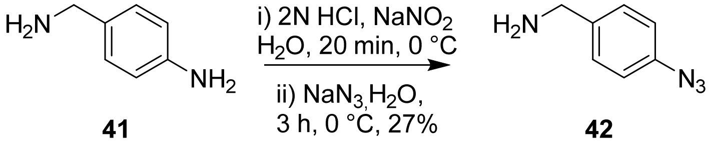

41 **42**

To an ice-cooled solution of 4-aminobenzylamine **41** (500 mg, 4.1 mmol, 1.00 eq.) in 2.0 M aqueous HCl (3.5 mL), $NaNO_2$ (283 mg, 4.1 mmol, 1.00 eq.) in water (0.7 mL) was added dropwise. After stirring for 20 min at 0˚C, NaN_3 (267 mg, 4,1 mmol) in water (0.7 mL) was added, and the stirring was continued for 3 h at 0˚C. The reaction was quenched with saturated aqueous $NaHCO_3$ (3 mL), extracted with EtOAc (3 x 3 mL), and the organic layer was dried over anhydrous $MgSO_4$. The obtained crude brown solid was used for further reactions without purification.

TLC ($MeOH/CH_2Cl_2$ = 20:80); R_f = 0.51.

^{1}H NMR (400 MHz, $CDCl_3$) δ (ppm) = 7.40– 7.36 (m, 2H, Ar-H), 7.02– 6.98 (m, 2H, Ar-H), 4.48 (s, 2H, CH_2).

^{13}C NMR (101 MHz, $CDCl_3$) δ (ppm) = 137.85, 134.61, 129.37 (2C), 118.65 (2C), 31.33.

2.10.7 *N*^{6}-azidobenzyl modified adenosine $(BnN_3)^6A$

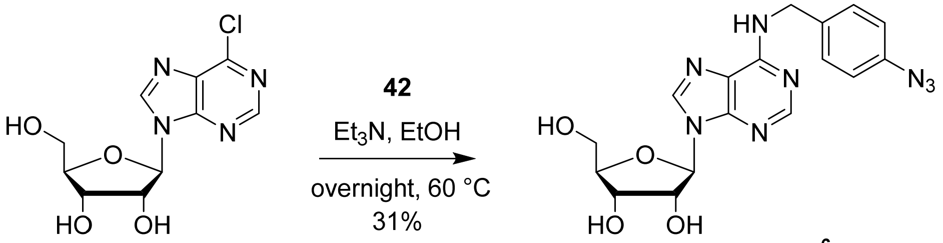

39 **$(BnN_3)^6A$**

6-Chloropurine nucleoside **39** (70 mg, 0.24 mmol, 1.00 eq.) and 4-azidobenzylamine **42** (54 mg, 0.36 mmol, 1.50 eq.) were suspended in ethanol (3 mL) and Et_3N (132 µL, 0.96 mmol, 4.00 eq.) was added to the suspension. The mixture was stirred overnight at 60 °C. solvent was removed *in vacuo.* The crude residue was purified by silica gel column chromatography using 5% MeOH in CH_2Cl_2 to obtain the pure product **$(BnN_3)^6A$** as an off-white solid (30 mg, 31%).

TLC ($MeOH/CH_2Cl_2$ = 20:90); R_f = 0.72.

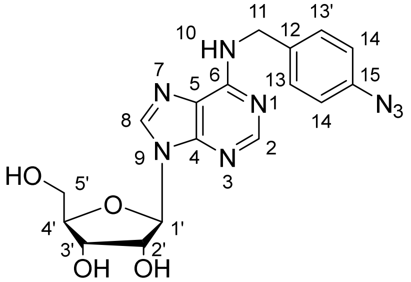

^{1}H NMR (400 MHz, DMSO-*d*$_6$) δ (ppm) = 8.50 (s, 1H, H-10), 8.38 (s, 1H, H-8), 8.20 (s, 1H), 7.37 (d, *J* = 8.2 Hz, 2H, H-13, H-13'), 7.07 – 7.03 (m, 2H, H-14, H-14'), 5.89 (d, *J* = 6.2 Hz, 1H, H-1'), 5.45 (d, *J* = 6.2 Hz, 1H, 2'-OH), 5.38 (dd, *J* =7 .2, 4.5 Hz, 1H, 5'-OH), 5.29 (d, *J* = 4.6 Hz, 1H, 3'-OH), 4.64 (s, 2H, H-11), 4.60 (d, *J* = 5.9 Hz, 1H, H-2'), 4.16 – 4.12 (m, 1H, H-3'), 3.97 – 3.95 (m, 1H, H-4'), 3.69 – 3.64 (m, 1H, H-5'a), 3.57 – 3.52 (m, 1H, H-5'a).

^{13}C NMR (101 MHz, DMSO-*d*$_6$) δ (ppm) = 154.42 (C-6), 152.36 (C-2), 148.50 (C-4), 140.03 (C-8), 137.68 (C-12), 128.83 (2C, C-13, C-13'), 119.72 (C-5), 118.97 (2C, C-14, C-14'), 87.93 (C-1'), 85.91 (C-4'), 73.48 (C-2'), 70.66 (C-3'), 61.66 (C-5'), 42.38 (C-11).

HR-MS (ESI$^+$): Exact mass calculated for $C_{17}H_{18}N_8NaO_4$ $[M+Na]^+$: 421.13381, found: 421.13432.

3. Solid phase synthesis, deprotection and purification of RNA oligonucleotides

RNA oligonucleotides were prepared by solid-phase synthesis using phosphoramidite chemistry with 2′-O-TOM-protection on controlled pore glass (CPG) solid support. RNA sequences are shown in Tables S3. Modified phosphoramidites (2′-OMe-U, 2′-OMe-C) were purchased. 5'-Hexynyl, *N*^{6}-methyl adenosine (m^{6}A) and *N*^{6}-benzyl Adenosine (Bn^{6}A) phosphoramidite were prepared by following published procedures.[17] RNA sequences are given in the Table S3. RNA oligonucleotides were cleaved from the solid support and deprotected with 1 mL of 1:1 $MeNH_2$ and aq. NH_3 (AMA) at 37 °C for 5 h. The solid support was filtered off and the solvent was removed *in vacuo*. The crude oligonucleotide samples were dissolved in freshly prepared in 1 mL TBAF solution in THF (1 M) and shaken overnight at 37 °C. After addition of 1 M Tris-HCl pH 8.0 (1 mL), THF was removed *in vacuo* and the oligonucleotide samples were desalted by size exclusion chromatography using an ÄKTA start purification system with three HiTRAP Desalting columns (5 mL volume each) from GE Healthcare. The desalted oligonucleotides were eluted with water and a flow rate of 2 mL/min. The water was removed under reduced pressure and the samples were dissolved in fresh nanopure water. The crude oligonucleotides were purified using 20% denaturing polyacrylamide gel electrophoresis (PAGE) and recovered by extraction with TEN buffer (10 mM Tris-HCl, 0.1 mM EDTA, 300 mM NaCl, pH 8.0) at 37 °C for 3 h. The pure RNA oligonucleotides were precipitated with 70% ethanol and their purity was confirmed by anion-exchange HPLC. Measured and calculated masses are listed in the Table S3.

4. In vitro transcription reaction to generate the tRNA

The dsDNA template for in vitro transcription of the 77-nt *E.coli* tRNA-Asp was assembled from two ssDNA oligonucleotides (D3 + D4) by overlap extension using Klenow exo$^-$ The reaction was performed at 37 °C for 2 h using 200 pmol of each primer with 0.2 mM of each dNTP and 2.5 U Klenow Polymerase exo in 50 µL 1x Klenow buffer. Following PCI extraction and EtOH-precipitation of the fully assembled template, 100 pmol of the dsDNA was used for 100 µL of in vitro transcription with T7 RNA polymerase. The dsDNA template was mixed with 4 mM of each NTP, 40 mM Tris (pH = 8), 30 mM $MgCl_2$, 10 mM DTT, 2 mM spermidine and 4 µL of T7 RNA polymerase (4 mg/µL). After overnight incubation at 37 °C, the reaction was quenched by the addition of 20 µL EDTA (0.5 M, pH 8) and 80 µL loading dye. The transcript was purified on 10% denaturing PAGE and recovered by extraction with TEN buffer (10 mM Tris-HCl, 0.1 mM EDTA, 300 mM NaCl, pH 8.0) at 37 °C for 3 h.

5. Fluorescent labeling of oligonucleotides

5.1 3’-end labeling of RNAs using aminolysis of NHS-ester

Oligonucleotides with 3'-amino group were labeled with 6-carboxyfluorescein succinimidyl ester (NHS-fluorescein). Amino-modified substrate RNA (**R1**) (5 nmol in 5 µL of H_2O) was dissolved in carbonate buffer (40 µL, 100 mM, pH = 9.0) and solution of NHS-fluorescein in DMF (10 µL, 7.5 mM) was added. The reaction mixture was incubated at 37°C for 2 h in the dark. The labeled RNA was purified by 20% denaturing PAGE.

5.2 3' end labeling of substrate RNA using periodate glycol oxidation

The substrate RNA (**R5**) was labeled using periodate oxidation followed by a reaction with fluorescein-5-thiosemicarbazide (Flu-TSC). The substrate RNA (4 nmol) was dissolved in 8 µL of 5x sodium phosphate buffer (8 µL, 250 mM, pH 7.4) and a freshly prepared aqueous solution of $NaIO_4$ (2 µl, 400 mM). Nanopure water was added to a total volume 40 µl. The resulting mixture was incubated for 10 min at 37 °C. The excess of $NaIO_4$ was quenched by treatment

with aqueous Na_2SO_3 (4 µL, 1 M) for 5 min at 37 °C. Afterwards, a solution of Flu-TSC (4 µl, 100 mM) in DMF was added, and the reaction mixture was incubated in the dark for 1 h at 37 °C. The labeled product was purified on 20% denaturing PAGE.

5.3 5' end labeling using CuAAC

A solution of 5'-hexynyl-modified RNA oligonucleotide (R8–R10) (500 µM, 1.00 eq.) in water/DMSO/*t*-BuOH 4:3:1 (10 µL) was treated with a solution of 6-FAM azide (5 mM, 10 eq.) in DMSO. A solution of CuBr (5 mM, 10 eq.) and TBTA (10 mM, 20.0 eq.) in DMSO/^{t}BuOH 3:1 was added and the sample was incubated in the dark at 37 °C for 3 h. The labeled oligonucleotides were purified on 20% PAGE. The purity and identity of the labeled RNAs was confirmed by anion-exchange HPLC and HR-ESI-MS.

6. Kinetic assays of RNA-catalyzed RNA alkylation reactions

Fluorescein-labeled target RNA (10 pmol) was mixed with the corresponding 1:1 mixture of 5'- and 3'-fragments of ribozyme (100 pmol each) in 1x reaction buffer (120 mM KCl, 5 mM NaCl and 50 mM Bis-Tris, pH 6.0) in a total volume of 10 µL. To ensure proper folding and formation of the ribozyme-substrate RNA complex, an annealing step (3 min at 95 °C and 15 min at 25 °C) was performed prior to the addition of cofactor (100 µM) and $MgCl_2$ (5 mM). Addition of $MgCl_2$ was performed last and marked the start of the reaction. The mixture was incubated at 25 °C and 1 µL aliquots were taken at desired time points and quenched immediately by adding 4 µL of stop solution (80% formamide (99%), 89 mM Tris (pH 8), 89 mM boric acid, 50 mM EDTA (pH 8)). 2.5 µL of each time-point sample was analyzed on 20% denaturing PAGE, and band intensities were quantified by fluorescence imaging using blue epi illumination and a 530/28 nm emission filter. The yield versus time data were fit according to (fraction reacted) = $Y(1 - e^{-kt})$, in which k = k_{obs} and Y = final yield, using Origin (2021). All kinetic assays were carried out as two independent replicates.

7. Preparative RNA alkylation using MTR1m2

RNA-catalyzed RNA alkylation reactions were performed in a total volume of 20 µL using 1 nmol target RNA, 1.1 nmol of each 5'- and 3'-fragments of ribozyme , 100 µM of the respective guanine cofactors, and 40 mM $MgCl_2$ in 1x reaction buffer (120 mM KCl, 5 mM NaCl, 50 mM Bis-Tris, pH 6.0) for 24 h at 25 °C. An annealing step (3 min at 95 °C and 10 min at 25 °C) was performed prior to the addition of the cofactor and $MgCl_2$ to ensure proper folding of the RNA. The modified RNA product was isolated on a 20% denaturing PAGE and recovered by extraction with TEN buffer (10 mM Tris-HCl, 0.1 mM EDTA, 300 mM NaCl, pH 8.0) at 37 °C for 3 h. Isolated pure RNA products were submitted to HR-ESI-MS analysis (Bruker microOTOF-Q III, direct injection). For mass and isolated yields after purification see table S4.

8. Dimroth rearrangement and analysis by anion exchange HPLC

Dimroth rearrangement using OH^- as nucleophile was performed in a total volume of 12 µL by incubation of the modified RNA substrates (300 pmol) in Na_2CO_3 buffer (25 mM, pH 10) including EDTA (1 mM) at 60 °C for 1 h.

Rearrangement using 4-nitrothiophenol was performed as reported in literature.[14] The modified RNA substrates (300 pmol) were incubated in 10 µL buffer containing 25 mM 4-Nitrothiophenol, 5 mM Tris(hydroxypropyl)phosphine (THP), 5% (v/v) DMF (pH 6.0, adjusted with HCl) for 9h at 60°C. The buffer was prepared in a 2x concentration and stored as aliquots at -80 °C.

Following EtOH-precipitation, the samples were analyzed using anion-exchange HPLC (Dionex DNAPac PA200, 2 × 250 mm, at 60 °C; solvent A: 25 mM Tris-HCl (pH 8.0), 6 M urea;

solvent B: 25 mM Tris-HCl (pH 8.0), 6 M urea, 0.5 M $NaClO_4$; linear gradient, 0-40% solvent B, with a slope of 4% solvent B per column volume). For reaction yields see table S5.

9. LC-MS anaylsis of modified RNA substrates

RNA digestion was performed in a total volume of 20 µL by incubation of RNA (200 pmol) with 1 U Nuclease P1 (New England BioLabs) and 2 U Shrimp Alkaline Phosphatase (rSAP, New England BioLabs) at 37 °C for 1 h. The reaction was quenched by snap-freezing in liquid nitrogen. The samples were diluted to 70 µL with nanopure water and residual denatured protein was removed by extraction with $CHCl_3$ (2 x 70 µL). The aqueous layer was concentrated under reduced pressure at 35 °C for 1 h. The resulting residue was dissolved in 60 µL NH_4OAc buffer (10 mM, pH 5.3). The sample was analyzed by LC–MS, using an RP-18 column (Synergi, 4-µm Fusion-RP C18 80 Å, 250 × 2 mm; Phenomenex) at 25 °C with aqueous mobile phase A (10 mM NH_4OAc, pH 5.3) and organic mobile phase B (100% acetonitrile). The flow rate was 0.2 ml/min with a gradient of 0–5% B in 15 min, followed by 5–70% B in 30 min. The micrOTOF-Q III with an ESI ion source was operated in positive-ion mode, with capillary voltage of 4.5 kV, end plate offset of 500 V, nitrogen nebulizer pressure 1.4 bar, dry gas flow 9 l/min and dry temperature 200 °C. Data were analyzed using Data Analysis software DA 4.2 (Bruker Daltonics).

10. Fluorescence anisotropy studies

Fluorescence anisotropy measurements were performed in Quartz SUPRASIL 10×2 and 3×3 mm High Precision Cell cuvettes (Hellma Analytics) at 25 °C, using a Jasco FP8300 spectrofluorometer with manually operated polarization filters. The excitation and emission wavelengths were set to 470 and 520 nm, respectively. Measurements were performed using 0.2 µM of 6-FAM-labeled RNA (R8–R10) in anisotropy buffer (30 mM TRIS pH 7.5, 120 mM NaCl). The samples were incubated with increasing concentrations of protein for 5 min at ambient temperature prior to the respective measurements. Fluorescence anisotropy values r were calculated as,

$$r = \frac{I_{VV} - G \cdot I_{VH}}{I_{VV} + 2 \cdot G \cdot I_{VH}}$$

where IVV denotes emission intensity at vertical excitation/vertical emission and IVH emission intensity at vertical excitation/horizontal emission. The grating factor G

$$G = \frac{I_{HV}}{I_{HH}}$$

is introduced as a correction factor to account for inherent unequal sensitivities of the spectrometer toward the different polarization planes.

K_D values were obtained from nonlinear fitting of the anisotropy data according to

$$r = r_f + (r_b - r_f) \cdot \frac{[L] + K_D + [R] + \sqrt{([L] + K_D + [R])^2 - 4 \cdot [L] \cdot [R]}}{2 \cdot [L]}$$

Here, r_f and r_b denote the respective anisotropy values of free ligand and bound complex, while [L] and [R] are the total ligand (RNA) and receptor (protein) concentrations in the sample.

11. Supporting Tables

Table S1: ID and structures for alkyl guanine cofactors (**3**–**10**)

ID for O^6-alkyl derivatives	Alcohol	O^6-alkyl derivatives (3–10)	Eq. of alcohol, reaction time and yield
**prop⁶G (3)**[15] CAS NO. 188680-41-3	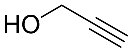	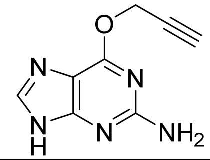	3.50 eq., 3 h, 73%
**azihex⁶G (4)**	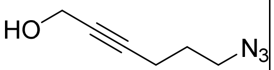	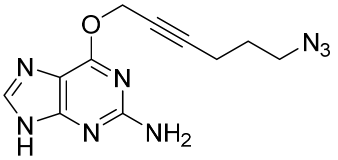	1.10 eq., 4 h, 31%
**BG-CH₂N₃ (5)**[16] CAS NO: 1151762-33-2	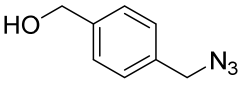	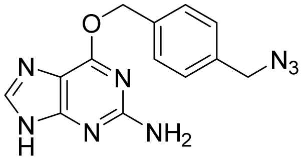	2.50 eq., overnight, 67%
**BG-prop (6)**[1] CAS NO: 680622-71-3	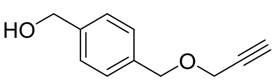	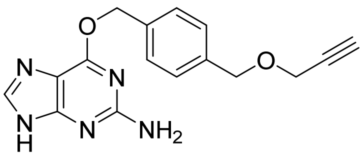	3.60 eq., overnight, 77%
**BG-DBCO (7)**[9] CAS NO: 1820756-74-8	n.a	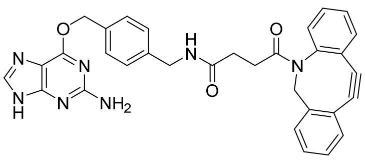	2 h, 57%
**BG-N₃ (8)**	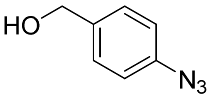	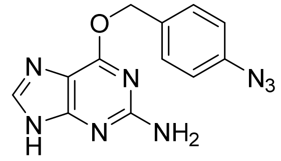	2.50 eq., 3 h, 55%
**BG-Bz (9)**	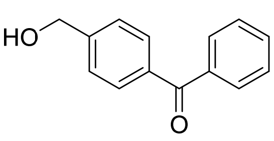	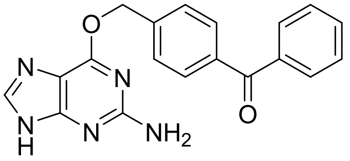	2.0 eq., overnight, 56%
**BG-AzNP (10)**[10]	n.a.	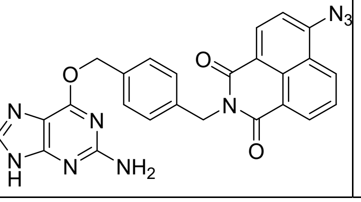	7 h, 12%

Table S2: RNAs synthesized by solid phase synthesis and respective ESI-MS analysis.

**Description**	**5'-sequence-3'**	**Calculated mass**	**Measured mass**
Substrate RNA (R1)	CCACUGAGAGCUUC-NH_2	4614.68794	4614.67570
Substrate RNA (R2)	CCACUGAGAGCUUC	4403.62643	4403.62057
MTR1 5' fragment Cm12 (R3) to target R1 and R2	GGAAGCUCUGA$\mathbf{C}_m$CGACCCCCAGCC	7635.11239	7635.13629

MTR1 3' fragment Um42 (R4) to target R1 and R2	GCUGGGACAACUAGACA$\mathbf{U_m}$ACAGUG	7749.12027	7749.14869
E. coli tRNA. fragment A58 (R5)	GGUUCGAGUCCCG	4130.57553	4130.60454
MTR1 5' fragment Cm12 (R6) to target R5	GACGGGACUGA$\mathbf{C_m}$CGACCCCCAGCC	7674.13452	7674.11999
MTR1 3' fragment Um42 (R7) to target R5	GCUGGGACAACUAGACA$\mathbf{U_m}$ACGAACCC	8342.22391	8342.25024
DRACH-RNA with Bn^{6}A (R8)	Alk-AACC**GGBn^{6}ACU**GUC	4042.63573	4042.64565
DRACH-m6A RNA with Bn^{6}A (R9)	Alk-GAUACGBn^{6}AUC**GGm^{6}ACU**GUC	5687.84937	5687.85004
DRACH-m6A RNA (R10)	Alk-GAUACGAUC**GGm^{6}ACU**GUC	5597.80242	5597.81597

Table S3: DNA oligonucleotides

**Description**	**5'-sequence-3'**
T7 promoter (D1)	CTGTAATACGACTCACTATA
Transcription template for MTR1 to target R1 and R2 (D2)	CCACTGTATGTCTAGTTGTCCCCGAGCGAACTCGGGGGGTCGGTCAGAGCTTCCTATAGTGAGTCGTATTACAG
Fw primer to assemble E. coli tRNA (D3)	CTGTAATACGACTCACTATAGGAGCGGTAGTTCAGTCGGTTAGAATACCTGCCTGTCACGCAGGGGGTC
Rv primer to assemble E. coli tRNA (D4)	TGGCGGAACGGACGGGACTCGAACCCGCGACCCCCTGCGTGACAG
Transcription template for MTR1 to target RNA R10 (D5)	GATACGTATGTCTAGTTGTCCCCGAGCGAACTCGGGGGGTCGGTCATCGGACCTATAGTGAGTCGTATTACAG

Table S4: Isolated yields for *trans*-alkylation activity of the ribozyme, molecular formulae, calculated and measured monoisotopic masses of modified RNAs.

**Description**	**Isolated yields (%)**	**Molecular formula**	**Calculated mass (g/mol)**	**Measured mass g/mol**
R2-prop	55	$C_{135}H_{168}N_{51}O_{96}P_{13}$	4441.64263	4441.71686
R2-azihex	59	$C_{138}H_{173}N_{54}O_{96}P_{13}$	4524.69098	4524.76559
R2-BnCH$_2$N$_3$	60	$C_{140}H_{173}N_{54}O_{96}P_{13}$	4548.69043	4548.67168
R2-BnPr	75	$C_{143}H_{176}N_{51}O_{97}P_{13}$	4561.69960	4561.68641
R2-DBCO	63	$C_{159}H_{188}N_{53}O_{98}P_{13}$	4809.79511	4809.88158
R2-BnN$_3$	70	$C_{139}H_{171}N_{54}O_{96}P_{13}$	4534.67533	4534.65962
R2-BnBz	87	$C_{146}H_{176}N_{51}O_{97}P_{13}$	4597.70015	4597.68489
R2-BnAzNP[a]	40	$C_{152}H_{180}N_{53}O_{98}P_{13}$	4717.73251	4717.70016
R5-BnCH$_2$N$_3$	86	$C_{131}H_{161}N_{51}O_{91}P_{12}$	4275.63897	4275.63840
R5-BnPr	75	$C_{134}H_{164}N_{48}O_{92}P_{12}$	4288.64814	4288.64261
R10-BnN$_3$	63	$C_{176}H_{217}N_{68}O_{120}P_{17}$	5728.85131	5728.84117

[a]The observed mass corresponds to NH_2 instead of N_3. This is probably due to release of N_2 during mass measurement. The expected mass for the azido modified product is 4743.72246.

Table S5: Ratio for yields of N^{1}A to N^{6}A modified RNA before and after Dimroth rearrangement as determined by anion-exchange HPLC analysis.

**Modified R2 substrate**	**N^{1}A:N^{6}A (After PAGE purification)**	**N^{1}A:N^{6}A (After Dimroth rearrangement OH$^-$)[b]**	**N^{1}A:N^{6}A (After Dimroth rearrangement S$^-$)[c]**
R2-Me	94:6	54:46	20:80
R2-Bn	89:11	56:44	13:87
R2-BnN$_3$[a]	75:10(:15)	48:34 (:18)	7:80 (:13)
R2-BnBz	82:18	53:47	11:89

[a] The amount of uncaged RNA (R1) is given in parenthesis.

[b] 25 mM Na_2CO_3, pH 10, 1 mM EDTA at 60 °C for 1 h.

[c] 25 mM 4-nitrothiophenol, 5 mM THP, 5% (v/v) DMF, pH 6 at 60 °C for 9 h.

Table S6: Isolated yields from click labeling, molecular formulae, calculated and measured monoisotopic masses of the fluorescently labeled RNA oligonucleotides R8-FAM – R10-FAM.

**Description**	**Isolated yields (%)**	**Molecular formula**	**Calculated mass (g/mol)**	**Measured mass g/mol**
R8-FAM	29	$C_{151}H_{176}N_{50}O_{90}P_{12}$	4500.75891	4500.75806
R9-FAM	53	$C_{200}H_{236}N_{69}O_{126}P_{17}$	6145.99770	6146.00645
R10-FAM	76	$C_{193}H_{230}N_{69}O_{126}P_{17}$	6055.92560	6056.03020

12. Supporting Figures

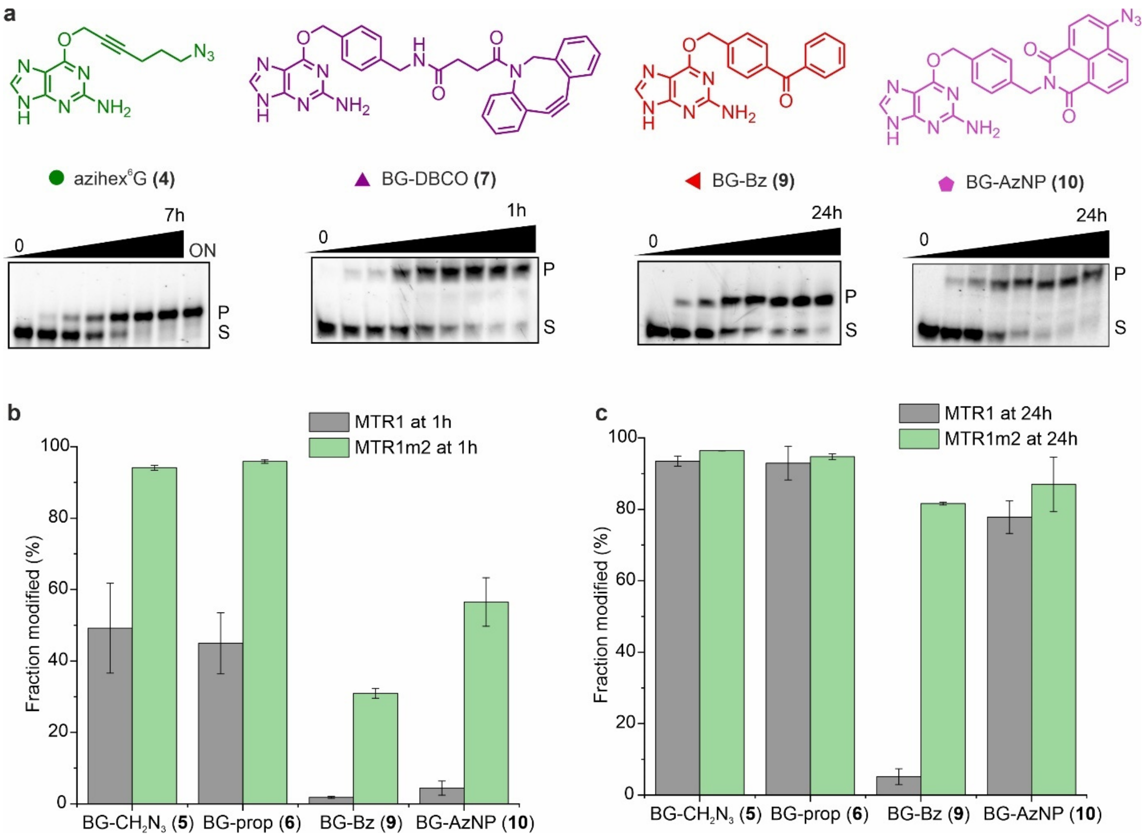

Figure S1: Representative PAGE images of the kinetic assays for cofactors **4**, **7**, **9** and **10** from two independent experiments. 1 μM 3'-fluorescein labeled RNA R1 (5′-CCACUGAGAGCUUC-NH_2-3′), 10 μM MTR1m2 (R3+R4, Table S2), 100 μM cofactor, 5 mM $MgCl_2$, pH 6.0, 25 °C, Timepoints: for azihex^{6}G: 0, 0.17, 0.5, 1, 2, 4, 7, 23 h; BG-DBCO (**7**): 0, 1, 2, 5, 10, 20, 30, 40, 60 min and for BG-Bz (**9**) and BG-AzNP (**10**): 0, 0.5, 1, 3, 5, 7, 11, 24h. Yields for modified RNA substrate using MTR1 (grey) and MTR1m2 (green) after 1 h (**b**) and 24 h (**c**) with cofactors **5**, **6**, **9** and **10**; mean of duplicate with standard error bar given. 1 μM 3'-fluorescein labeled RNA R1 (5′-CCACUGAGAGCUUC-NH2-3′), 10 μM of either MTR1 or MTR1m2, 100 μM cofactor, 5 mM MgCl2, pH 6.0, 25 °C.

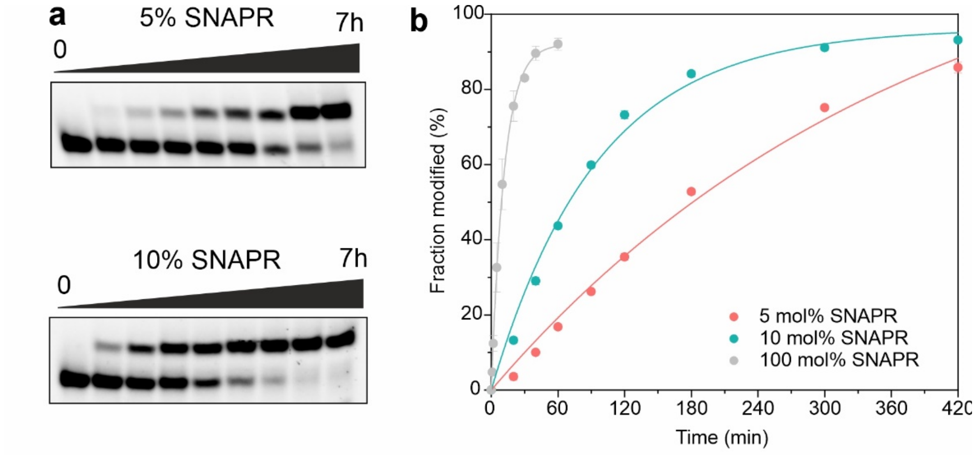

Figure S2: Analysis of multiple turnover activity of SNAPR. **a)** Representative PAGE images for analysis of RNA labeling under multiple turnover conditions. **b)** Kinetic data for labeling of 3’ FAM-labelled 14-mer RNA R1 (5’-CCACUGAGAGCUUC-NH_2-Flu3’) with p-azidomethyl-BG cofactor **5** using only 5 mol% or 10 mol% MTR1m2 relative to the target RNA. Conditions: 100 µM cofactor **5**, 5 mM $MgCl_2$, pH 6.0, 25 °C. For comparison, kinetics under single turnover conditions are shown in grey.

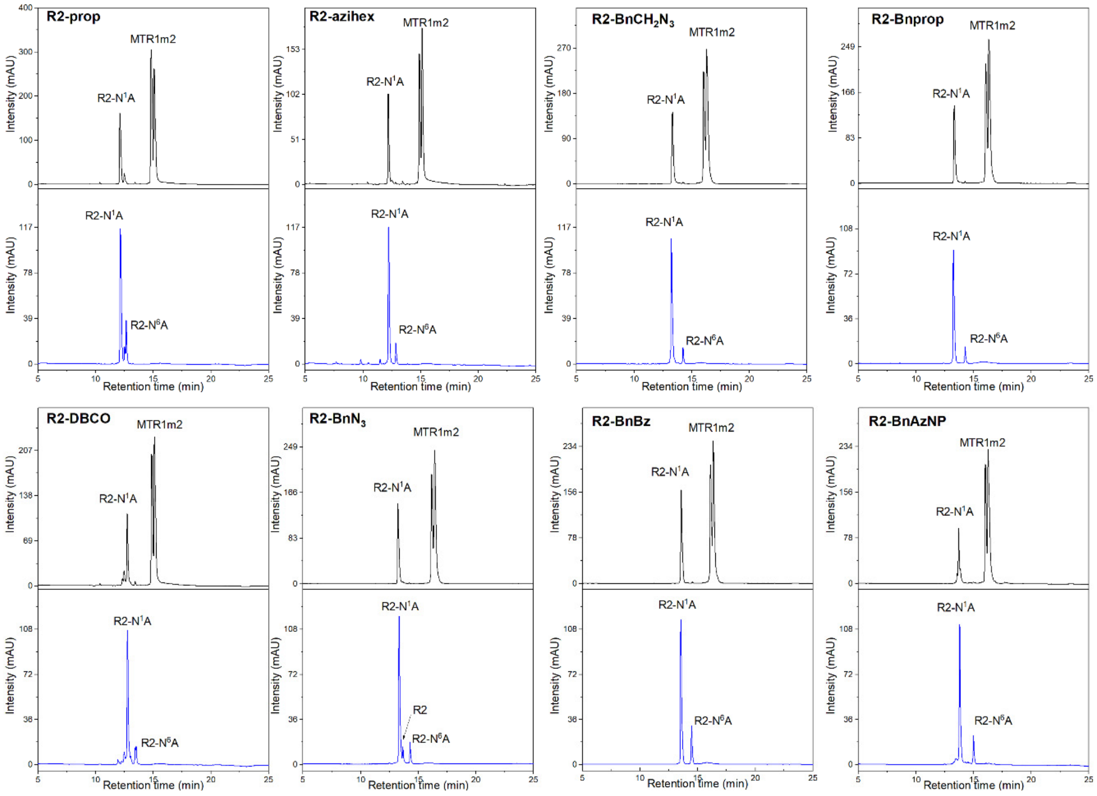

Figure S3: Anion-exchange HPLC profiles of the trans-alkylation reaction using MTR1m2 ribozyme and different guanine-based cofactors before (black) and after (blue) PAGE purification. Reaction conditions: 1 nmol substrate RNA (R2), 1.1 nmol MTR1m2, 100 µM cofactor, 1x reaction buffer (120 mM KCl, 5 mM NaCl, 50 mM Bis-Tris, pH 6.0), 40 mM $MgCl_2$, 25 °C, 24 h.

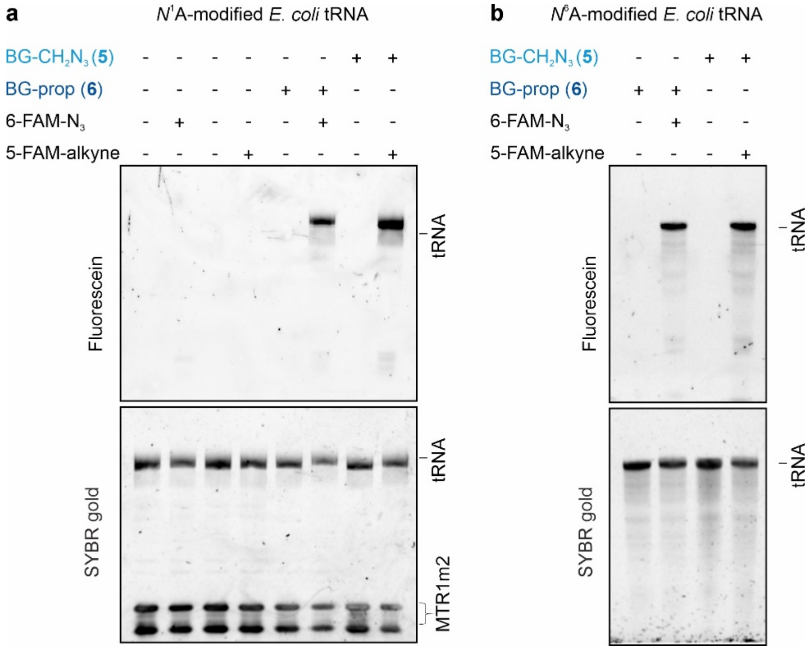

Figure S4: SNAPR-catalyzed labeling of *E. coli* tRNA-Asp. (**a**) expanded version of Figure 3d for CuAAC labeling of *E. coli* tRNA-Asp modified by SNAPR using cofactors **5** or **6** resolved on denaturing PAGE. Conditions: 50 µM 6-FAM-N_3 or 5-FAM-alkyne, 0,5 mM CuBr, 1 mM TBTA, 37 °C, 3 h. The gel was imaged in the fluorescein-channel (top) to detect the labeled tRNAs. Then it was stained with SYBR gold (bottom) to visualize the tRNA and the two SNAPR fragments. **b**) CuAAC labeling of E.coli tRNA-Asp modified by SNAPR as in a, then subjected to Dimroth rearrangement conditions with NTPh at pH6 to generate A58-N6-alkylated tRNA.

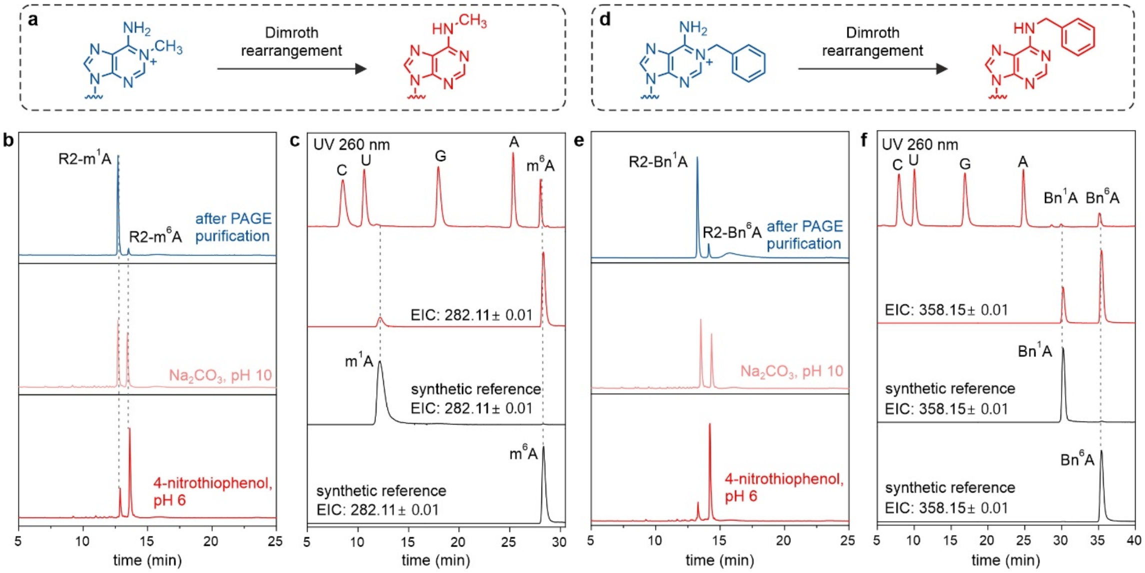

Figure S5: Dimroth rearrangement on *N*[1]-modified RNAs (14-nt), R2-m^{1}A (**a**) and R2-Bn^{1}A (**d**). Anion-exchange HPLC profile for harsh (light red) and mild (red) Dimroth rearrangement conditions on m^{1}A modified R2 (**b**) and Bn^{1}A modified R2. (**e**). LC-MS analysis of digested RNA R2 after Dimroth rearrangement for R2-m^{6}A (**c**) and R2-Bn^{6}A (**f**). Digestion of RNA (200 pmol) performed with nuclease P1 and shrimp alkaline phosphatase at 37 °C for 1 h.

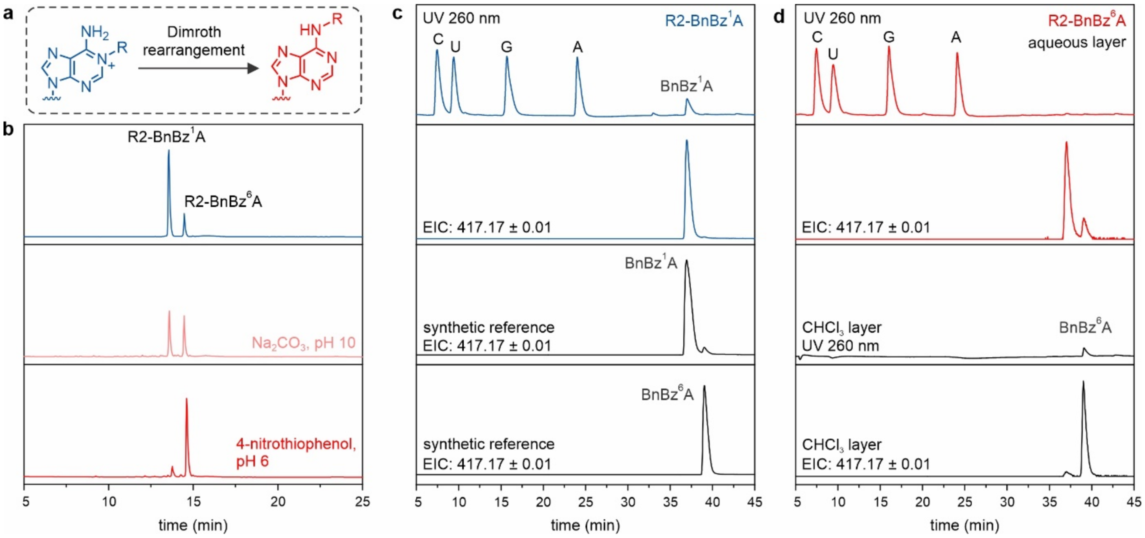

Figure S6: (**a**) Dimroth rearrangement converts *N*1- to *N*6-modified adenosine RNA R2 (14-nt). (**b**) Anion-exchange HPLC analysis of isolated *N*1-product from SNAPR reaction of R2 (5′-CCACUGAGAGCUUC-3′) with BG-Bz (**9**) after Dimroth rearrangement reactions using Na_2CO_3 (pH 10), 60 °C, 1h (pink) or 4-nitrothiophenol (pH 6), 60 °C, 9 h (red). (**c**) LC-MS analysis of the digested BG-Bz modified RNA (R2-BnBz^{1}A). The UV trace (260 nm) and extracted ion chromatogram (EIC, detecting $m/z = 417.17 \pm 0.01$) are shown. Digestion of RNA (200 pmol) performed with nuclease P1 and shrimp alkaline phosphatase at 37 °C for 1 h. (**d**). LC-MS analysis of digested BG-Bz modified R1 after Dimroth rearrangement (R2-BnBz^{6}A). The UV trace (260 nm) and extracted ion chromatogram (EIC, detecting $m/z = 417.17 \pm 0.01$) are shown. After extraction of the digested nucleosides, BnBz^{6}A was only detected in the organic, but not in the aqueous layer due to its low solubility in aqueous solvents.

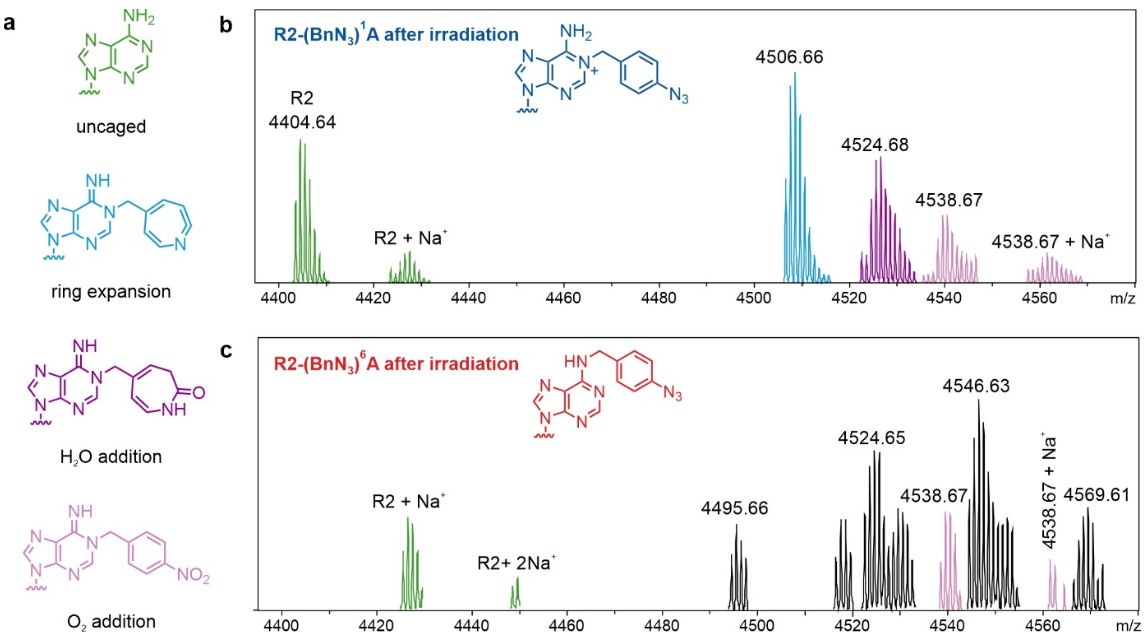

Figure S7: (**a**) Intermediates formed during Irradiation of R2-$(BnN_3)^1$A and R2-$(BnN_3)^6$A. (**b**) and (**c**) Mass analysis for R2-$(BnN_3)^1$A and R2-$(BnN_3)^6$A after irradiation at 312 nm in H_2O (200 pmol for 5 min). The mass corresponding to the starting material R2-$(BnN_3)^1$A and R2-$(BnN_3)^6$A (4534.66 a.u.) was not observed after irradiation.

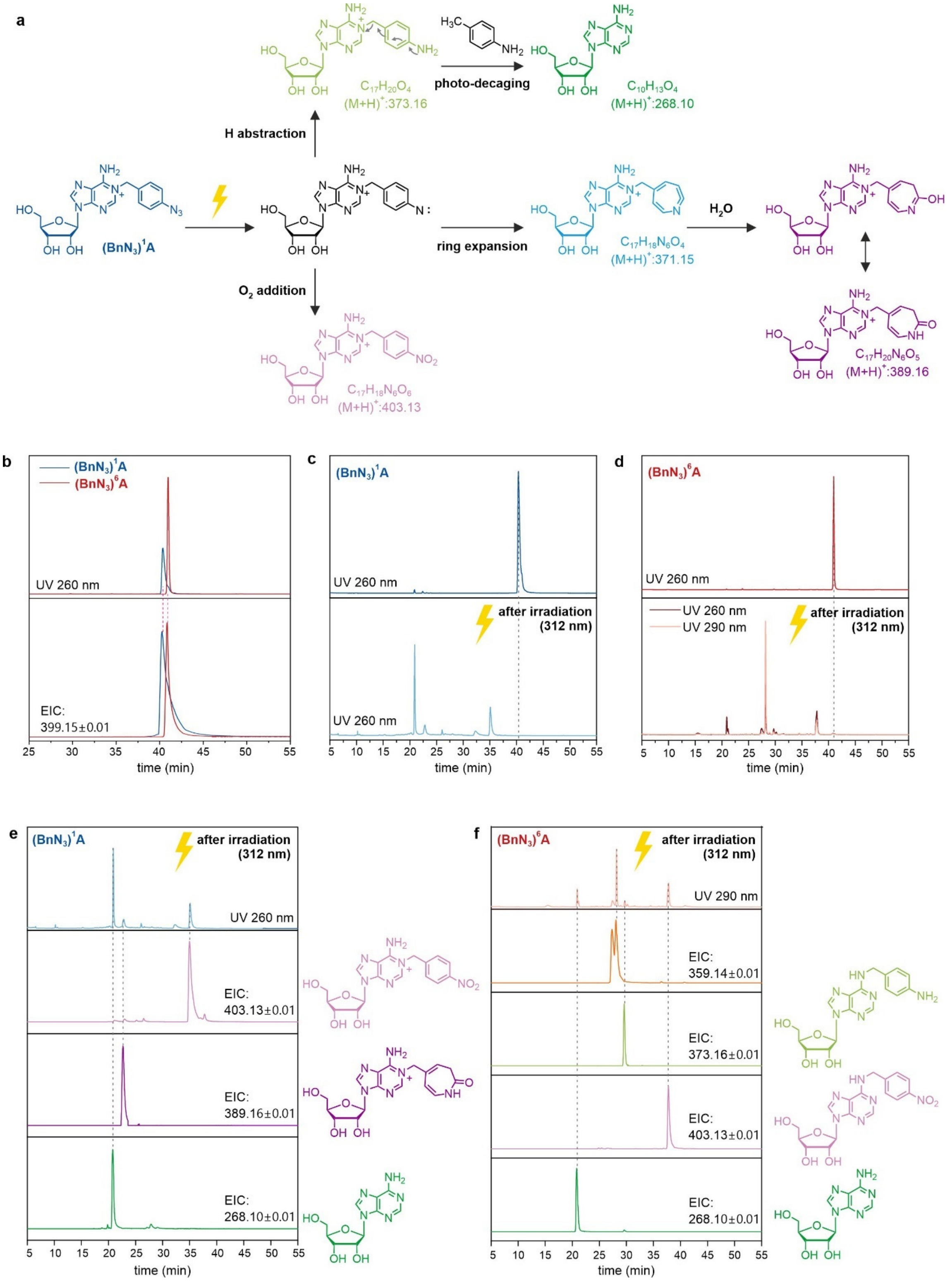

Figure S8: (**a**) Possible reaction pathways including intermediates formed for the photo-uncaging of $(BnN_3)^1A$ after exposure to light. (**b**) LC-MS for $(BnN_3)^1A$ and $(BnN_3)^6A$ nucleosides (**c**), (**d**) LC-MS profile for $(BnN_3)^1A$ and $(BnN_3)^6A$ before and after irradiation at 312 nm. (**e**), (**f**) LC-MS profiles for $(BnN_3)^1A$ and $(BnN_3)^6A$ after irradiation at 312 nm reveals the formation of different intermediates.

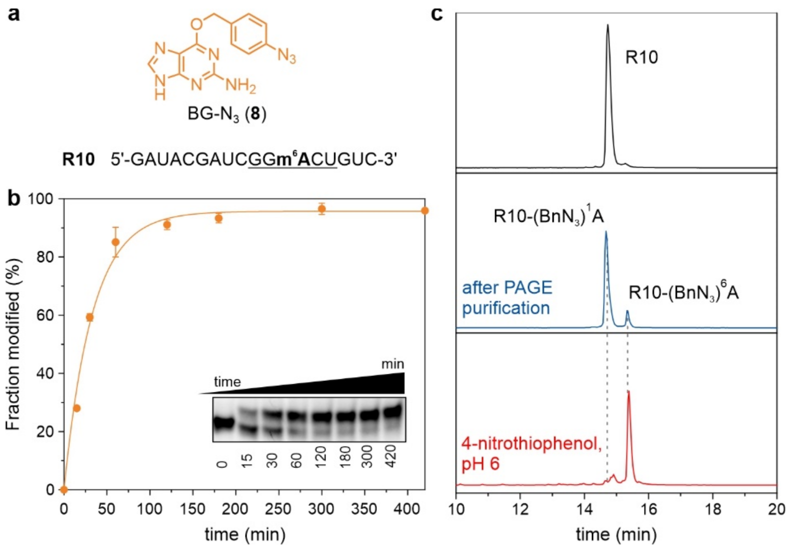

Figure S9: (**a**) Structure of cofactor and sequence of substrate RNA R10 (17-nt) containing the GGm^{6}ACU motif (**8**). (**b**) Time course of MTR1m2 catalyzed modification of R10 using cofactor **8** fit to a pseudo-first order kinetic. Insert shows a representative gel image of two independent replicates for the reaction analyzed on 20 % denaturing PAGE (100 µM **8**, 40 mM $MgCl_2$, pH 6, 25 °C (**c**) Anion-exchange HPLC profile for *trans*-alkylation with cofactor **8** before and after Dimroth rearrangement (100 pmol R10-(BnN_3)^{1}A, 25 mM 4-nitrothiophenol, 5 mM THP in 5% (v/v) DMF, pH 6.0, 60 °C, 9 h). The yield for R10-(BnN_3)^{6}A was 84%.

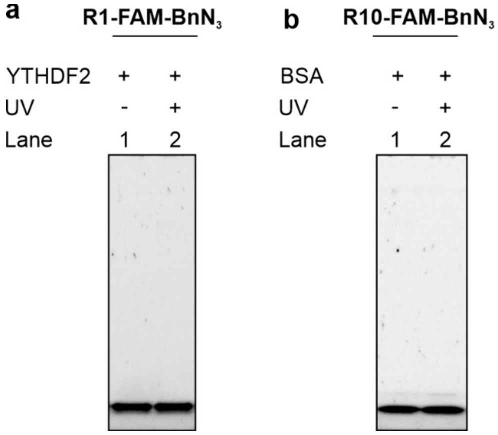

Figure S10: SDS page analysis for controls of photo-crosslinking. (**a**) Random RNA R1-FAM-BnN_3 not containing the GGm^{6}ACU motif (**b**) R10-FAM-BnN_3 together with BSA protein shows no crosslinking.

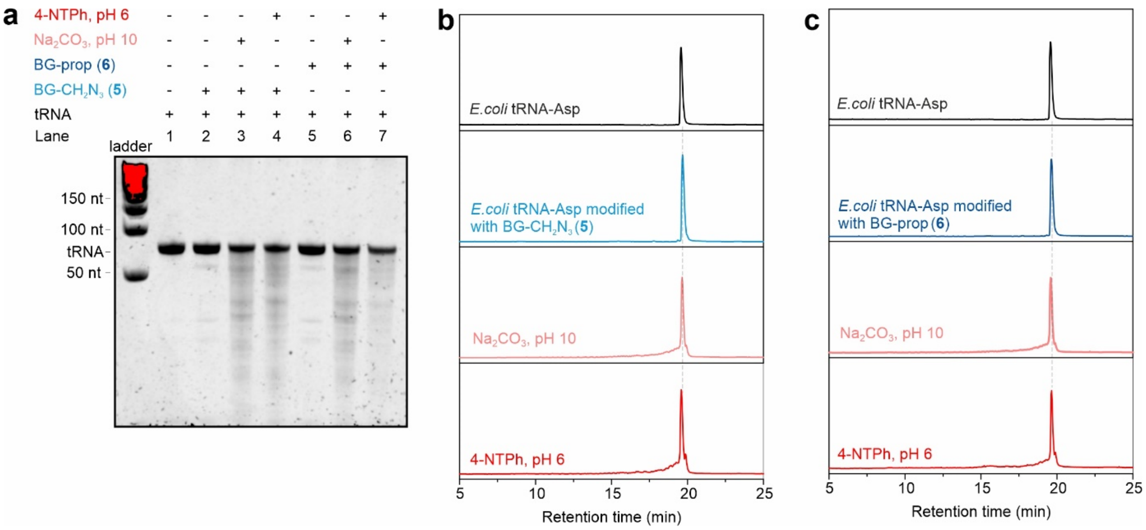

Figure S11. Dimroth rearrangement for converting N^1-alkylated-A58 into N^6-alkylated A58, analyzed by **a**) PAGE and **b**,**c**) anion exchange HPLC.

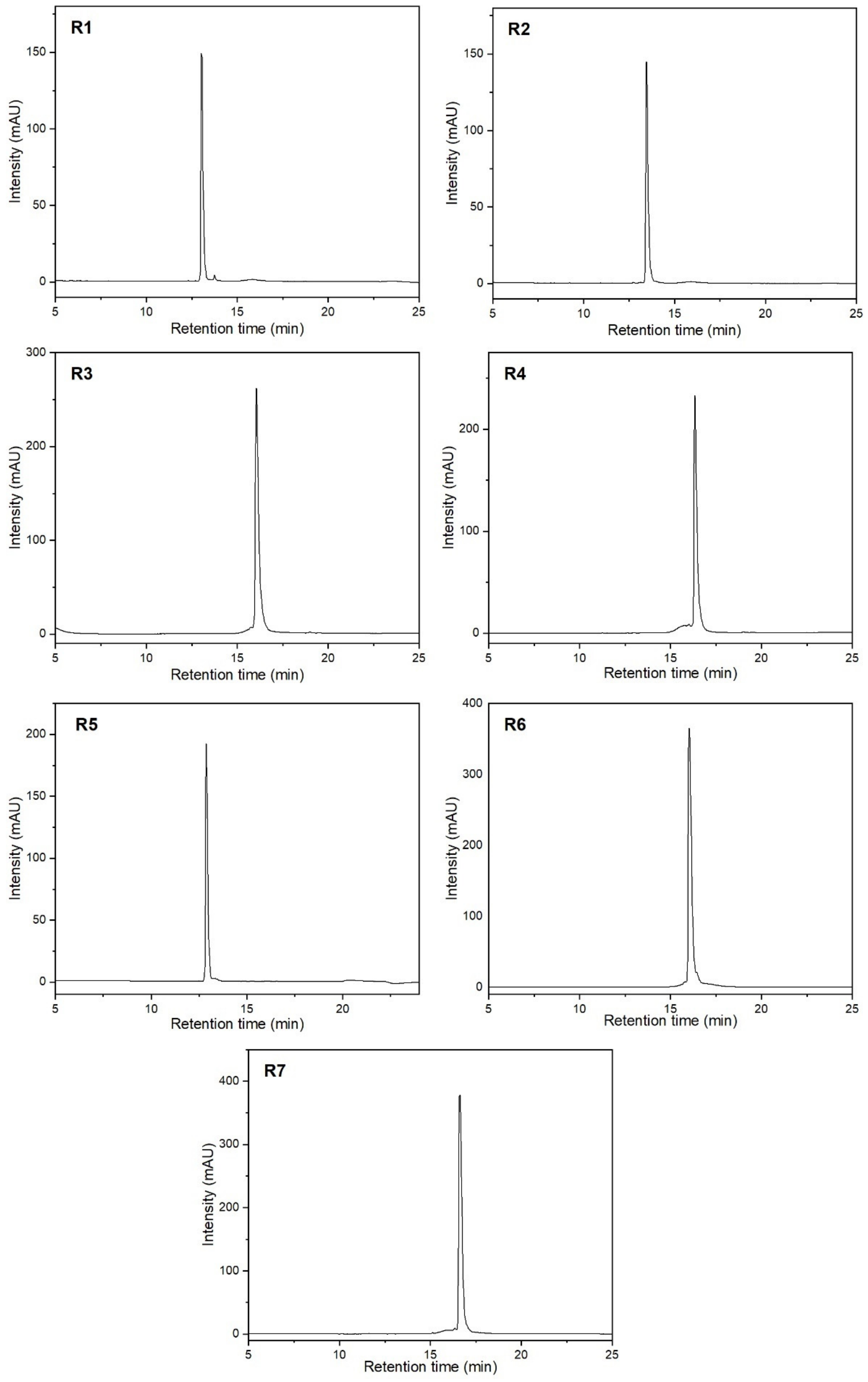

Figure S12: Anion-exchange HPLC profile for RNA R1-R7 (260 nm) to confirm purity of the RNAs.

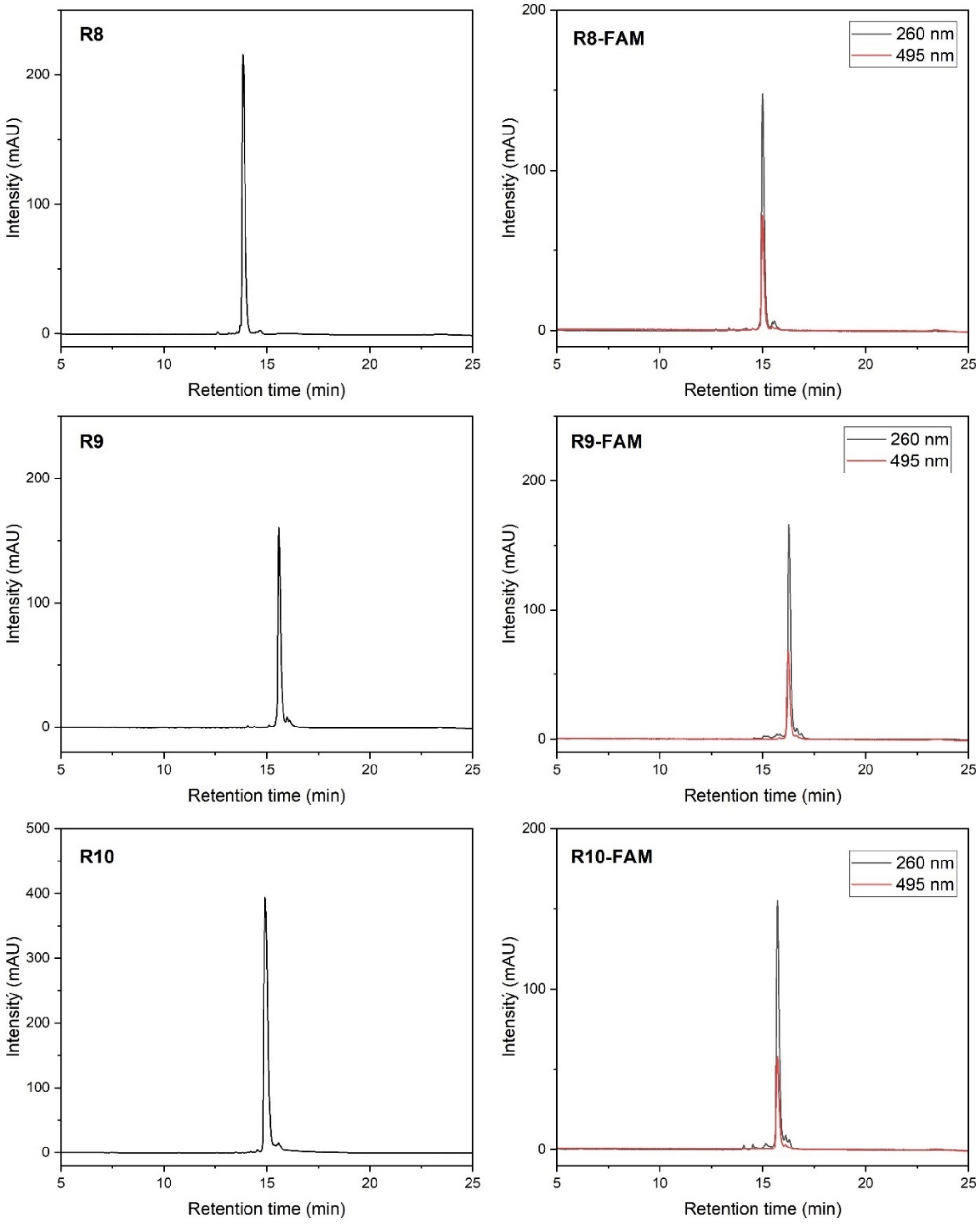

Figure S13: Anion-exchange HPLC profile for RNA R8-R10 (260 nm) and FAM-labeled R8-R10 (260 and 495 nm) to confirm purity of the RNAs.

13 NMR spectra

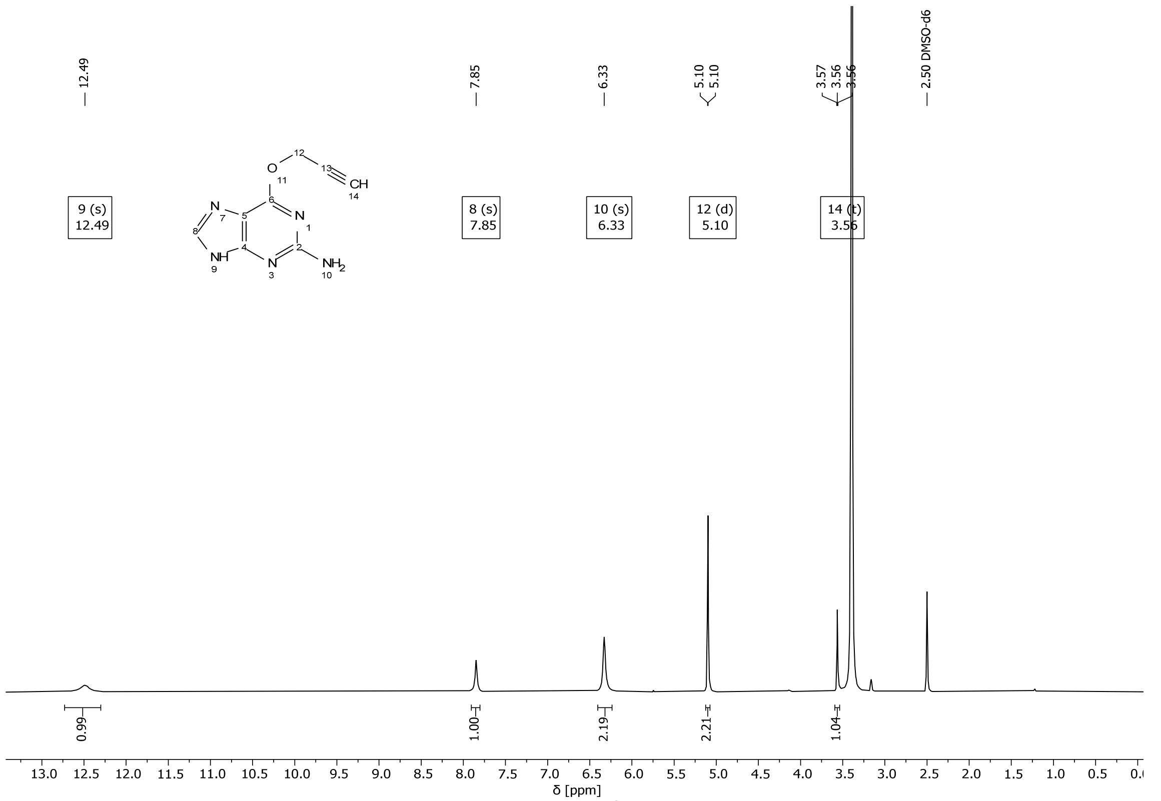

^{1}H NMR (400 MHz, DMSO-d_6) of compound **prop^{6}G (3)**.

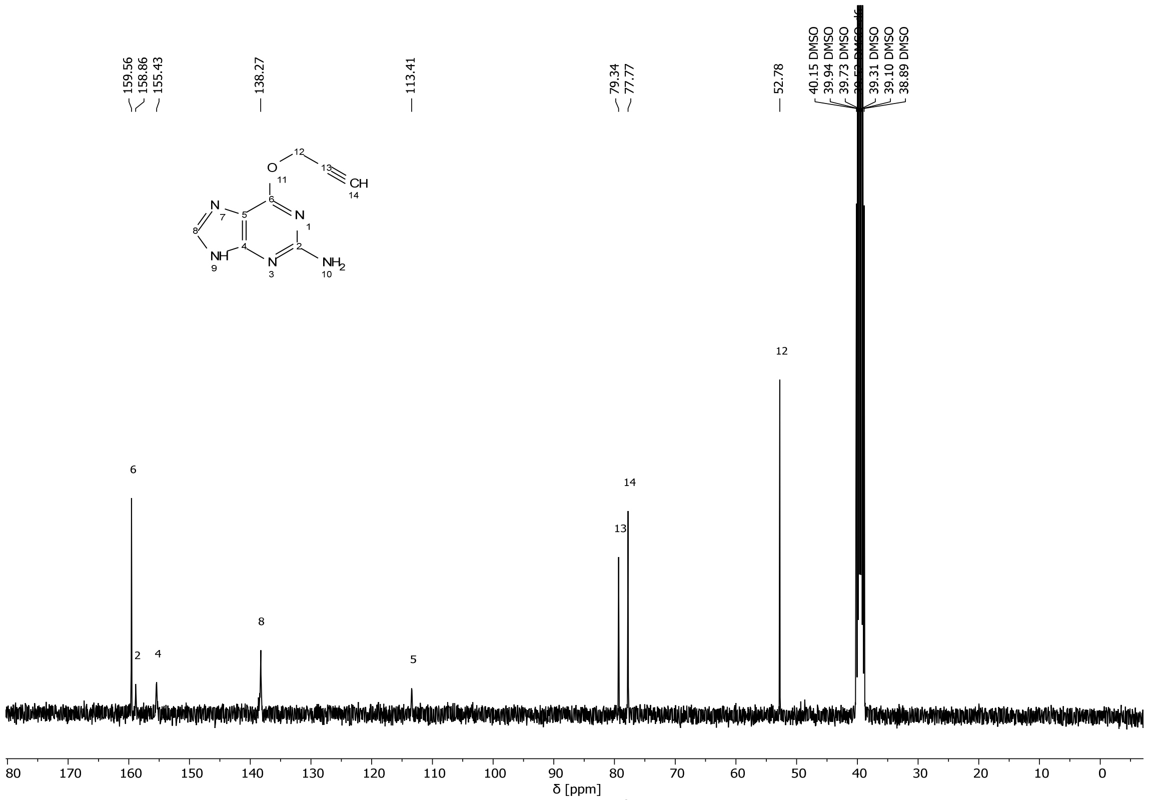

^{13}C NMR (101 MHz, DMSO-d_6) of compound **prop^{6}G (3)**.

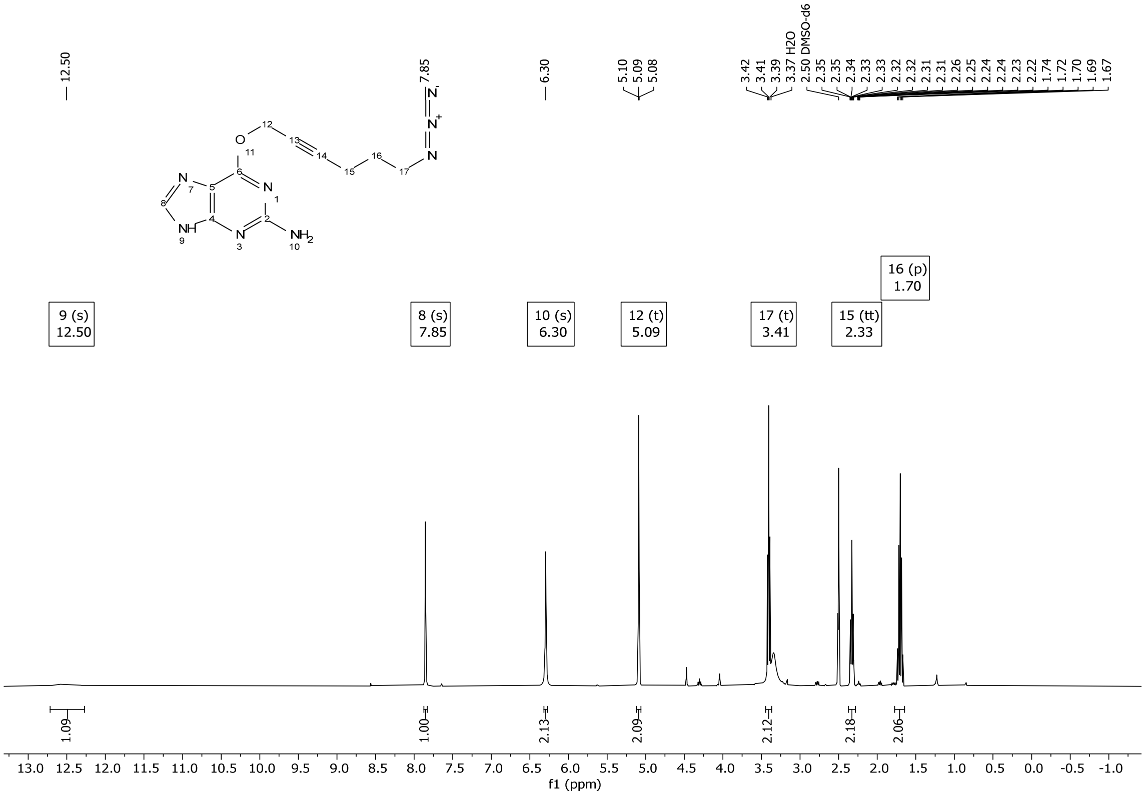

^{1}H NMR (400 MHz, DMSO-*d6*) of compound **azihex^{6}G (4)**.

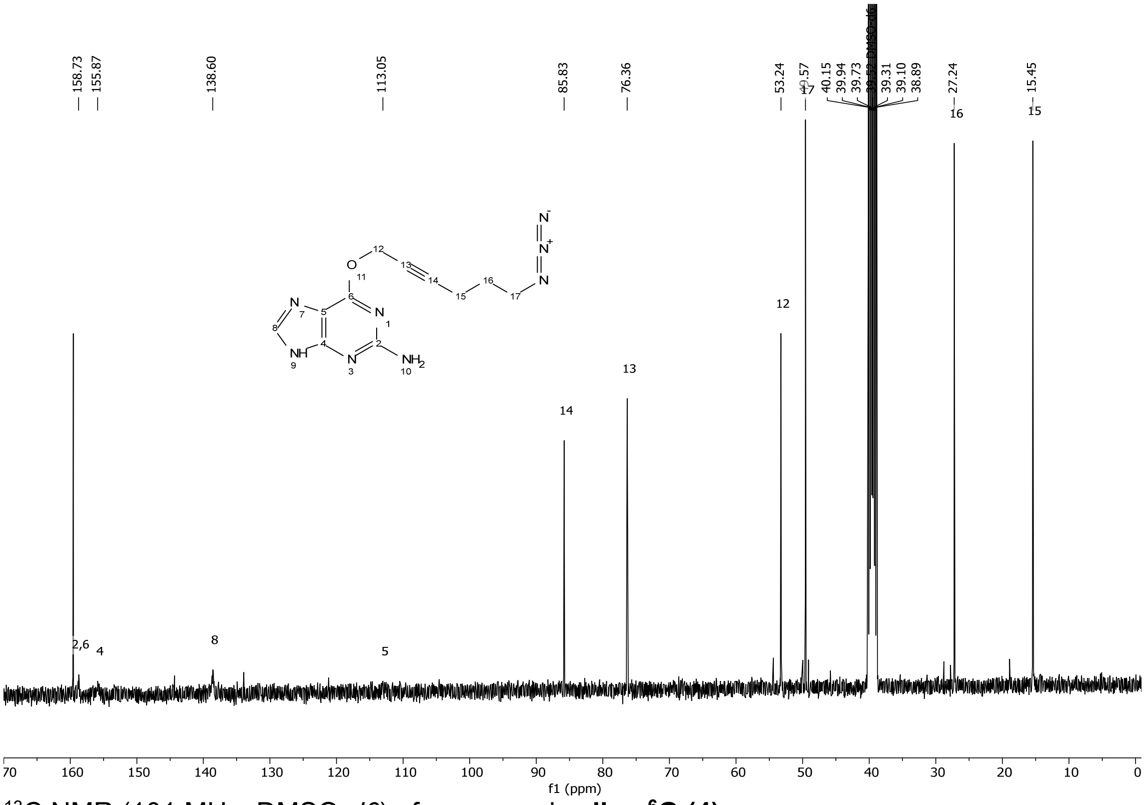

^{13}C NMR (101 MHz, DMSO-*d6*) of compound **azihex^{6}G (4)**.

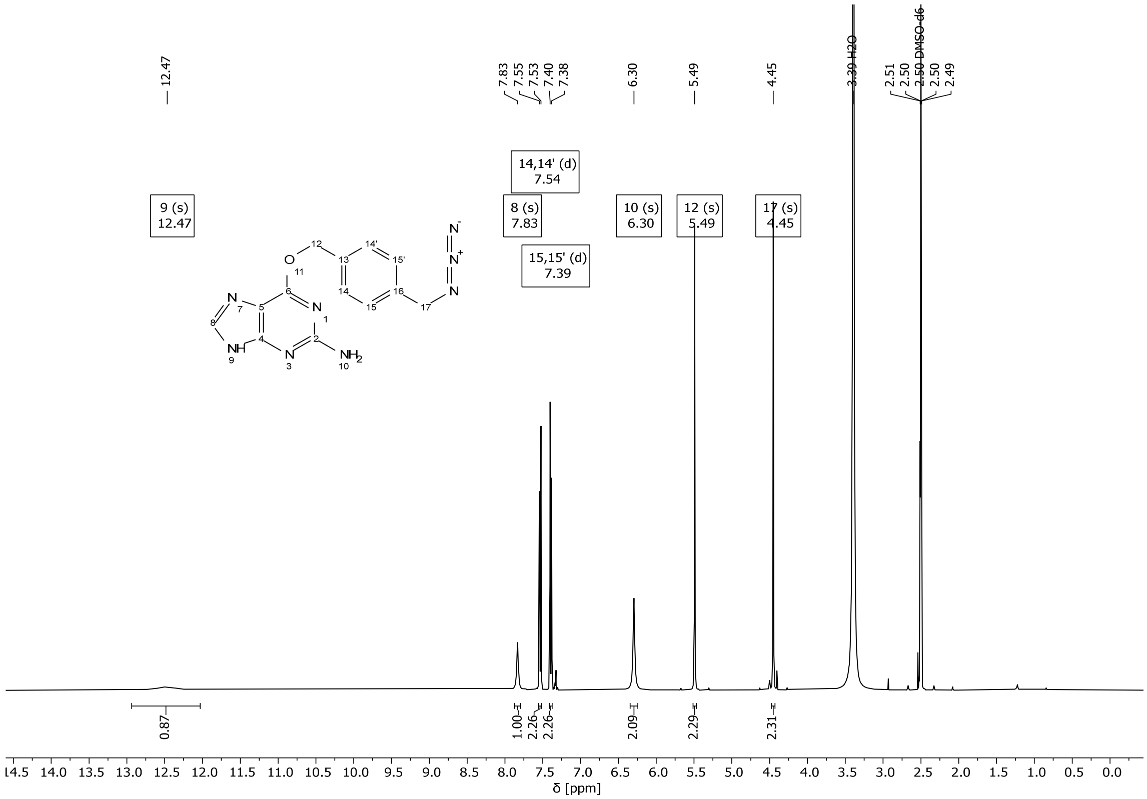

^{1}H NMR (400 MHz, DMSO-d_6) of compound **BG-CH$_2$N$_3$ (5)**.

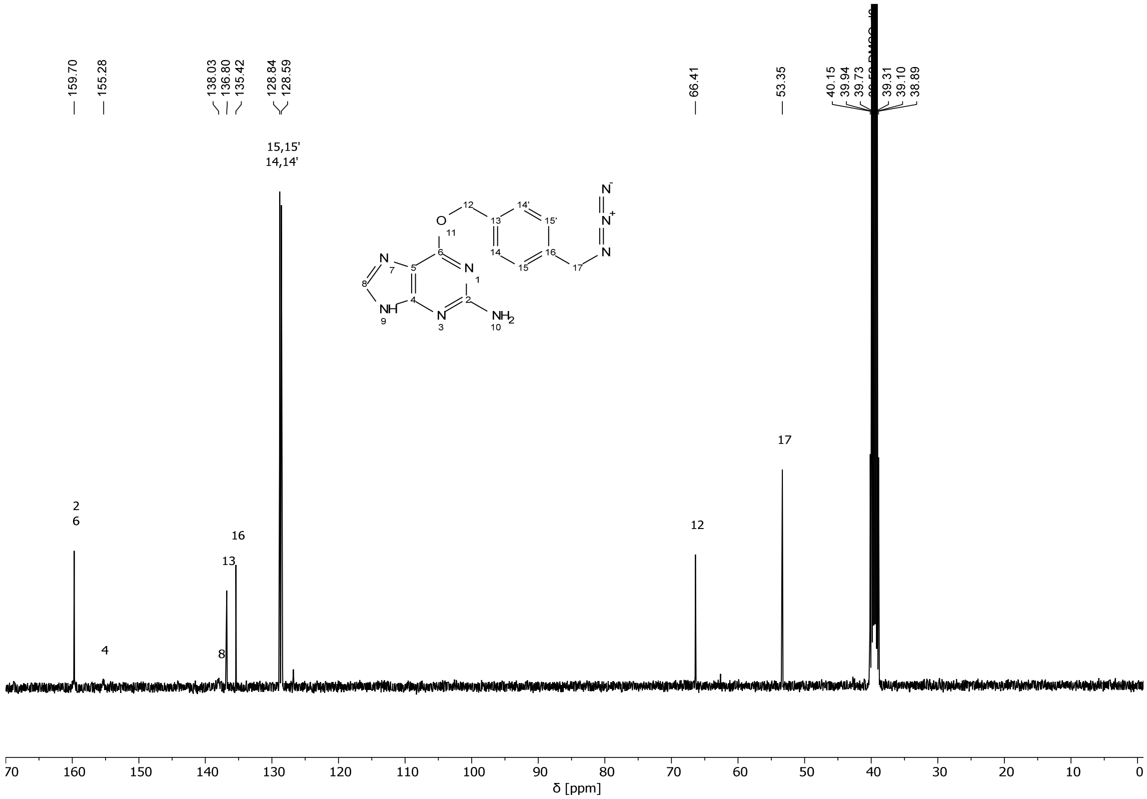

^{13}C NMR (101 MHz, DMSO-d_6) of compound **BG-CH$_2$N$_3$ (5)**.

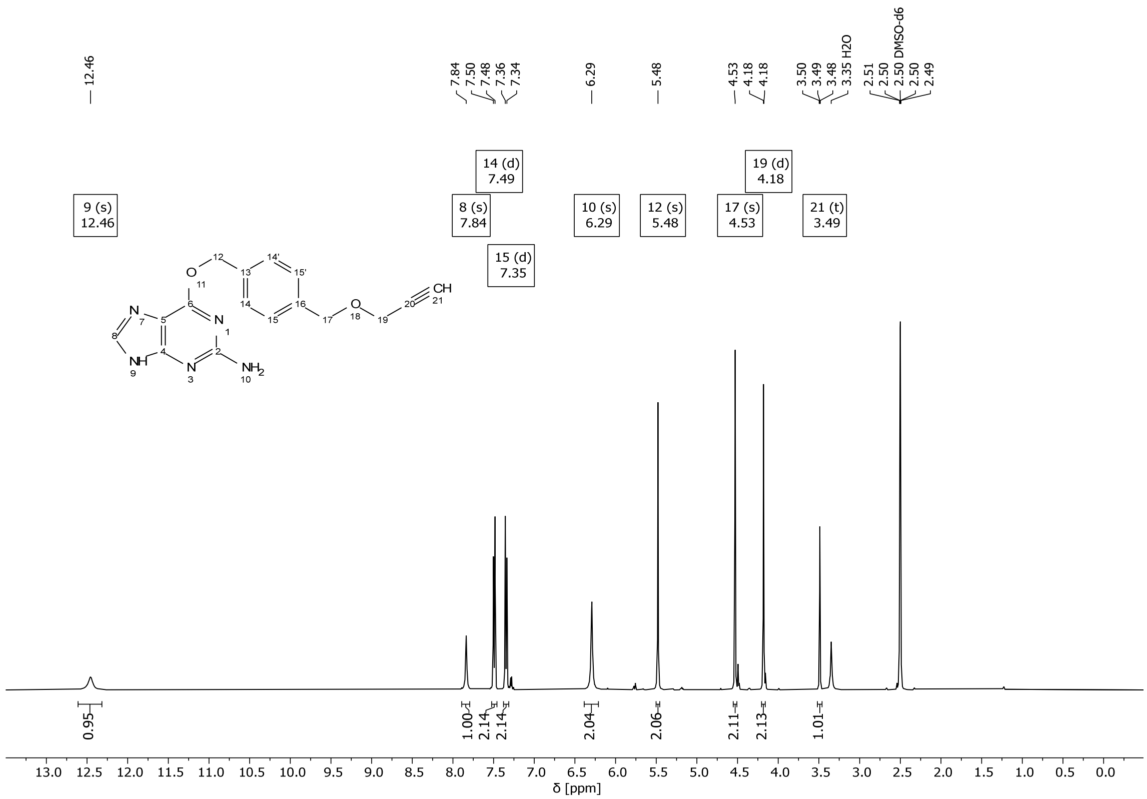

^{1}H NMR (400 MHz, DMSO-d_6) of compound **BG-prop (6)**.

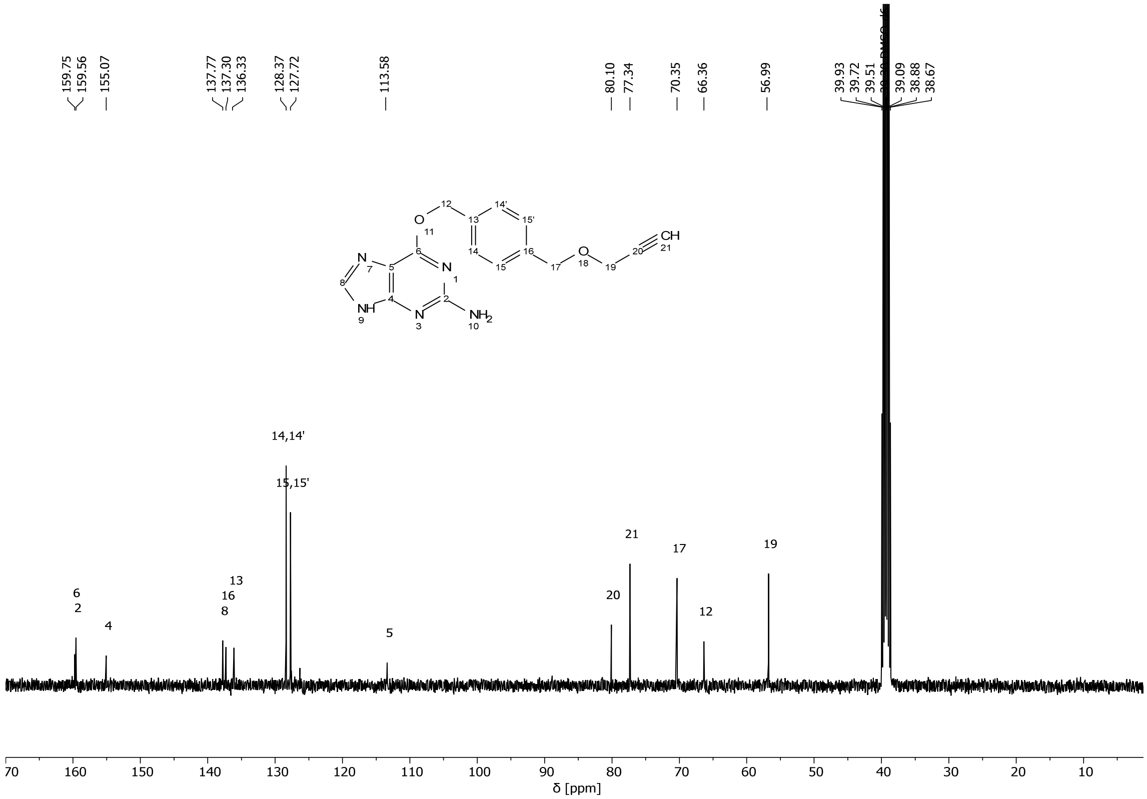

^{13}C NMR (101 MHz, DMSO-d_6) of compound **BG-prop (6)**.

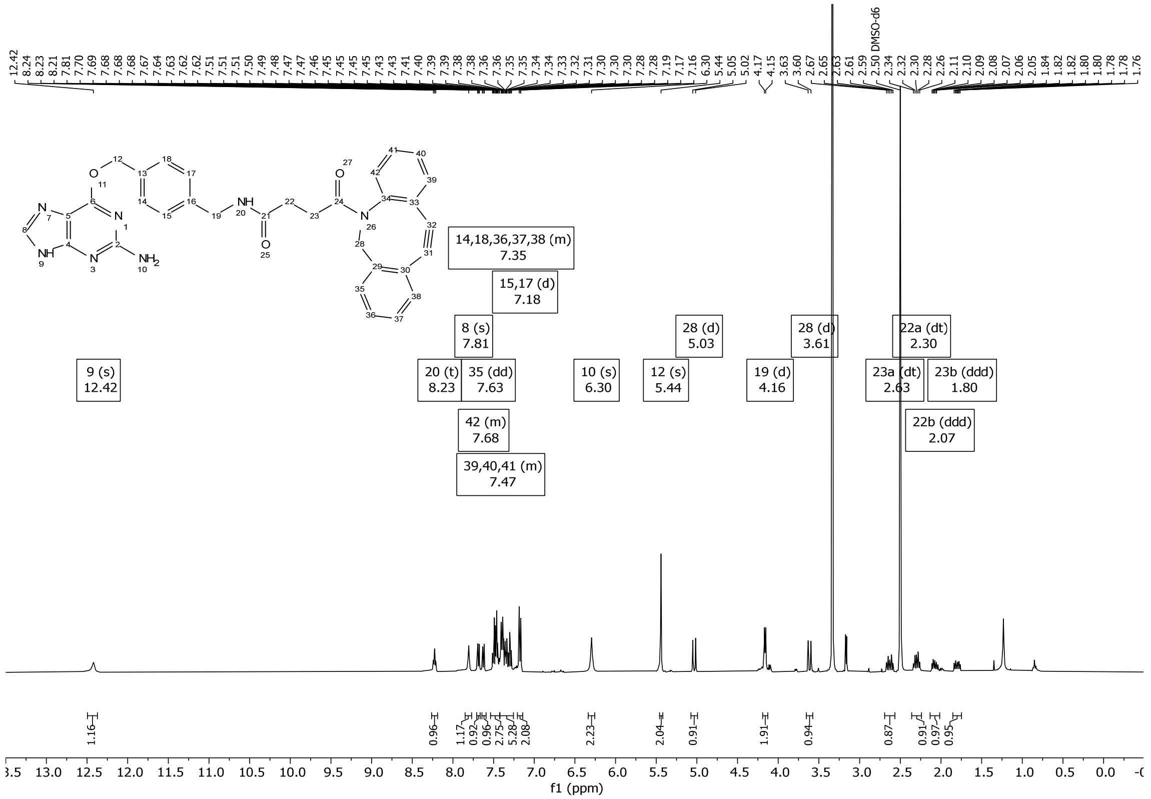

^{1}H NMR (400 MHz, DMSO-d_6) of compound **BG-DBCO (7)**.

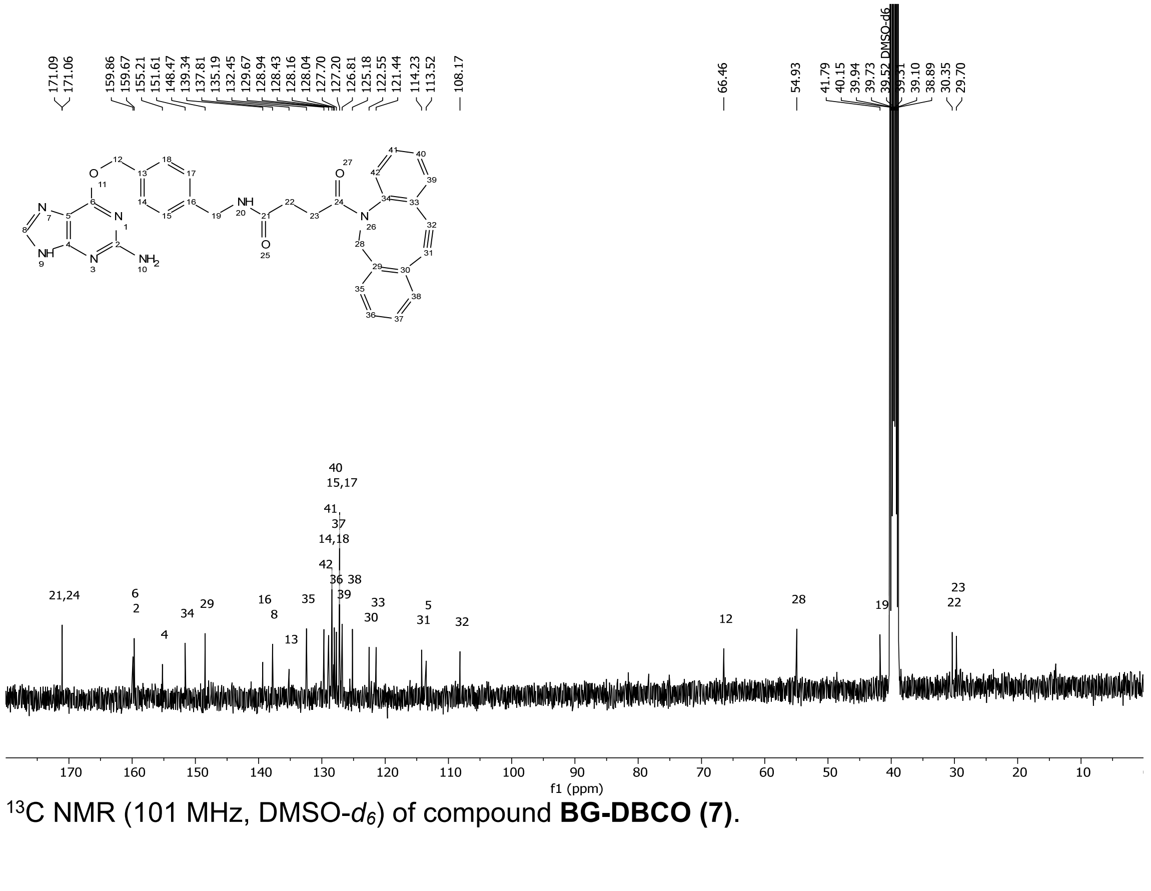

^{13}C NMR (101 MHz, DMSO-d_6) of compound **BG-DBCO (7)**.

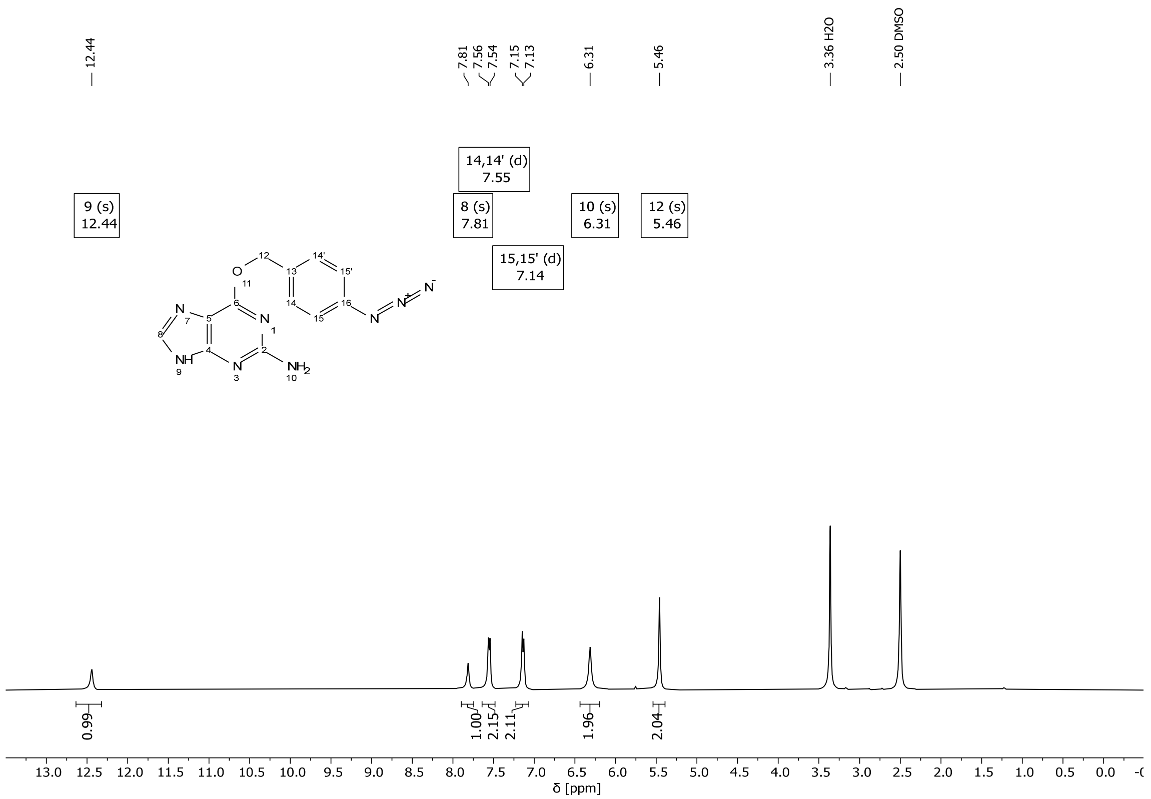

^{1}H NMR (400 MHz, DMSO-d_6) of compound **BG-N₃ (8)**.

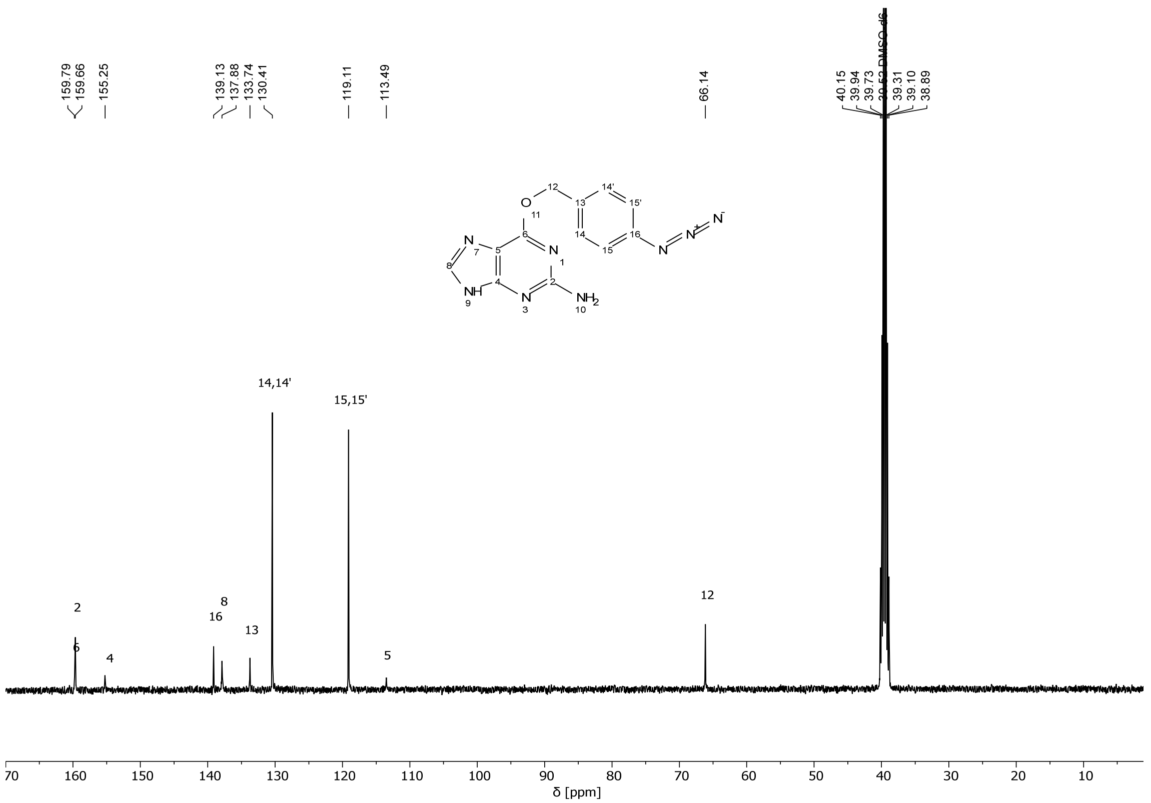

^{13}C NMR (101 MHz, DMSO-d_6) of compound **BG-N₃ (8)**.

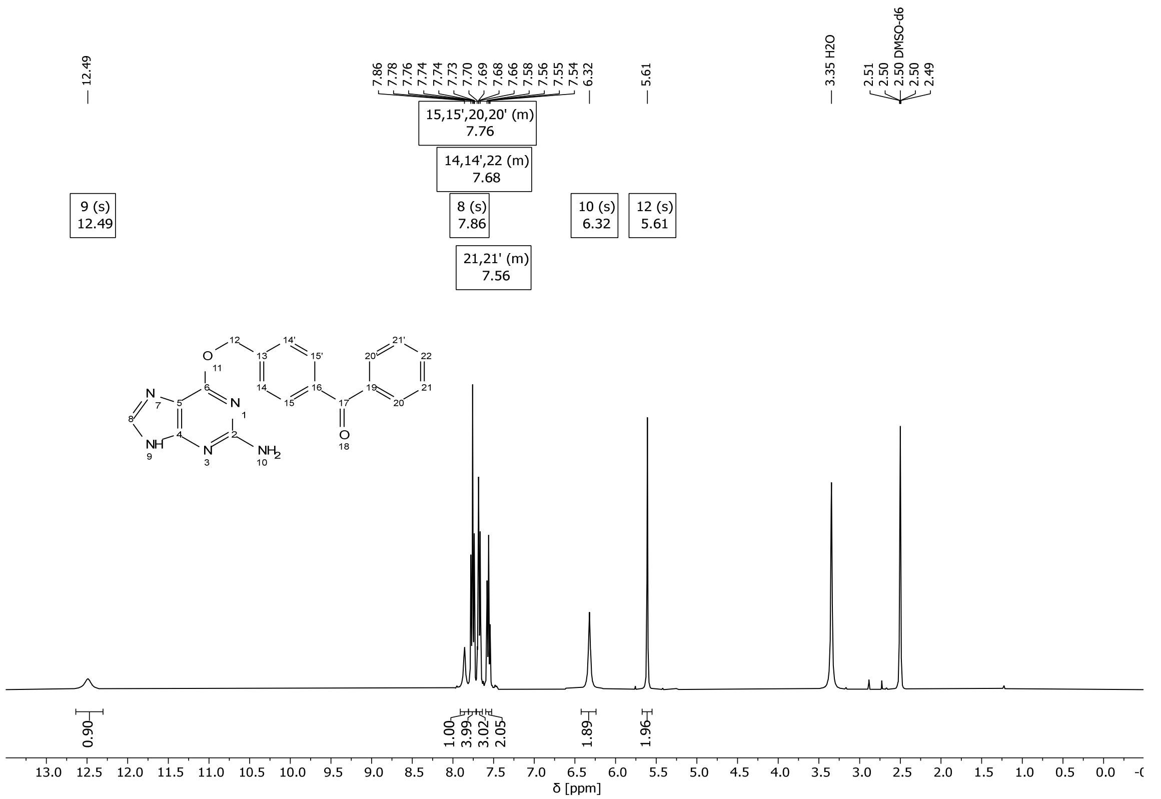

^1H NMR (400 MHz, DMSO-d_6) of compound **BG-Bz (9)**.

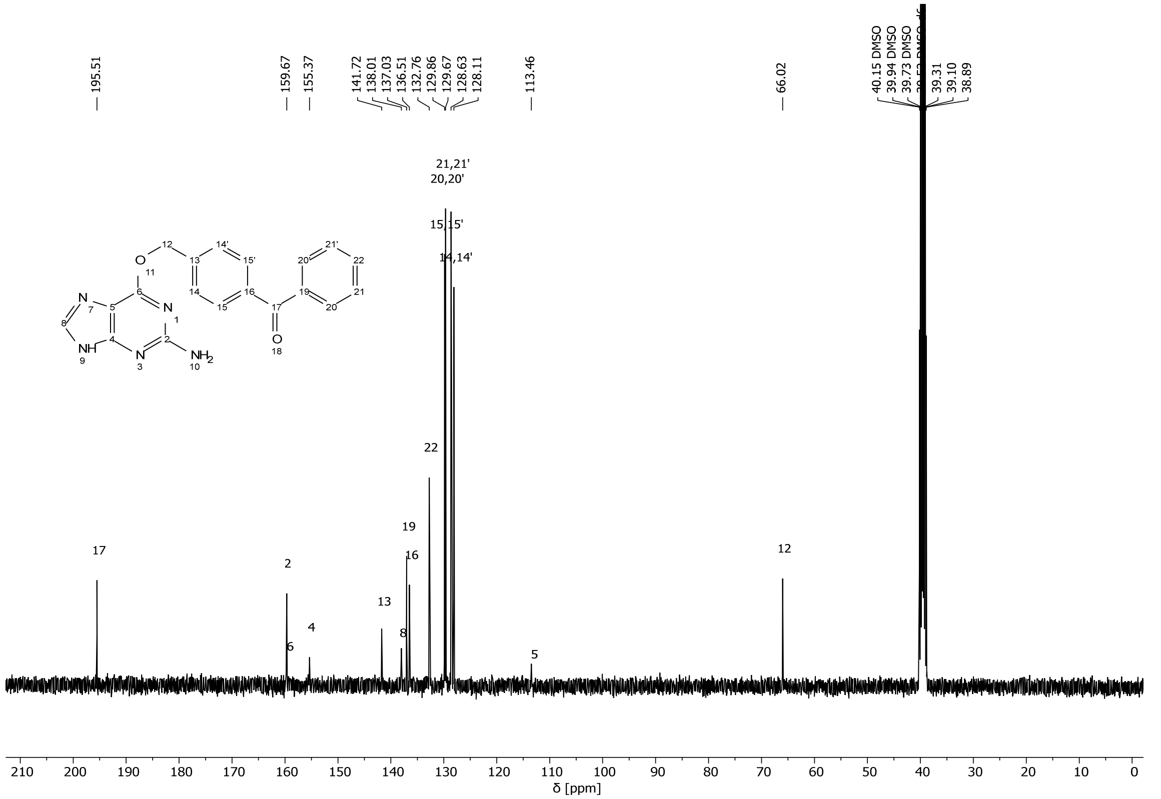

^{13}C NMR (101 MHz, DMSO-d_6) of compound **BG-Bz (9)**.

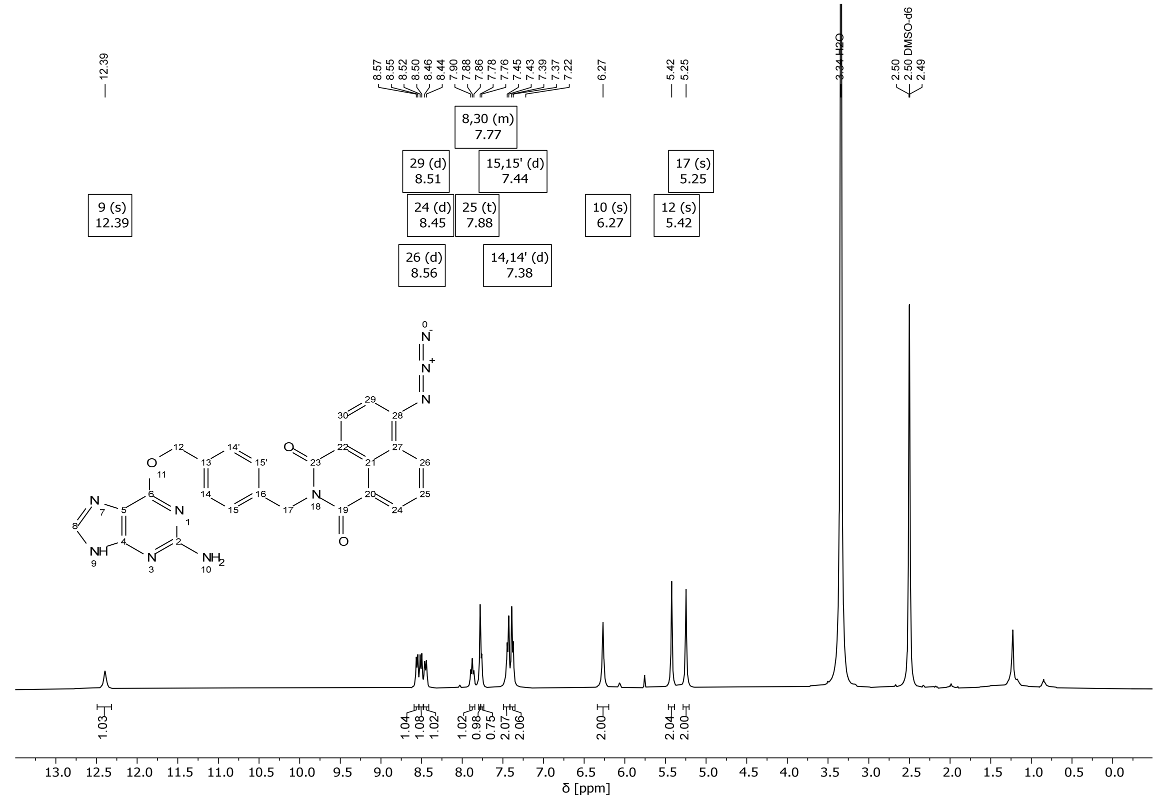

^1H NMR (400 MHz, DMSO-d_6) of compound **BG-AzNP (10)**.

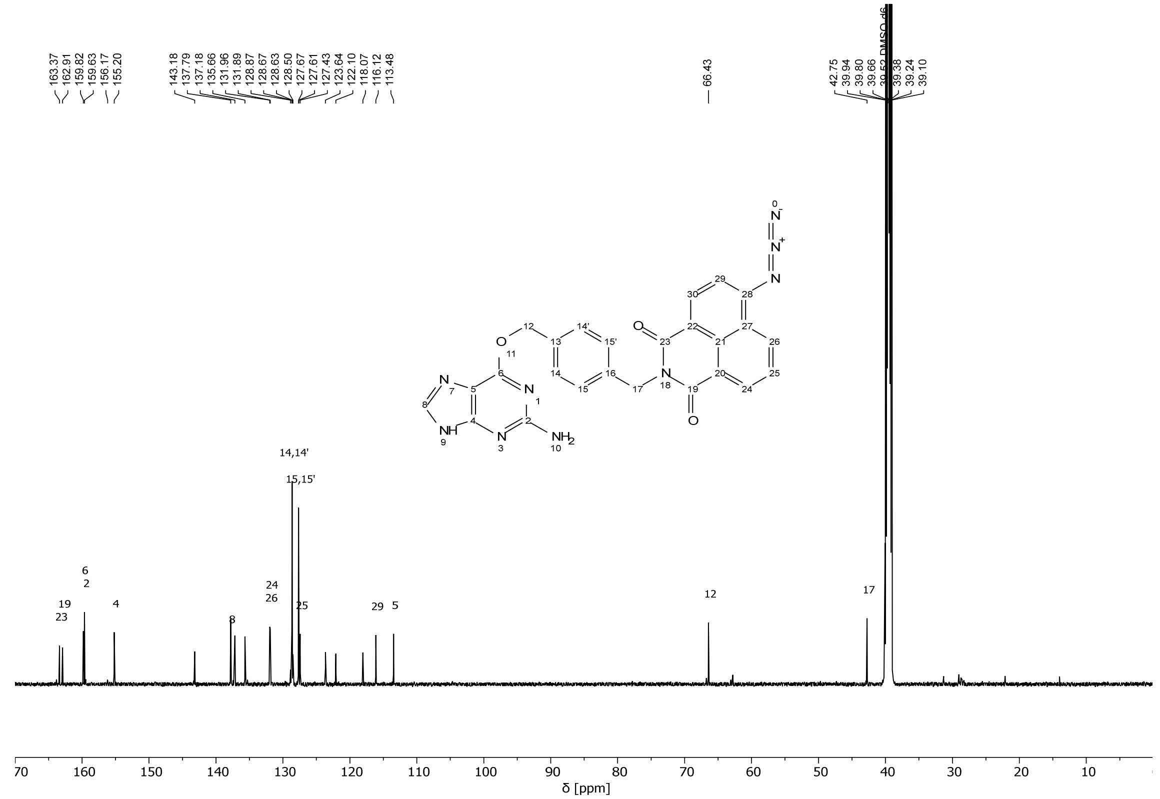

^{13}C NMR (101 MHz, DMSO-d_6) of compound **BG-AzNP (10)**.

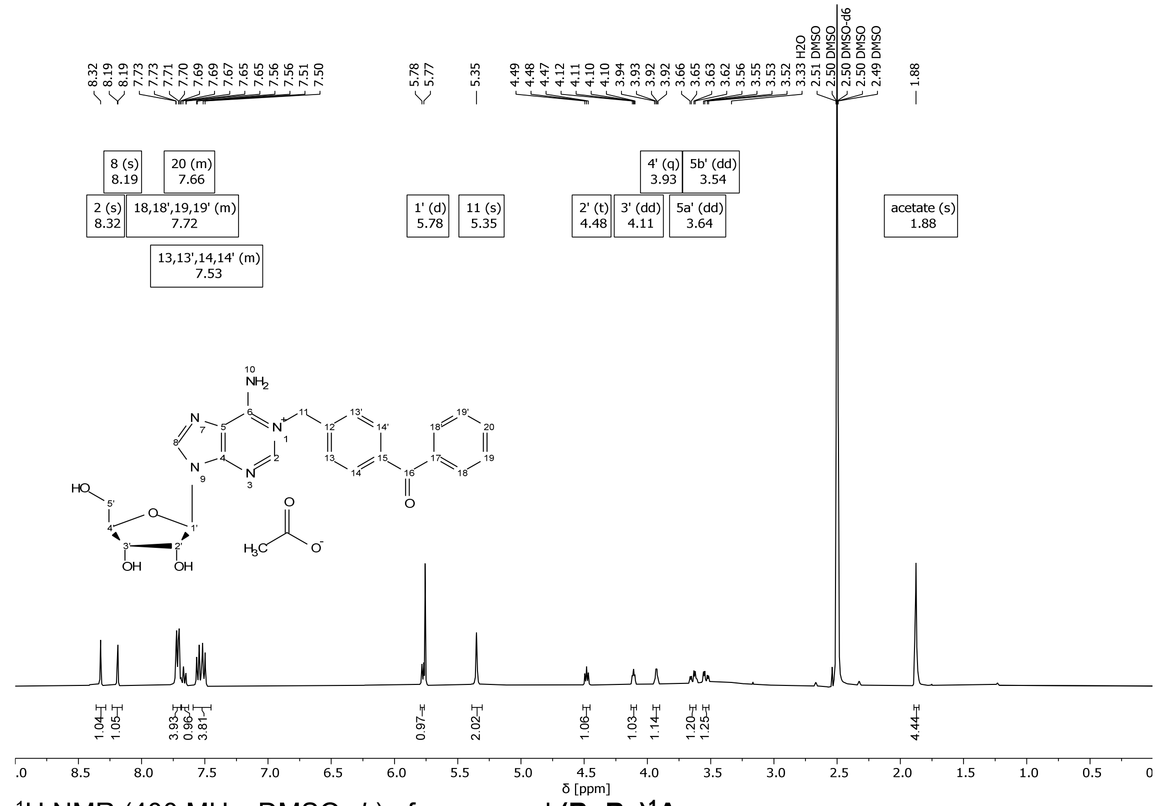

^{1}H NMR (400 MHz, DMSO-d_6) of compound **(BnBz)[1]A**.

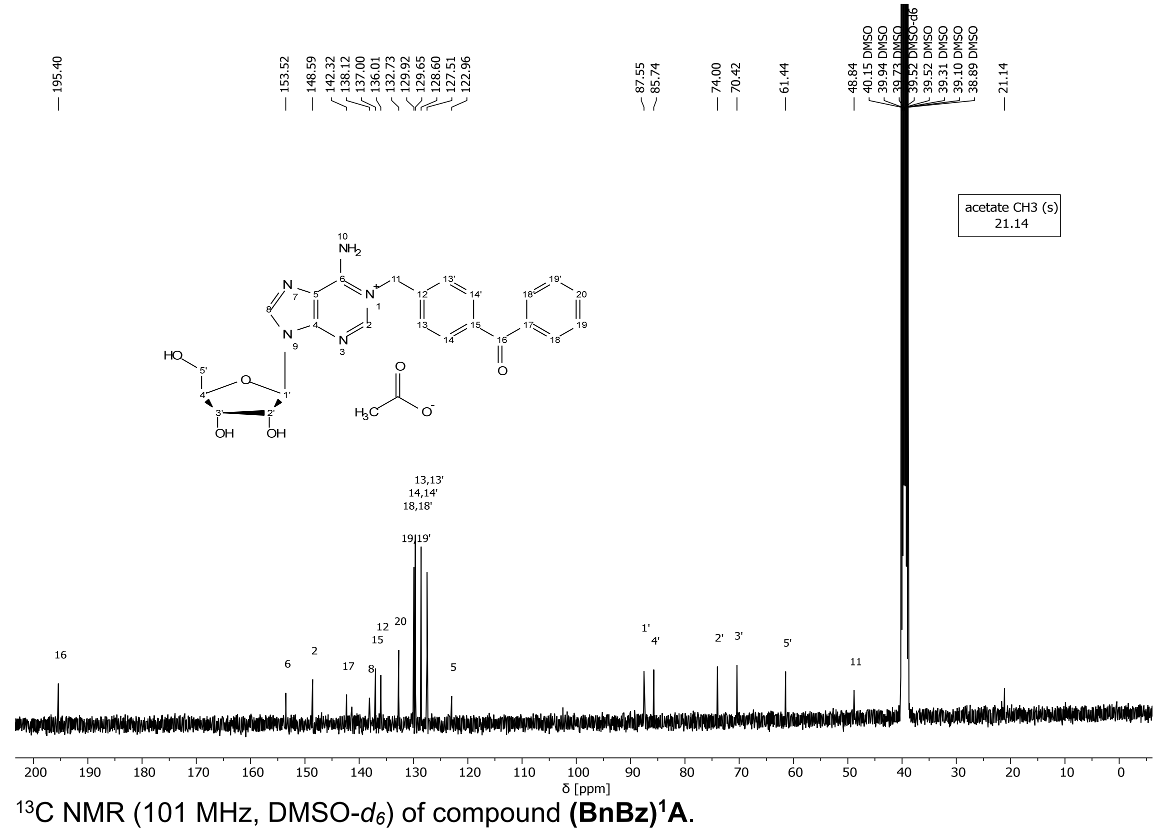

^{13}C NMR (101 MHz, DMSO-d_6) of compound **(BnBz)[1]A**.

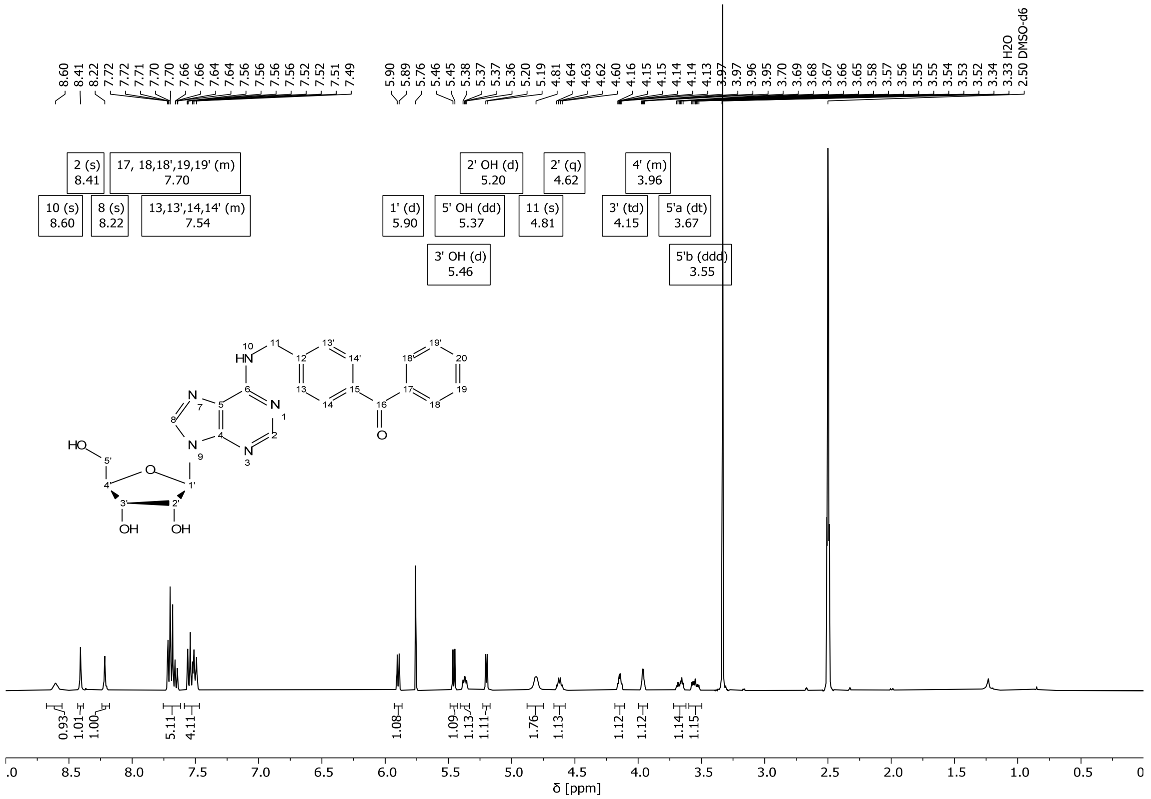

^{1}H NMR (400 MHz, DMSO-d_6) of compound **(BnBz)⁶A**.

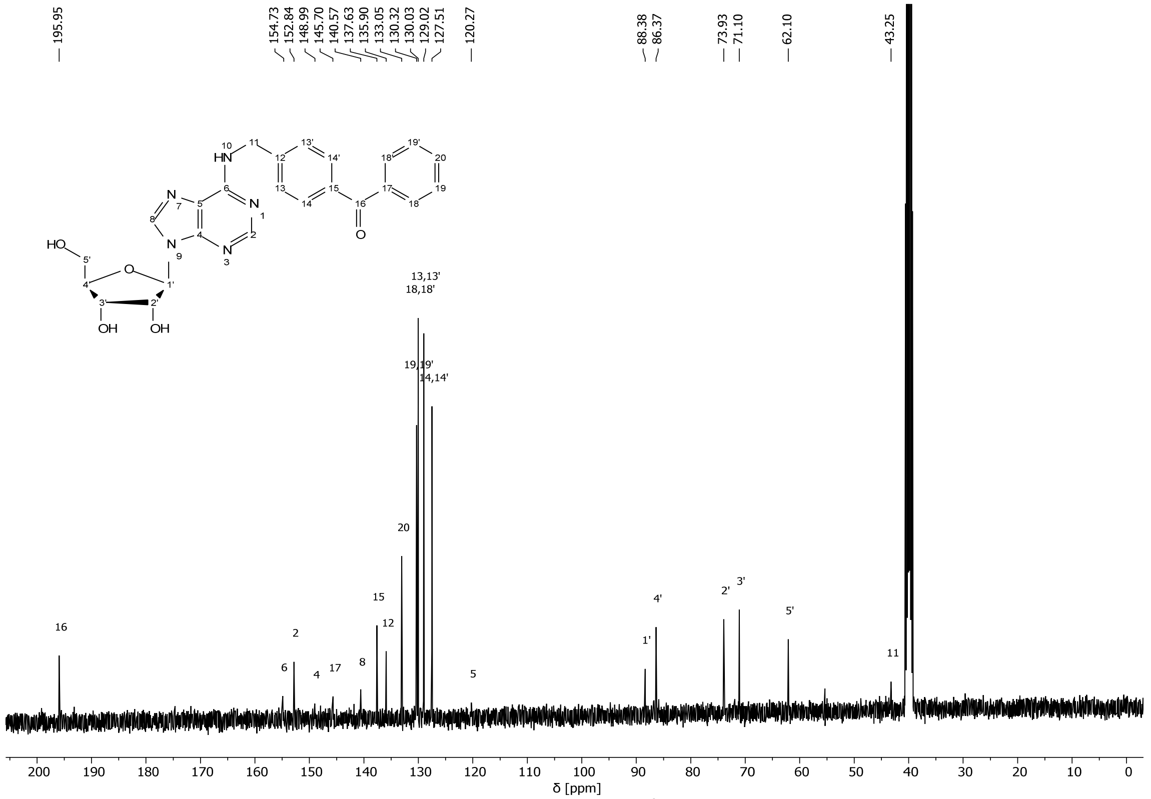

^{13}C NMR (101 MHz, DMSO-d_6) of compound **(BnBz)⁶A**.

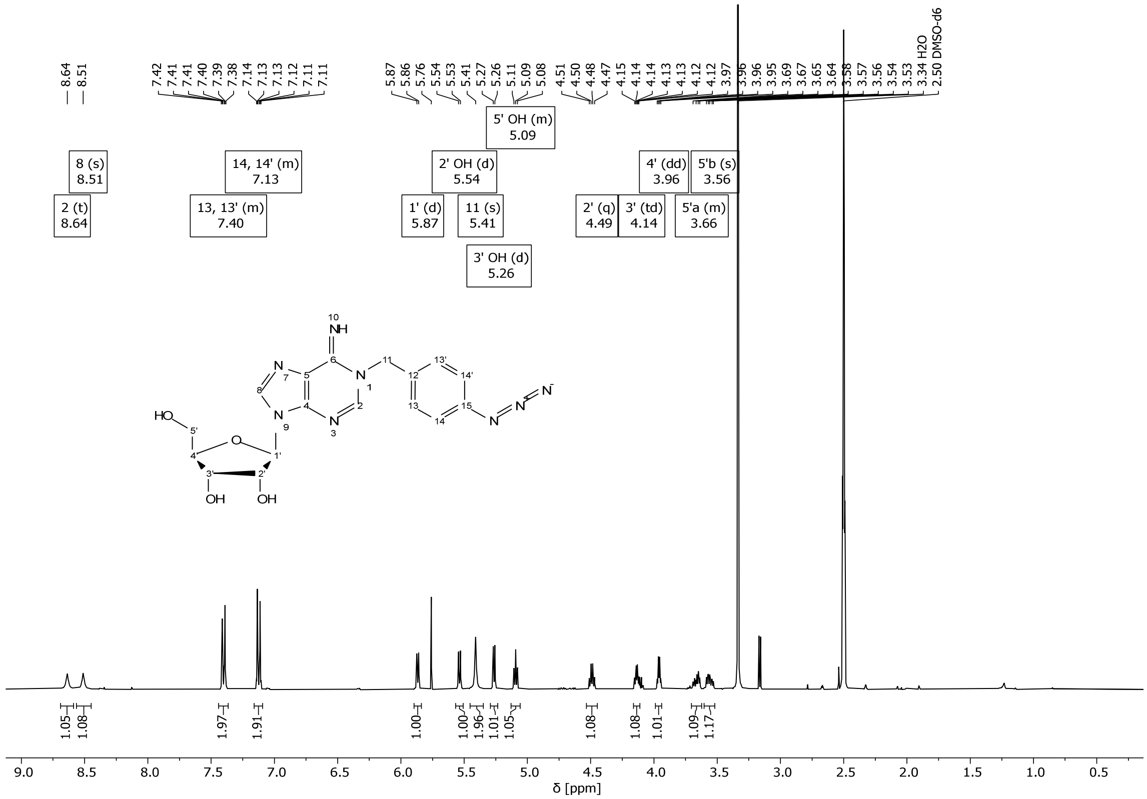

^{1}H NMR (400 MHz, DMSO-*d6*) of compound **(BnN$_3$)^{1}A**.

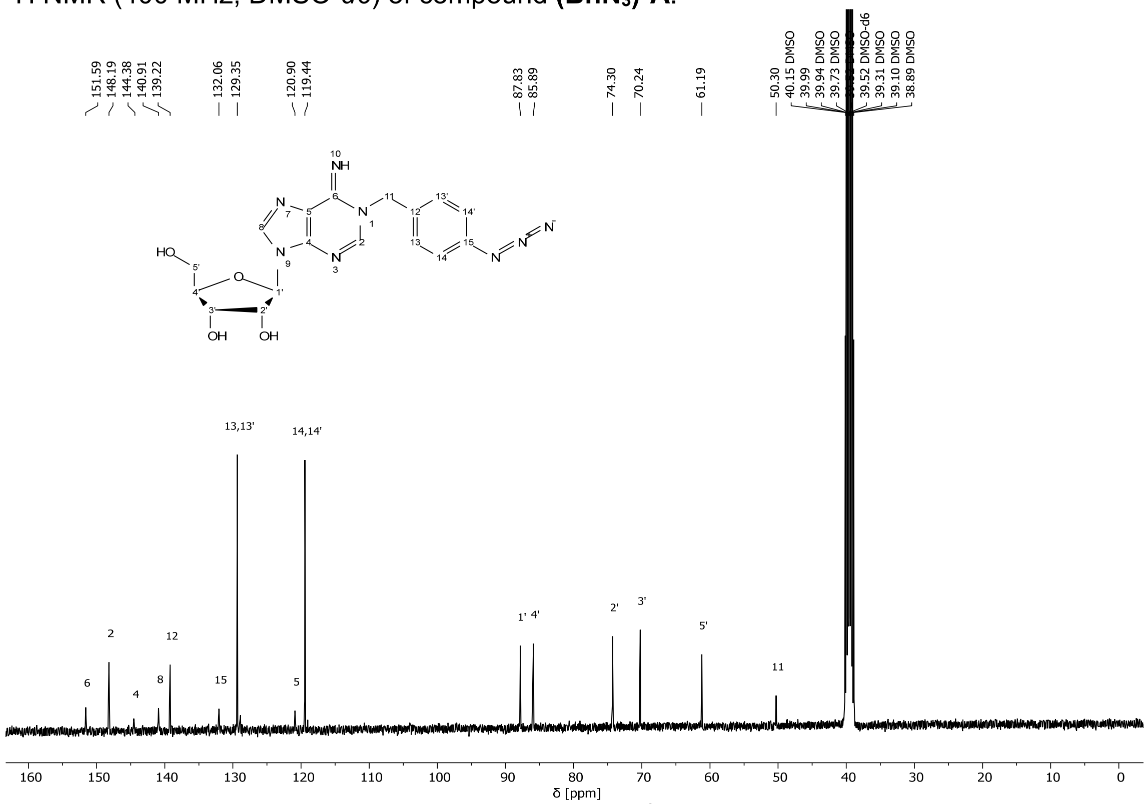

^{13}C NMR (101 MHz, DMSO-*d$_6$*) of compound **(BnN$_3$)^{1}A**.

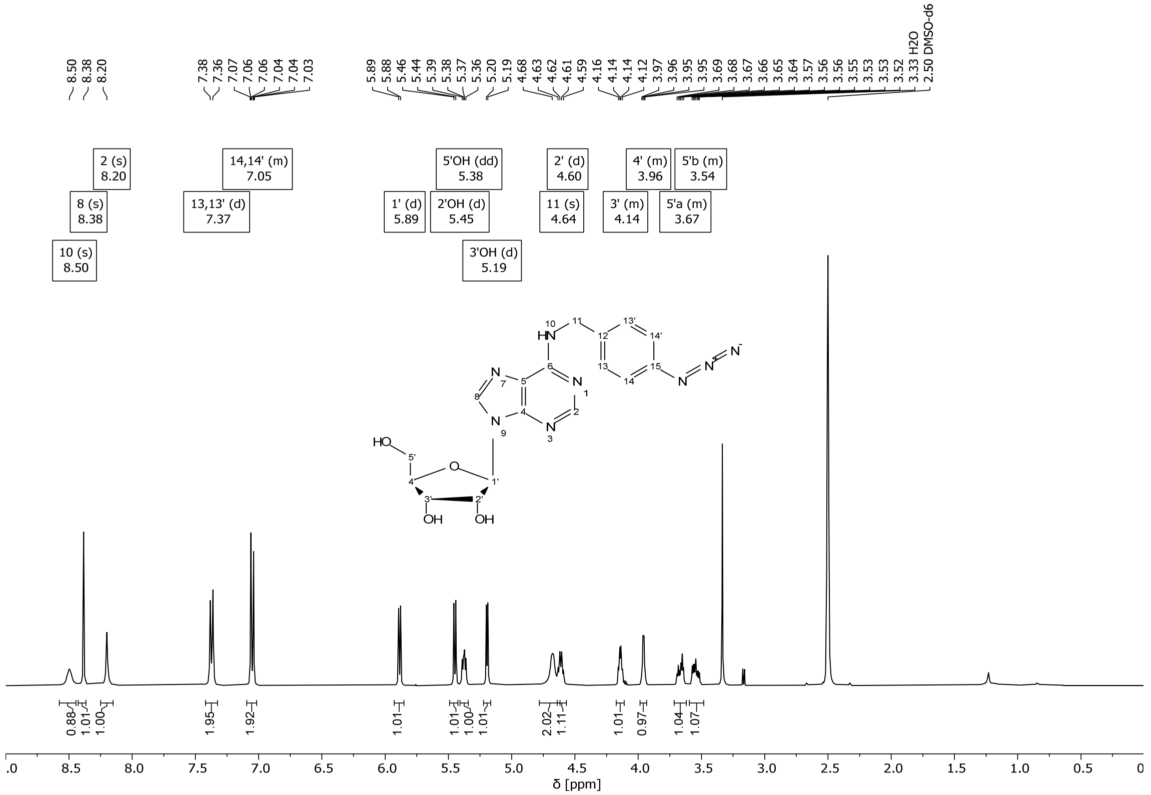

^{1}H NMR (400 MHz, DMSO-*d6*) of compound **$(BnN_3)^6$A**.

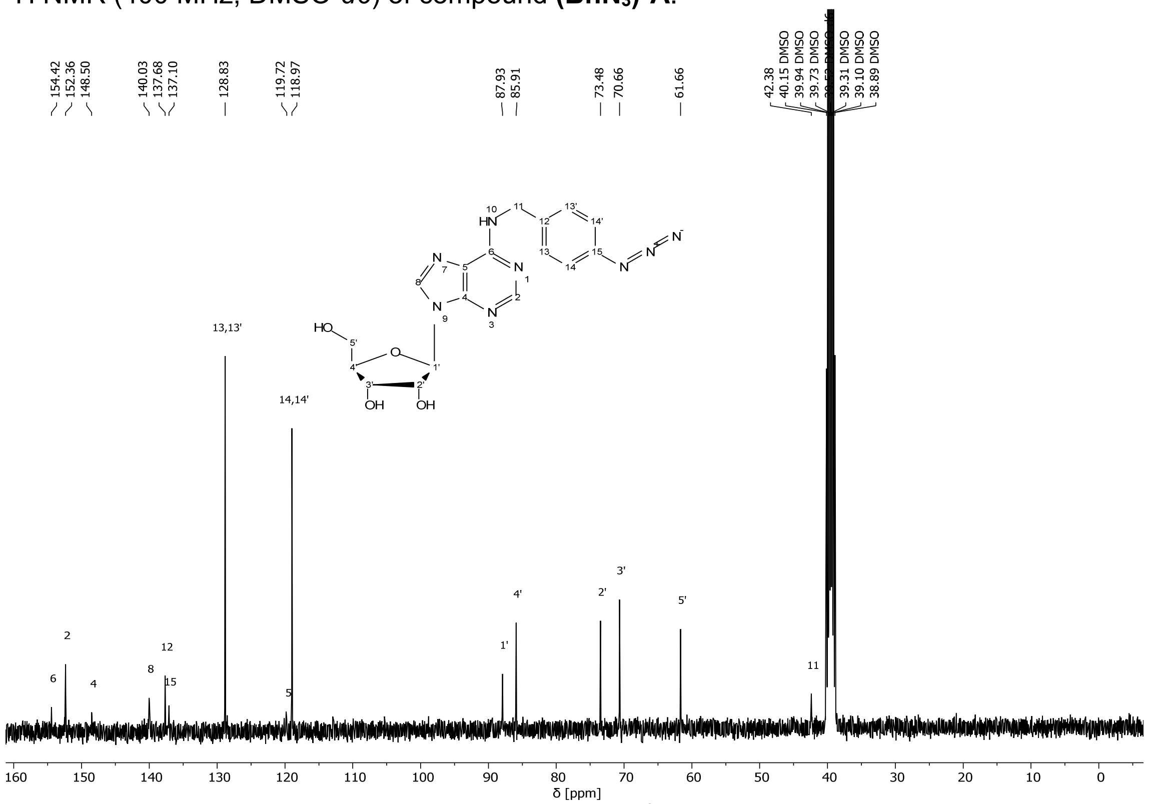

^{13}C NMR (101 MHz, DMSO-*d_6*) of compound **$(BnN_3)^6$A**.

14. HR-ESI-MS spectra of isolated alkylated RNA

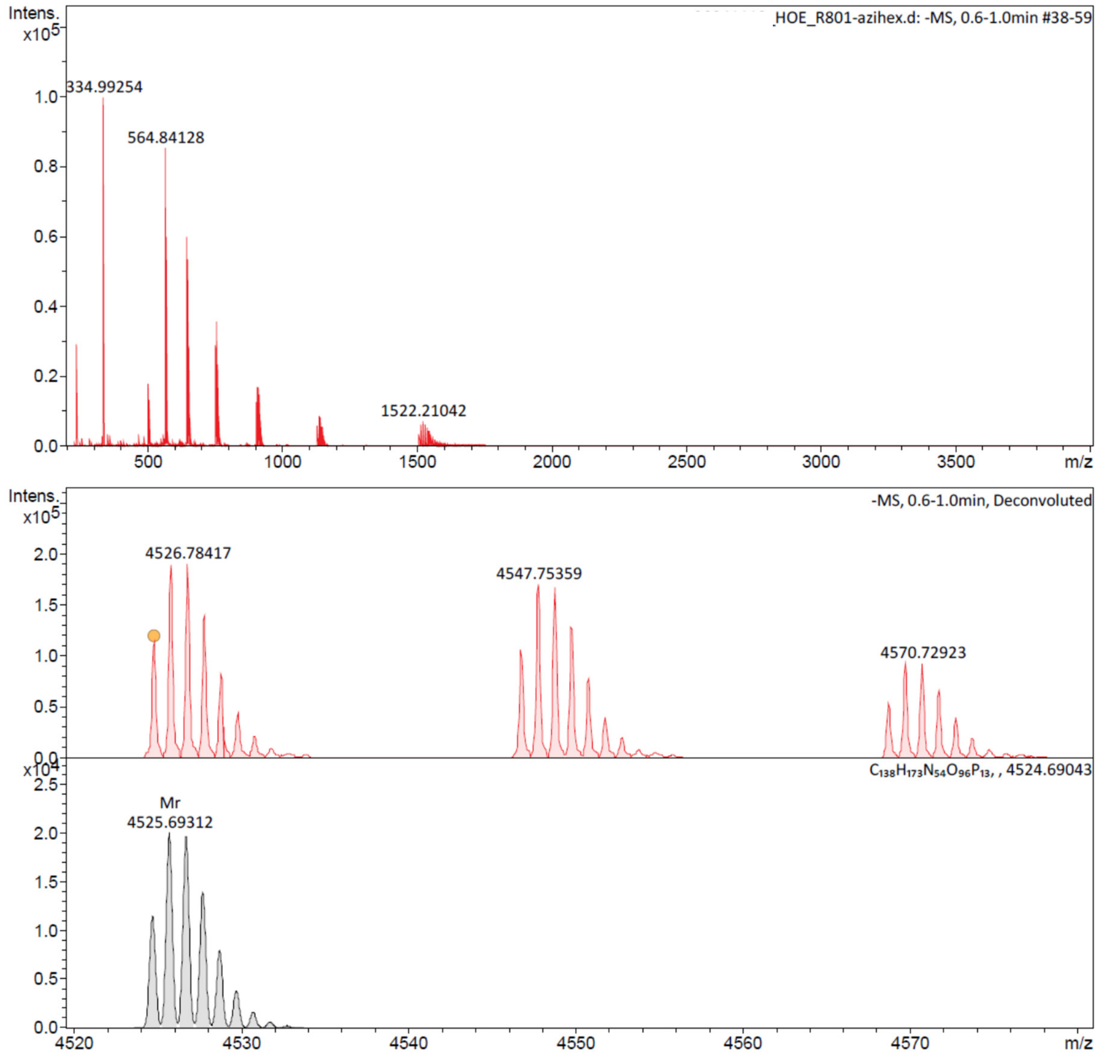

Meas. m/z	#	Ion Formula	m/z	err [ppm]	mSigma	# mSigma	Score	rdb	e⁻ Conf	N-Rule
4524.76559	1	C138H173N54O96P13	4524.69098	-16.49	29.9	1	100.00	86.0	odd	ok

HR-ESI-MS of R2-azihex

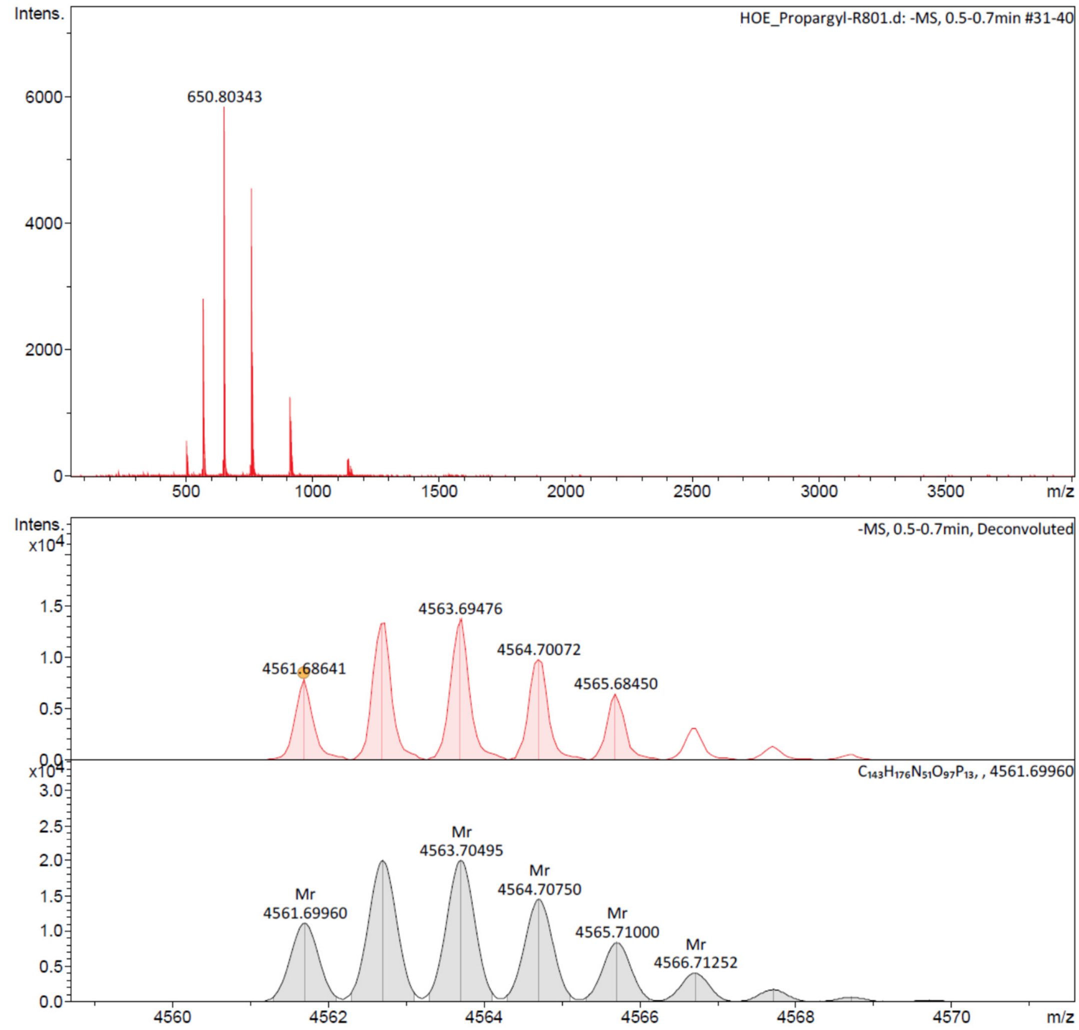

Meas. m/z	#	Ion Formula	m/z	err [ppm]	mSigma	# mSigma	Score	rdb	e¯ Conf	N-Rule
4561.68641	1	C143H176N51O97P13	4561.69960	2.89	82.3	1	100.00	88.0	odd	ok

HR-ESI-MS of R2-Bnprop

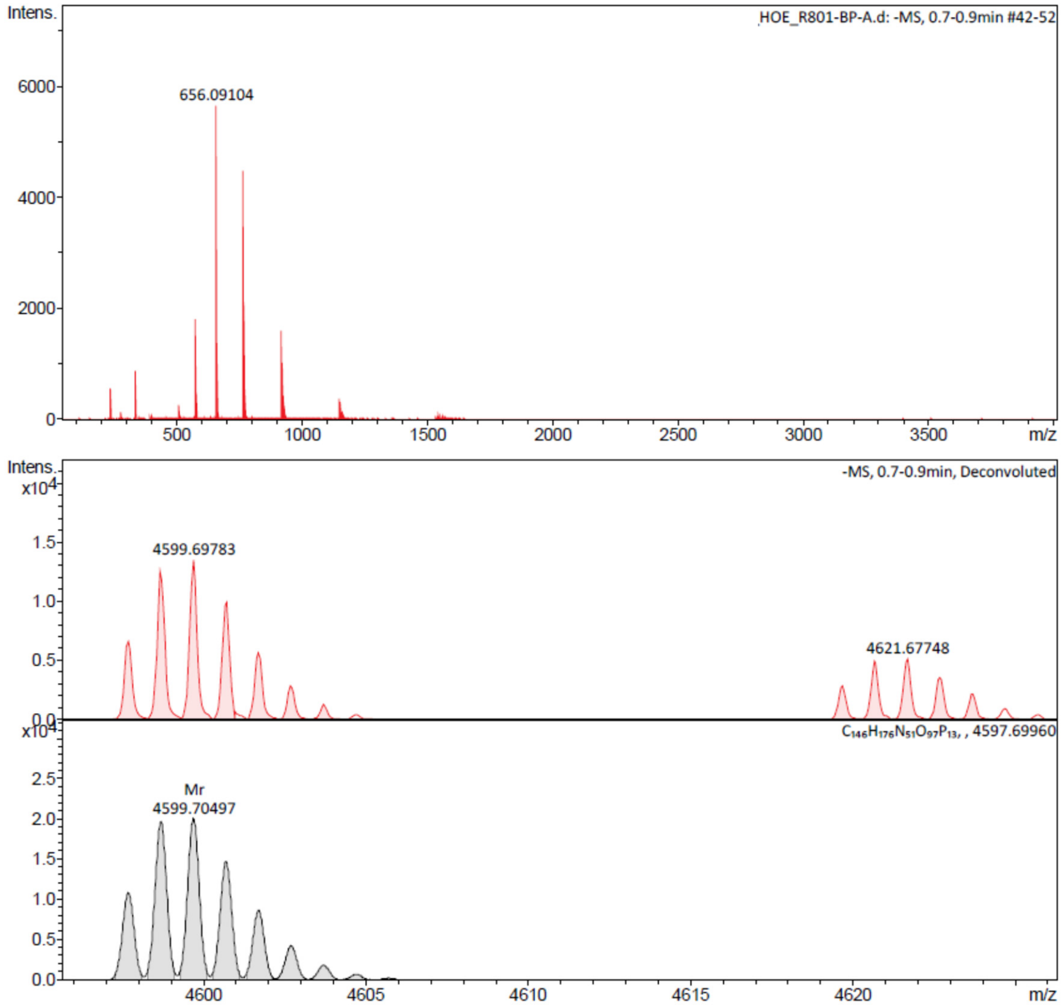

Meas. m/z	#	Ion Formula	m/z	err [ppm]	mSigma	# mSigma	Score	rdb	e⁻ Conf	N-Rule
4597.68489	1	C146H176N51O97P13	4597.70015	3.32	30.3	1	100.00	91.0	odd	ok

HR-ESI-MS of R2-BnBz

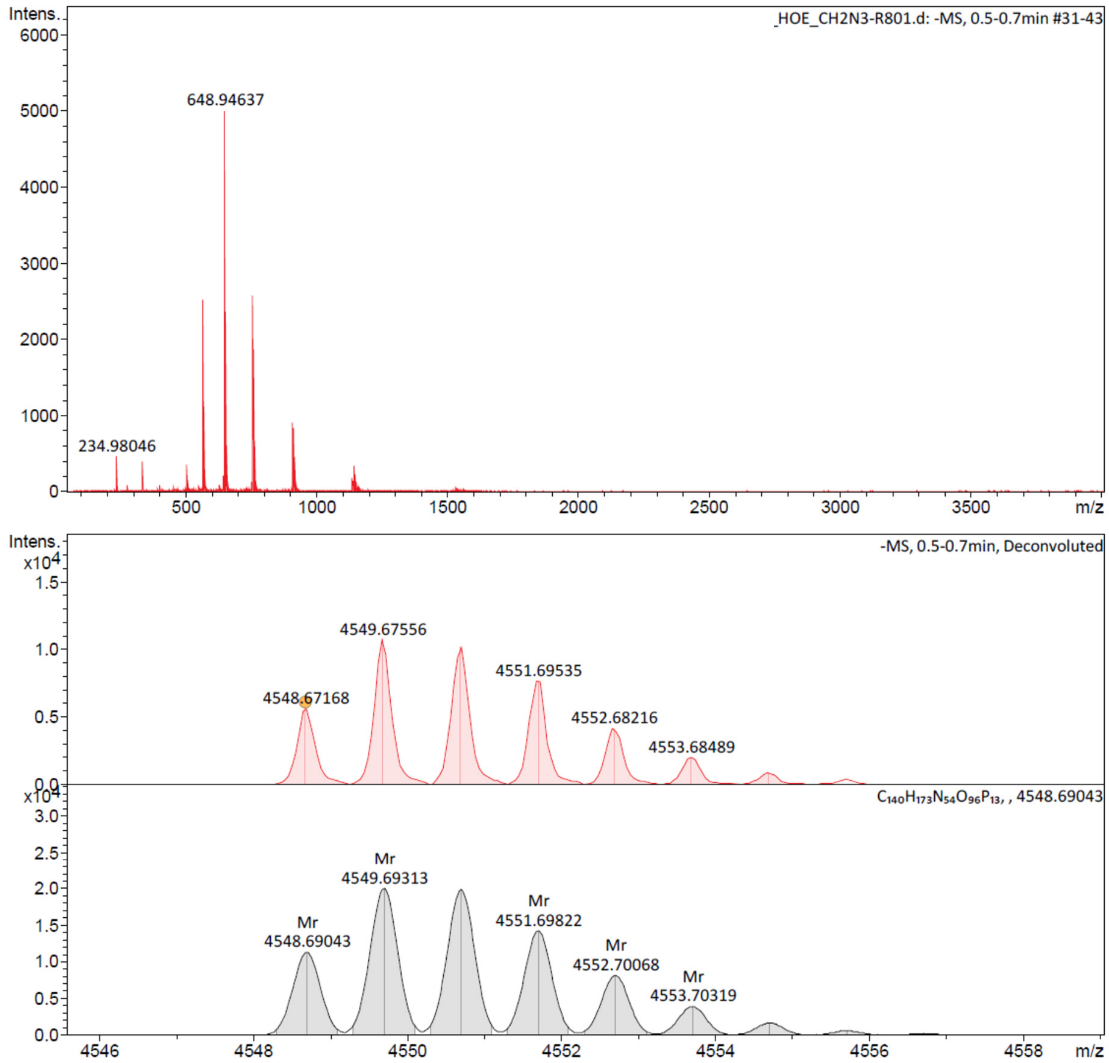

Meas. m/z	#	Ion Formula	m/z	err [ppm]	mSigma	# mSigma	Score	rdb	e‾ Conf	N-Rule
4548.67168	1	C140H173N54O96P13	4548.69043	4.12	25.7	1	100.00	88.0	odd	ok

HR-ESI-MS of R2-BnCH2N3

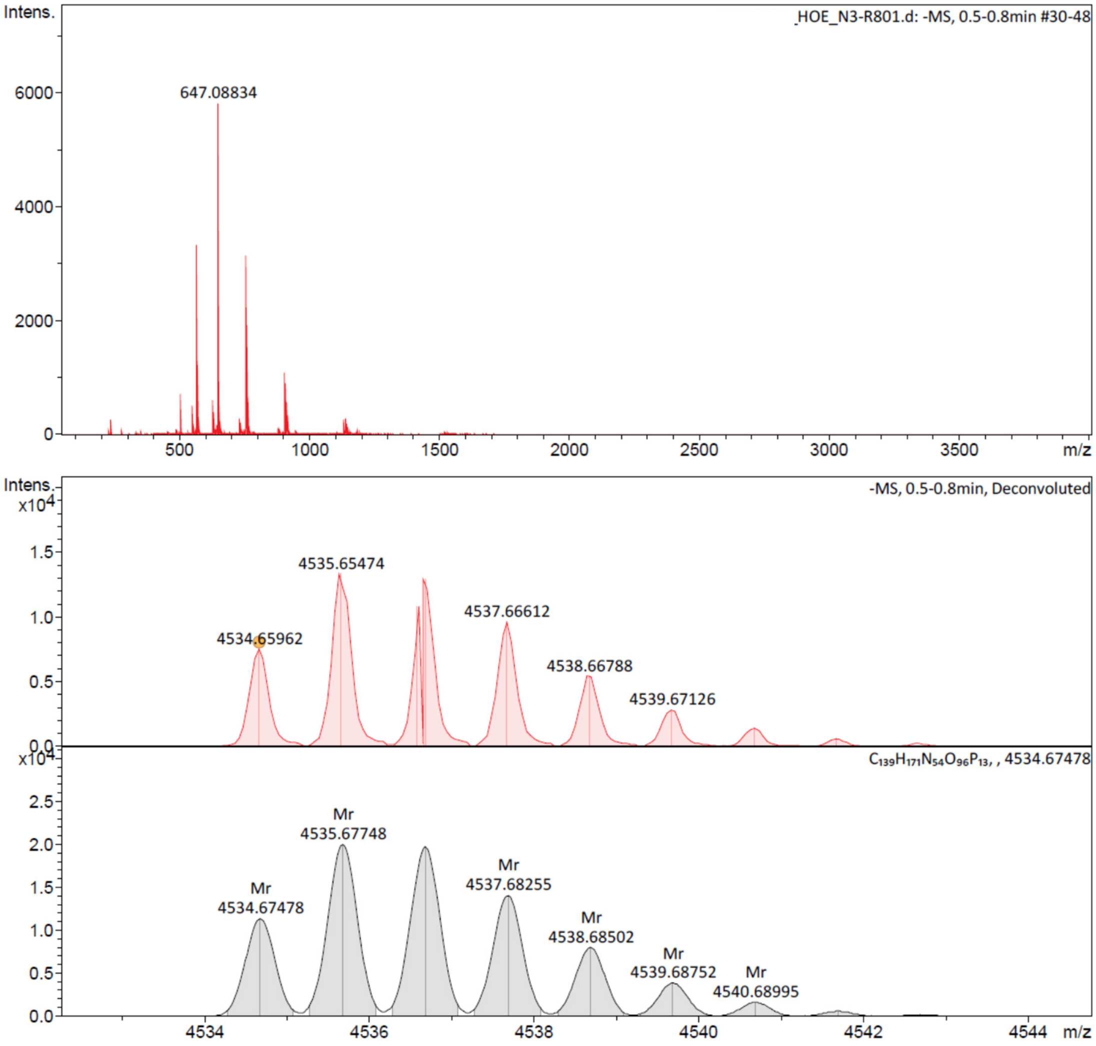

Meas. m/z	#	Ion Formula	m/z	err [ppm]	mSigma	# mSigma	Score	rdb	e¯ Conf	N-Rule
4534.65962	1	C139H171N54O96P13	4534.67533	3.46	16.8	1	100.00	88.0	odd	ok

HR-ESI-MS of R2-BnN3

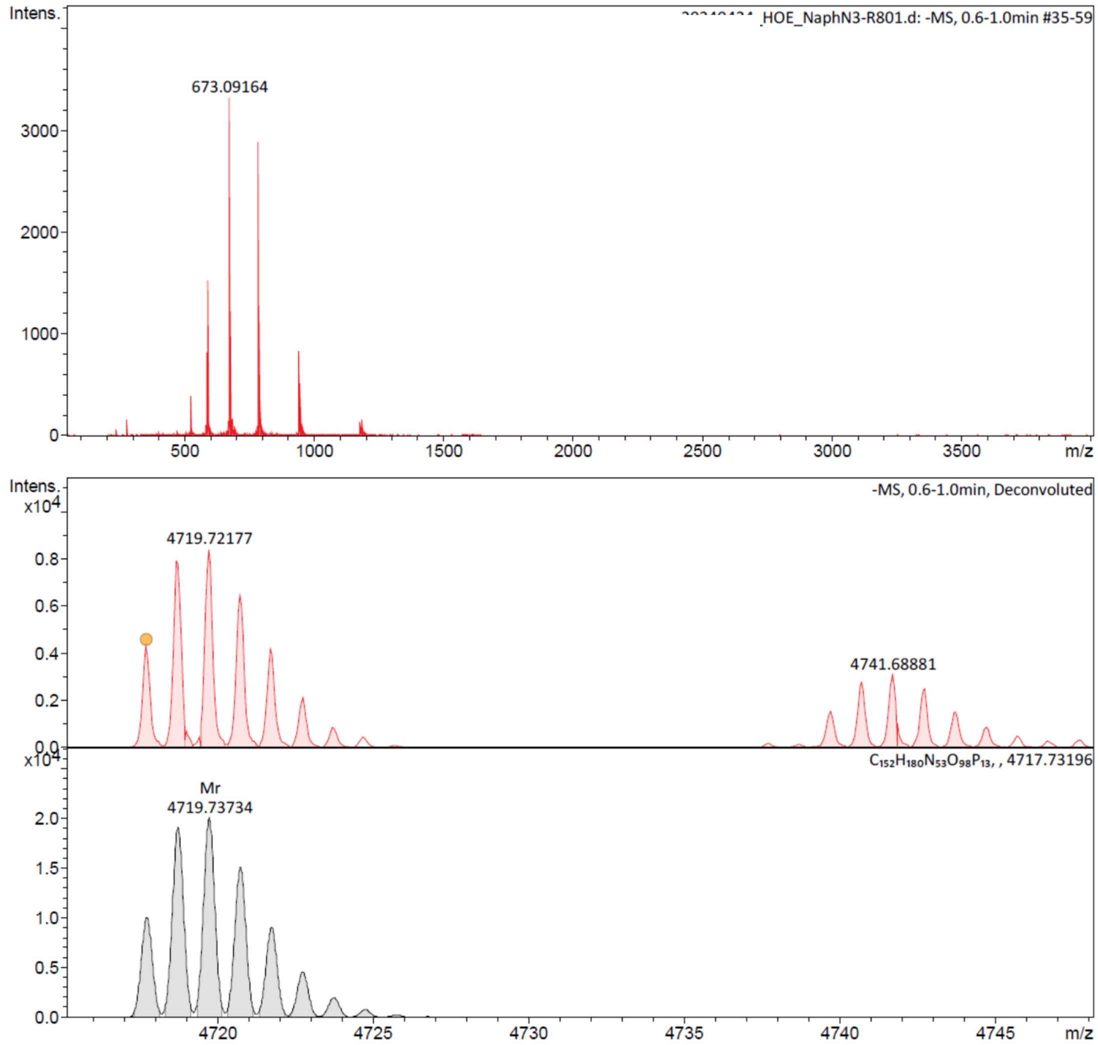

Meas. m/z	#	Ion Formula	m/z	err [ppm]	mSigma	# mSigma	Score	rdb	e¯ Conf	N-Rule
4717.70016	1	C152H180N53O98P13	4717.73251	6.86	23.3	1	100.00	96.0	odd	ok

HR-ESI-MS of R2-BnAzNP (-N2)

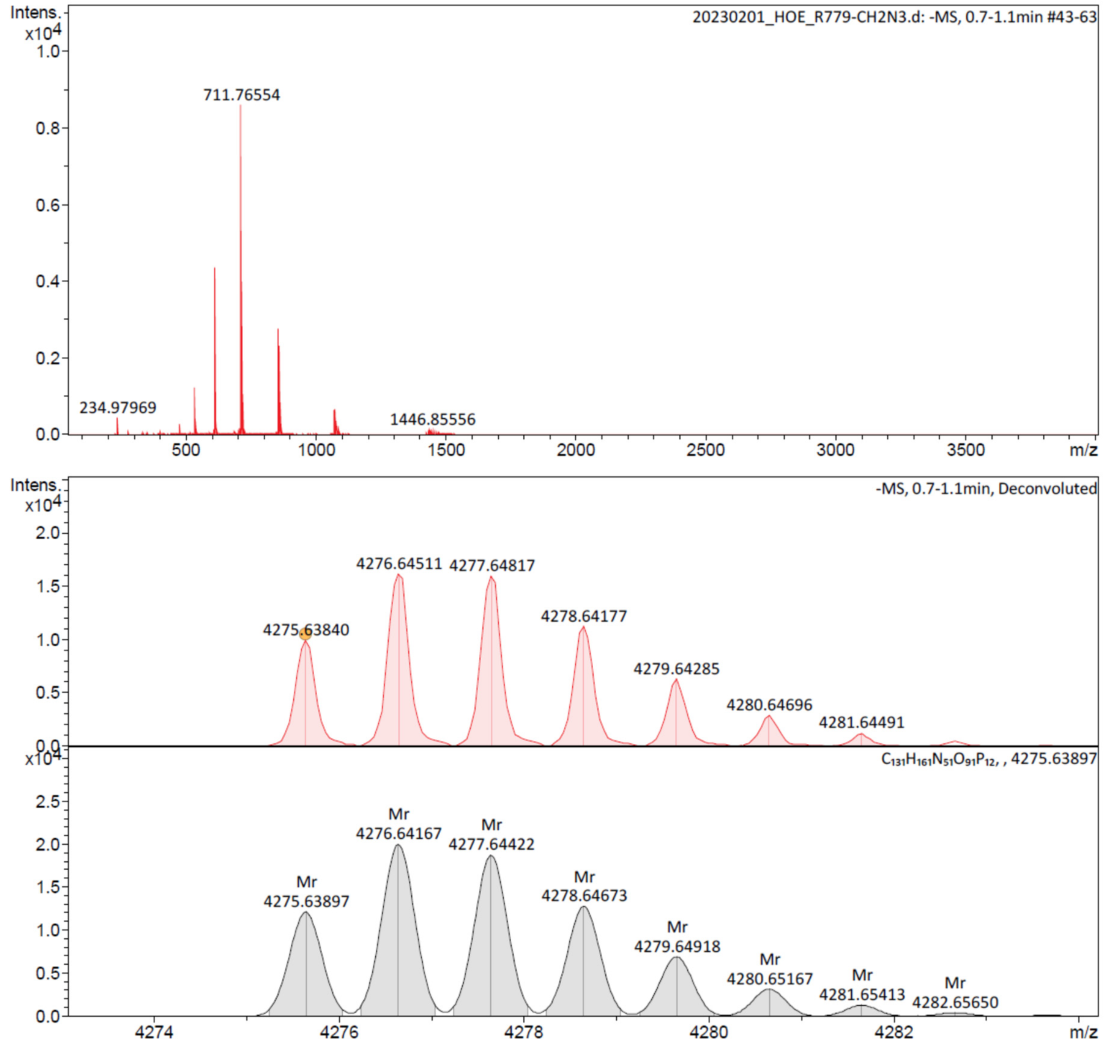

Meas. m/z	#	Ion Formula	m/z	err [ppm]	mSigma	# mSigma	Score	rdb	e⁻ Conf	N-Rule
4275.63840	1	C131H161N51O91P12	4275.63897	0.14	34.8	1	100.00	83.0	odd	ok

HR-ESI-MS of R5-BnCH2N3

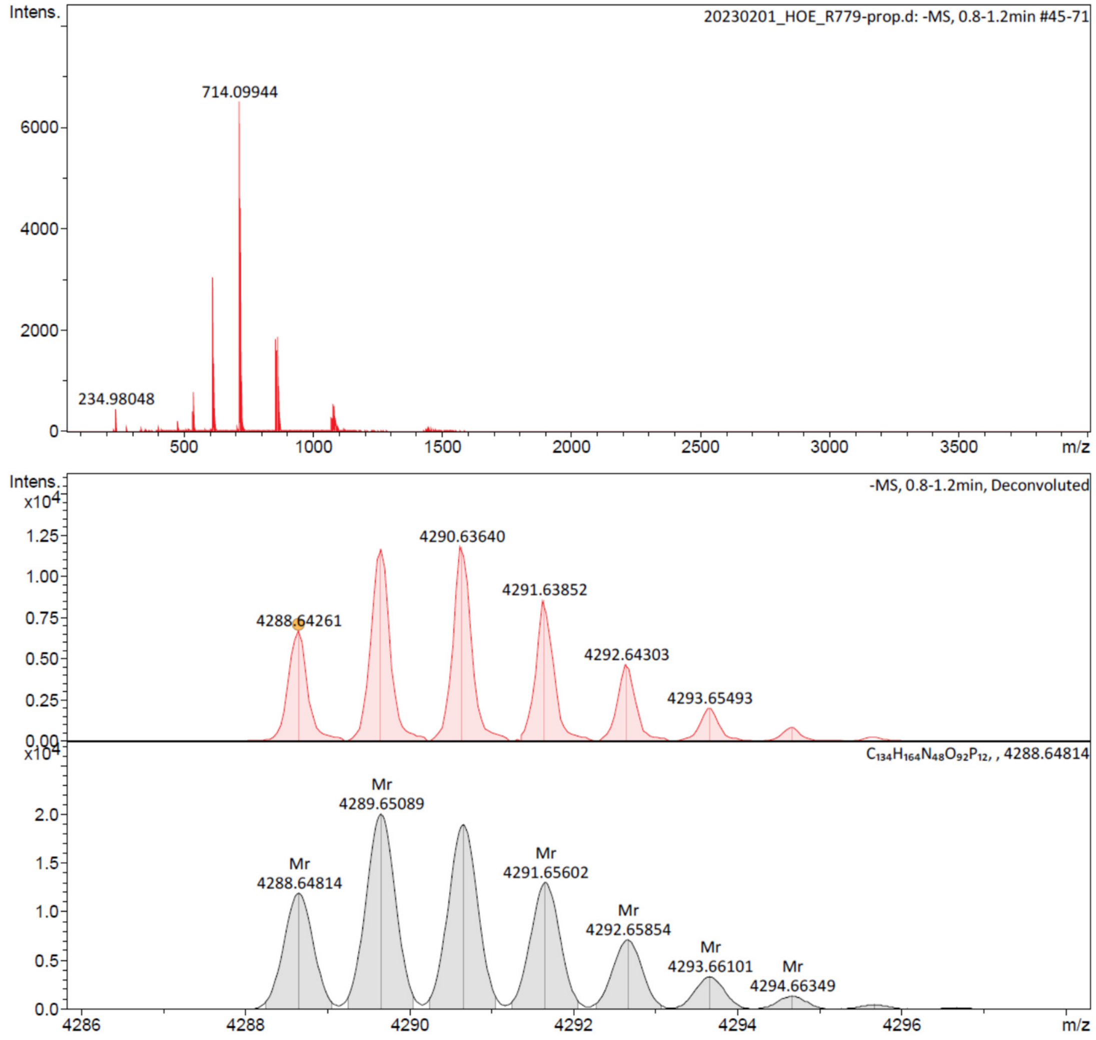

Meas. m/z	#	Ion Formula	m/z	err [ppm]	mSigma	# mSigma	Score	rdb	e⁻ Conf	N-Rule
4288.64261	1	C134H164N48O92P12	4288.64814	1.29	37.5	1	100.00	83.0	odd	ok

HR-ESI-MS of R5-BnPr

References

[1] D. A. Depke, C. P. Konken, L. Rösner, S. Hermann, M. Schäfers, A. Rentmeister. *Chem. Commun.* **2021**, *57*, 9850 – 9853.

[2] G. Lukinavičius, M. Tomkuvienė, V. Masevičius, S. Klimašauskas, *ACS Chem. Biol.* **2013**, *8*, 1134 – 1139.

[3] A.L.Z. Lee, Z.X. Voo, W.Chin, R. J. Ono, C. Yang, S. Gao, J. L. Hedrick, Y. Y. Yang, *ACS Appl. Mater. Interfaces* **2018** *10*, 13274 – 13282.

[4] M. Belkheira, D. El Abed, J.-M. Pons, C. Bressy, *Chem. Eur. J.* **2011**, *17*, 12917 – 12921.

[5] R. Tang, J. Zhu, Z. Yan, L. Yiming, *Front. Chem. China*. **2006**, *1*, 174 – 178.

[6] McCutcheon DC, Lee G, Carlos A, Montgomery JE, Moellering RE. *J Am Chem Soc.* **2020**, *42*, 146 – 153.

[7] S. A. McNelles, J. L. Pantaleo, A. Adronov, *Org. Process Res. Dev.* **2019**, *23*, 2740 – 2745.

[8] Bandela, A. K.; Bandaru, S.; Rao, C. P. A *Chem. – Eur. J.* **2015**, *21*, 13364 – 13374.

[9] Broichhagen, J.; Damijonaitis, A.; Levitz, J.; Sokol, K. R.; Leippe, P.; Konrad, D.; Isacoff, E. Y.; Trauner, D. *ACS Cent. Sci.* **2015**, *1*, 383 – 393.

[10] Montoya LA, Pluth MD. *Anal Chem.* **2016**, *88*, 5769 – 5774.

[11] Anhäuser, L.; Klöcker, N.; Muttach, F.; Mäsing, F.; Spacek, P.; Studer, A.; Rentmeister, A. *Angew. Chem., Int. Ed.* **2020**, *59*, 3161 – 3165.

[12] Pagnozzi, D.; Pala, N.; Biosa, G.; Dallocchio, R.; Dessì, A.; Singh, P. K.; Rogolino, D.; Di Fiore, A.; De Simone, G.; Supuran, C. T.; Sechi, M. *ACS Med. Chem. Lett.* **2022**, *13*, 271 – 277.

[13] S. Uno, H. Kimura, M. Murai, H. Miyoshi, *J. Biol. Chem.* **2019**, *294*, 679 – 696.

[14] H. Liu, T. Zeng, C. He, V. H. Rawal, H. Zhou, B. C. Dickinson, *Acs Chem Biol* **2022**, *17*, 1334 – 1342.

[15] Griffin, R. J.; Arris, C. E.; Bleasdale, C.; Boyle, F. T.; Calvert, A. H.; Curtin, N. J.; Dalby, C.; Kanugula, S.; Lembicz, N. K.; Newell, D. R.; Pegg, A. E.; Golding, B. T. *J. Med. Chem.* **2000**, *43*, 4071 – 4083.

[16] Zhang, C.-J.; Li, L.; Chen, G. Y. J.; Xu, Q.-H.; Yao, S. *Q. Org. Lett*. **2011**, *13*, 4160 – 4163.

[17] Höbartner, C.; Kreutz, C.; Flecker, E.; Ottenschläger, E.; Pils, W.; Grubmayr, K.; Micura, R. Monatsh. Chem. 2003, 134, 851–873.
